# Dynamic transcription programs during ES cell differentiation towards mesoderm in serum versus serum-free^BMP4 ^culture

**DOI:** 10.1186/1471-2164-8-365

**Published:** 2007-10-10

**Authors:** Stephen J Bruce, Brooke B Gardiner, Les J Burke, M Milena Gongora, Sean M Grimmond, Andrew C Perkins

**Affiliations:** 1 Institute for Molecular Bioscience, University of Queensland, Brisbane, Queensland, Australia; 2 Wesley Research Institute, Queensland, Australia; 3 ARC Special Research Centre in Functional and Applied Genomics, Australia; 4 Australian Stem Cell Centre, Melbourne, Australia

## Abstract

**Background:**

Expression profiling of embryonic stem (ES) cell differentiation in the presence of serum has been performed previously. It remains unclear if transcriptional activation is dependent on complex growth factor mixtures in serum or whether this process is intrinsic to ES cells once the stem cell program has been inactivated. The aims of this study were to determine the transcriptional programs associated with the stem cell state and to characterize mesoderm differentiation between serum and serum-free culture.

**Results:**

ES cells were differentiated as embryoid bodies in 10% FBS or serum-free media containing BMP4 (2 ng/ml), and expression profiled using 47 K Illumina(R) Sentrix arrays. Statistical methods were employed to define gene sets characteristic of stem cell, epiblast and primitive streak programs. Although the initial differentiation profile was similar between the two culture conditions, cardiac gene expression was inhibited in serum whereas blood gene expression was enhanced. Also, expression of many members of the *Kruppel-like factor *(KLF) family of transcription factors changed dramatically during the first few days of differentiation. KLF2 and KLF4 co-localized with OCT4 in a sub-nuclear compartment of ES cells, dynamic changes in KLF-DNA binding activities occurred upon differentiation, and strong bio-informatic evidence for direct regulation of many stem cell genes by KLFs was found.

**Conclusion:**

Down regulation of stem cell genes and activation of epiblast/primitive streak genes is similar in serum and defined media, but subsequent mesoderm differentiation is strongly influenced by the composition of the media. In addition, KLF family members are likely to be important regulators of many stem cell genes.

## Background

Embryonic stem (ES) cells isolated from the inner cell mass of the early mammalian blastocyst-stage embryo retain pluripotency when cultured on mouse embryonic fibroblasts (MEFs) in the presence of leukemia inhibitory factor (LIF) [1]. In the absence of LIF and MEF attachment, ES cells spontaneously differentiate into multi-cellular aggregates termed embryoid bodies (EBs). Although the spatial complexity of organogenesis is not established during EB maturation, the dynamics of gene expression closely mimic those which characterize early stages of mouse development [2-5]. Thus, ES cell differentiation is an excellent model system for the discovery of genes involved in developmental processes.

Many studies have attempted to identify genes that define the stem cell state by mining for genes co-expressed in ES cells and various other stem cell populations such as hematopoietic and neural stem cells [6,7], or ES cells and trophoblast stem cells [8]. Some of these studies have been criticized because they were unable to define a similar cohort of stem cell genes [9]. Also, this approach is likely to find co-expression of 'housekeeping' genes that are irrelevant to the stem cell state. More recently, dynamic expression profiling during ES cell differentiation has been undertaken using various EB culture techniques [10,11] and the addition of exogenous growth factors [7]. Most of these studies followed differentiation for a short time period (up to 6–8 days) due to inherent limitations of liquid or adherent cultures cell differentiation systems.

Various platforms have been used to determine gene expression profiles, including differential display, SAGE [12] and slide micro-arrays, such as those provided by Affymetrix, Agilent and Compugen. In this study we employed Illumina^® ^Sentrix Mouse-6 oligo bead arrays [13], which have ~47 K probes that are largely based on the MEEBO set of transcripts, plus ESTs discovered as part of the FANTOM2 transcriptome project [14], some alternatively employed exons, and a small subset of non-coding transcripts. This is a new, rich and sensitive platform for expression profiling [15].

ES cells were differentiated in methylcellulose cultures, which allowed consistent EB development over 16 days in either serum [2,16] or defined serum-free media containing BMP4 (2 ng/ml), termed serum-free^B4L ^hereafter [17]. The dynamics of loss of stem cell gene expression and activation of epiblast and primitive streak gene expression programs were similar in serum and serum-free^B4L ^but QT clustering revealed a significant difference in mesoderm outcomes from day 6 onwards. In particular, cardiac gene expression was greater in serum-free^B4L ^whereas activation of the blood program was enhanced in serum.

High expression levels of Kruppel-like factor (KLF) family members: *Klf2*, *Klf4*, *Klf5 *and *Klf9 *was detected in undifferentiated ES cells, and confirmed by quantitative real time RT-PCR, indirect immuno-fluorescence and DNA-binding assays. Expression was down regulated rapidly upon differentiation and a different set of *Klfs: Klf3*, *Klf16 *and *Klf1/Eklf*, were activated upon ES cell differentiation. Also, KLF-binding sites were markedly over represented in the proximal promoters of most stem cell specific genes, suggesting possible direct regulation by KLFs. We suggest a model in which the balance between self-renewal and differentiation is regulated by competitive occupancy of the proximal promoters of key stem cell genes such as *Zfp42/Rex-1*, *Nanog*, *Pou5f1(Oct4)*, *Lefty1 *and *Lefty2*, and others such as the *Klf *genes themselves.

## Results

### Differentiation of ES cells in serum and serum-free^B4L ^culture

**Figure 1:**
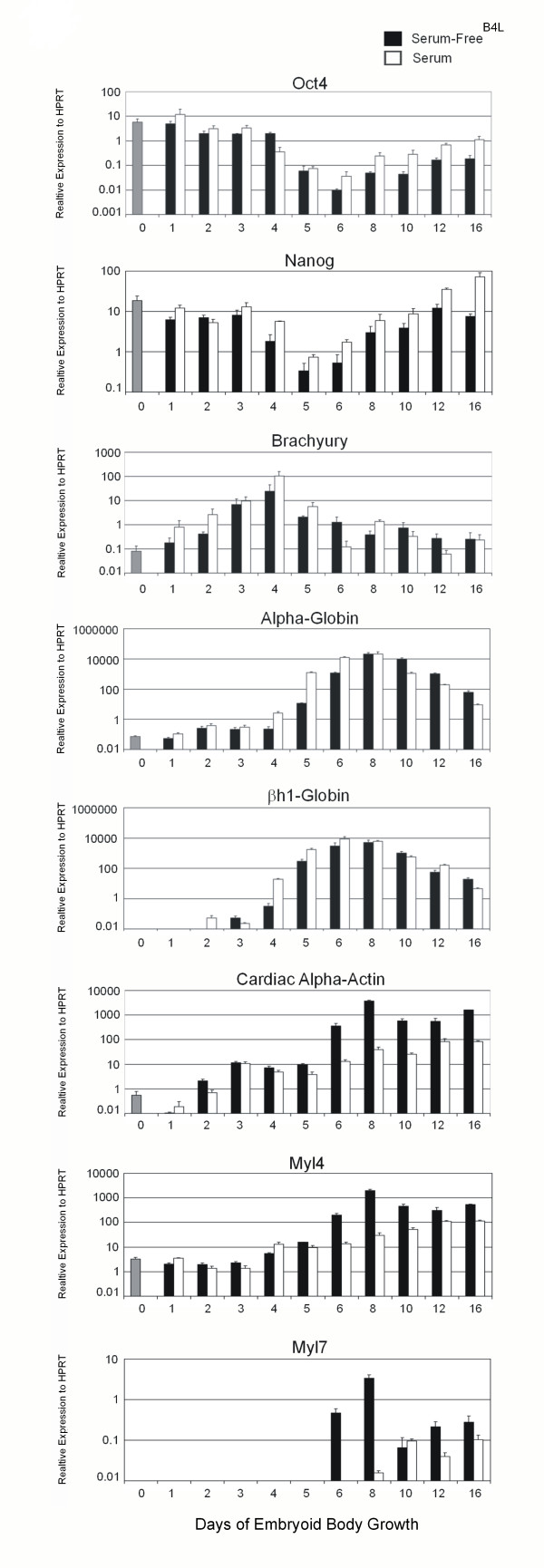
**Dynamic gene expression during EB differentiation in serum and serum-free media containing BMP4 [2 ng/ml]**. Expression of stem cell, primitive streak and late mesoderm genes were analysed by quantitative RT-PCR in undifferentiated ES cells (0, grey bar) and in EBs collected for up to 16 days in serum (white bars) or defined media (black bars) from the same starting ES cell populations. Bars represent the means of three (serum) or two (serum-free^B4L^) biological replicates. The Y-axis represents a log scale normalized relative to the housekeeping gene HPRT. Error bars indicate standard deviation.

The directed differentiation of embryonic stem cells into desirable cell types will depend upon the addition of specific growth factors to defined media and reproducible physical conditions of culture. To establish a baseline for such studies, we undertook a comparison of murine ES cell differentiation using an embryoid body (EB) methylcellulose culture system containing either 10% FBS [2] or a chemically defined BSA-based media containing 2 ng/ml of BMP4, termed serum-free^B4L ^[17]. Triplicate experiments were performed in which ES cells were harvested after feeder depletion, and set up in parallel EB cultures in serum versus serum-free^B4L^. RNA was collected at regular intervals up to 16 days, quantitative RT-PCR was performed on a panel of genes representing defined stages of development to establish the robustness of the culture system (Figure 1), and expression profiling was performed using Illumina^® ^Sentrix Mouse-6 bead arrays (see Methods and [13]).

**Figure 2:**
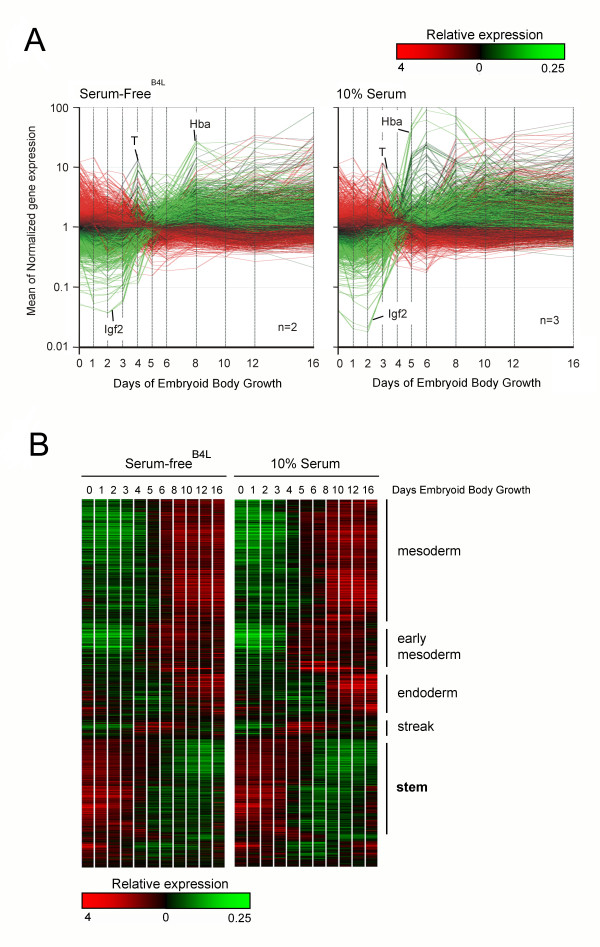
**Gene expression profiles during 16 days of EB differentiation in serum and serum-free media containing BMP4 [2 ng/ml]**. (**A**) GeneSpring representation of the subset of genes from the 48 K Illumina array that showed significant differences in gene expression at 2 or more time points. Each line represents the mean normalised expression of an individual Illumina probe. Colours represent relative expression (red 4× increased; green 0.25× reduced) compared to a gene mean of 1 (black) with expression in undifferentiated ES cells (day 0) as the decision point for colour coding. The expression profiles of T (brachyury), Hba (α-globin), and Igf2 are indicated for reference. (**B**) Clustering (tree) of the genes that showed significant (p < 0.01) changes in gene expression upon differentiation by One-way Welch ANOVA. Expression levels are heat map colour coded (inset) with high expression at Day 0 coloured red and low expression coloured green.

Changes in gene expression were extremely reproducible across ES differentiation experiments with overall correlation of global gene expression for biological replicates in the order or 0.95–0.96. Approximately 30% of the probes were differentially expressed at one or more stages of EB differentiation as determined by one-way Welch ANOVA with multiple testing corrections using the Benjamini and Hochberg False Discovery Rate algorithm (7,967 probes with a p-value of < 0.01 by Welch t-test) (Figure 2A).

To mine for genes with similar patterns of expression across the time course, hierarchical clustering was performed on the 7,967 probes and represented graphically. A large percentage of these (~30%) displayed highest expression in undifferentiated ES cells and subsequent reduced expression over time (labeled **stem **in Figure 2B). A second major cluster showed marked induction after ~day 4–6 of differentiation (labeled **mesoderm **in Figure 2B).

### Putative stem cell genes sets

Previous studies have identified genes important to the stem cell state by comparing expression profiling data from different stem cell populations such as ES cells, haematopoietic stem cells (HSCs) and neural stem cells [6,18]. We used two alternative approaches to identify specific stem cell specific gene expression. Firstly, genes with dynamic expression patterns which correlated closely with *Oct4*, *Sox2 *or *Nanog *expression profiles (Pearson correlation > 0.9) were determined as previously described [6,7]. Second, genes rapidly down regulated ≥3 fold by day 3 of differentiation, a time point where ES cell clonogenicity decreases dramatically, were determined. Not surprisingly there was very little overlap between these gene lists, primarily because *Oct4 *and *Nanog *expression persists during EB differentiation longer than many other stem cell genes (Figure 1). Full lists of genes ranked according to similarity of expression (Pearson correlation >0.9) with expression patterns for *Oct4*, *Nanog *or *Sox2 *are provided in Additional files 1, 2, 3, respectively.

Only fifty-nine genes were down regulated ≥3 fold by day 3 of EB differentiation (Table 1). Many of these have been identified as stem cell markers by other groups using alternative strategies [6,7,19]. Information was compiled on their known expression patterns and/or gene knockout phenotypes via literature searches as referenced in Table 1. Insufficient space exists to discuss every gene and most have been previously identified as ES cell enriched transcripts. Osteopontin is secreted by osteoblasts in the bone marrow niche where it is thought to play an important role in the maintenance of HSC quiescence and maintenance of 'stemness' [20]. It has also been discovered by other groups as a strong marker of ES cells and is regulated directly by the key stem cell transcription factors, Oct4 and Sox2 [21]. Thus, osteopontin is likely to be a useful marker of ES cell pluripotency and may play autocrine or paracrine roles in maintenance of stemness. The second most rapidly down regulated gene was estrogen related receptor β, *Esrrb*, which had previously been identified as a stem cell specific gene and shown to be critical for maintenance of the stem cell state [19]. F-box 15 (*Fbxo15*), a member of the large F-box family of genes [22] was also rapidly down regulated. *Fbxo15 *is known to be highly expressed in ES cells, is regulated by Oct4 and Sox2, but is dispensable for ES cell self-renewal [23]. Recently, the stem cell specificity of this locus was used to screen for cDNAs capable of transforming mouse fibroblasts into ES cell populations [24]. Other notable rapidly down regulated genes included the nodal inhibitors *Lefty-1 *and *Lefty-2*, and *Zfp42*/*Rex-1*, which are also well established as stem cell markers [19].

**Table 1:** Genes rapidly down regulated (>3 fold in 3 days) upon ES cell differentiation

**Gene Name**	**Synonyms**	**Genbank ID**	**Fold ↓ D0 → D3**	**Raw D0**	**Function**	**Refs**
Secreted phosphoprotein 1	Osteopontin; Spp1	NM_009263	26.8	3588	Reg. by Oct4 and Sox2	[21, 96]
Estrogen related receptor, beta	Err2; Estrrb; Nr3b2	NM_011934	24.0	4238	ES cell renewal, placenta	[97]
F-box 15	Fbx15	NM_015798	21.8	4487	Stem cell specific locus	[23]
RIKEN cDNA 2200001I15	2200001I15Rik	NM_183278	18.3	2780		
**Kruppel-like factor 2**	**Klf2; Lklf**	NM_008452	17.0	2639	Vascular defect	[49]
Left-right determination, factor B	Leftb; Stra3; Tgfb4; Lefty 1	NM_010094	14.4	2537	Nodal signalling inhibitor	[87]
Transcription factor CP2-like 1	Crtr-1; LBP-9	NM_023755	13.8	2237	ES cells, repressor	[98]
Zinc finger protein 42	Rex-1; Rex1; Zfp-42	NM_009556	13.4	2108	Nanog-Sox2 induced	[99, 100]
L-threonine dehydrogenase	Tdh	NM_021480	10.9	1764	L-threonine degradation	[101]
DNA (cytosine-5-)-methyltransferase 3-like	D6Ertd14e, Dnmt3l	NM_019448	10.8	5226	Maternal imprinting	[102]
Transcription elongation factor A, 3	S-II, Tcea3	NM_011542	8.6	3874	ICM Expression	[103]
Myosin light chain, skeletal muscle	MLC-2; Mlc2, Mylpf	NM_016754	8.6	1560	Cardiac muscle	[104]
S100 calcium binding protein A6	Cacy, S100a6, calcyclin	NM_011313	8.2	9086	Cell cycle regulation	[105]
Testis expressed gene 19	Tex19, 2410081M02Rik	NM_028602	8.1	1963	Spermatogonia	[106]
Epithelial membrane protein 1	TMP, Emp1	NM_010128	7.9	1030	Neuronal differentiation	[107]
DNA segment, Chr 11, ERATO Doi 636	D11Ertd636e	NM_029794	7.9	1278	Expressed in Morula	[108]
Similar to solute carrier family 7, member 3	Slc7a3	. NM_001004153	7.3	1036		
Left-right determination, factor 2	Lefta; Ebaf; Lefty 2	NM_177099	7.2	1903	Inhibitor of Nodal	[109]
Junction adhesion molecule 2	Jcam2; VE-JAM, Jam2	NM_023844	6.5	713	KO normal	[110]
Lysosomal-associated protein, transmembrane 5	E3, Laptm5	NM_010686	6.5	1539	haematopoiesis	[111]
Discoidin domain receptor 1	Drr1, MCK10, EDDR1	. NM_007584	6.4	5451	Collagen response receptor	[112]
Aminoadipate-semialdehyde synthase	Aass, LKR/SDH; Lorsdh	NM_013930	6.2	1094	Mitochondria	[113]
RIKEN cDNA 2410039E07	2410039E07Rik	NM_212483	6.2	737		
RIKEN cDNA 2410004A20	2410004A20Rik	NM_025890	5.7	644		
Gastrulation brain homeobox 2	Gbx2, MMoxA;Stra7	NM_010262	5.6	1204	Midbrain/hindbrain organiser	[114]
cDNA sequence BC050188	4933403D14	NM_177742	5.6	497		
Cordon-bleu	Cobl, C530045F18Rik	NM_172496	5.1	619		
Similar to EF 1A	eIF-1A, eIF-4C	XM_181357	5.0	538		
Regulatory factor X, 2	Rfx2	NM_009056	4.9	796	ICM expression	[103]
Tissue inhibitor of metalloproteinase 1	Clgi;Timp;TIMP-1	NM_011593	4.6	1732	Metalloproteinase inhibitor matrix	[115]
**Kruppel-like factor 4**	**Klf4, Gklf; **Zie	NM_010637	4.5	597	Epidermal, gut defect	[116]
Insulin-like growth factor binding protein 7	Igfbp7, Fstl2, mac25	NM_008048	4.4	813		
latent transforming growth factor BP4	Ltbp4, 2310046A13Rik	NM_175641	4.4	481		
DnaJ (Hsp40) homolog, subfamily C, 6	Dnajc6, 2810027M23Rik	NM_198412	4.3	490		
**Kruppel-like factor 5**	**Klf5**, Bteb2, IKLF	NM_009769	4.3	1797	Cardiovascular remodelling	[117]
Nuclear receptor subfamily 5, group A, 2	Nr5a2, Ftf;LRH-1	NM_030676	4.2	494	Ovary granulosa cells	[118]
RIKEN cDNA D230005D02	B230207J08	NM_172813	4.2	433		
Nuclear protein 1	Nupr1, Com1;p8	NM_019738	4.1	388	KO normal	[119]
Intercellular adhesion molecule	Icam1, CD54, Icam-1	NM_010493	4.1	716	KO normal, inflammation	[120]
Interleukin-1 receptor-associated kinase 3	Irak3, IRAK-M	NM_028679	3.9	409	Immune homeostasis	[121]
Keratin complex 1, acidic, gene 17	Krt1-17, K17	NM_010663	3.9	368	Embryonic ectoderm	[122]
Stathmin-like 2	Stmn2, Scgn10, Stmb2	NM_025285	3.9	712	KO normal, cell cycle	[123]
Suppressor of cytokine signaling 3	Socs3, CIS3, SSI-3	NM_007707	3.8	1081	LIF singalling	[124]
Lipase, member H	Lpdlr; PLA1B Liph	NM_153404	3.8	446	Lipid metabolism	[125]
RIKEN cDNA 4933405K07	4930427I11Rik	NM_028913	3.8	573		
Erythrocyte protein band 4.1-like 4a	Epb4.1l4, NBL4	NM_013512	3.8	809	beta-catenin/Tcf pathway	[126]
Polo-like kinase 3 (Drosophila) (Plk3)	Cnk, Fnk, PRK	NM_013807	3.7	567	Mitosis regulator	[127]
Heat shock protein 1 (Hspb1)	27 kDa, Hsp25	NM_013560	3.7	3731	Blastocyst expression	[128]
RIKEN cDNA 1700061G19	1700061G19Rik	XM_140052	3.7	425		
Mannosidase, beta A, lysosomal	Bmn, Manba	NM_027288	3.7	484	Glycoprotein cleavage, KO normal	[129]
Developmental pluripotency-associated 3	PCG7, Stella, Dppa3	NM_139218	3.6	450	Expressed in preimplantation embryos	[130]
Lysyl oxidase	TSC-160, Lox	NM_010728	3.6	644	KO cardiovascular defects	[131]
RIKEN cDNA E130014J05	Tet2	NM_145989	3.5	866		
Myeloblastosis oncogene-like 2	B-myb, Mybl2	NM_008652	3.3	718	Cell proliferation G1/S transition	[132]
Pleckstrin homology domain containing, A4	PEPP1, Plekha4	NM_148927	3.3	532	Preimplantation embryo expression	[108]
Aurora kinase C (Aurkc)	AIK3, Stk13	NM_020572	3.3	305	Chromosome segregation	[133]
Fibroblast growth factor binding protein 1	FGF-BP, Fgfbp1	NM_008009	3.1	312	Enhances FGF activity	[134]
Caveolin, caveolae protein 1	Cav1	NM_007616	3.1	438	KO viable, vascular defects	[135]

*Oct4*, *Nanog *and *Sox2 *are not included in Table 1, even though each is required for the maintenance of ES cell pluripotency [25-27] due to their persistent expression during the first 3 days of EB differentiation (Figure 1 and 5A, and Additional file 1). Indeed, *Oct4 *expression is known to persist beyond the inner cell mass stage of development *in vivo*, with transcripts detected in the epiblast and later in the primitive streak, before it becomes further restricted to primordial germ cells [10,28]. 779 genes with a similar pattern of expression to *Oct4 *(Pearson correlation of > 0.9) were identified (Additional file 1). As expected this list also includes many well known stem cell genes such as Sal-like 1, *nodal*, chromobox homolog 7 (*Cbx7*), *Dnmt3b *and Sprouty homolog 4 [19]. We also mined for stem cell genes by looking for similarity (Pearson correlation of > 0.9) with *Nanog *and *Sox2 *gene expression profiles. These provided overlapping but not identical sets of putative stem cell specific genes (Additional files 2, 3). Full data for the 16 days of differentiation can be interrogated and mined for specific differentiation outcomes via Signet (Login_Bruce) (see Methods).

### Tightly co-ordinated waves of gene expression suggest sequential activation of epiblast, primitive streak and mesoderm differentiation programs

**Figure 3:**
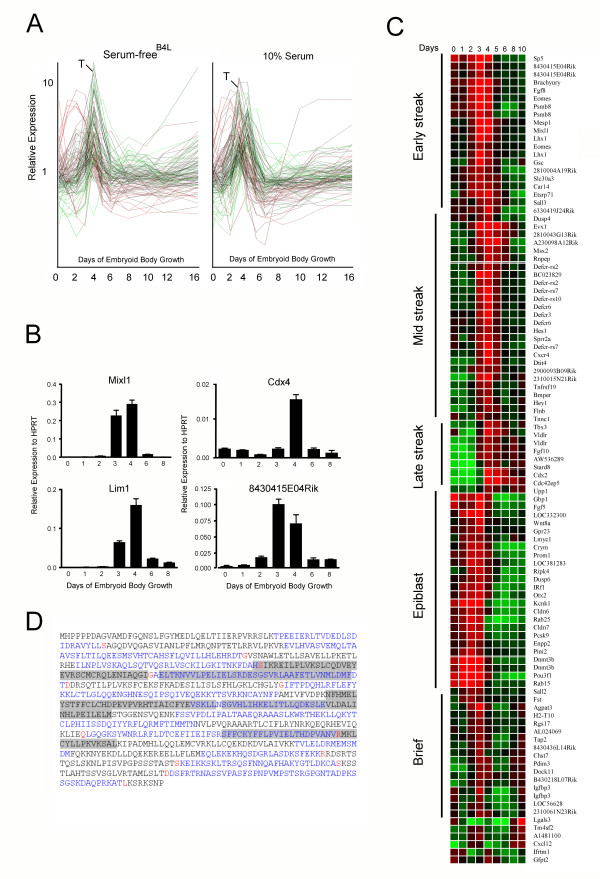
**Mining for genes expressed in patterns consistent with primitive streak, and epiblast differentiation programs**. (**A**) GeneSpring representation of epiblast and streak gene expression profiles during EB differentiation in 10% serum or serum-free^B4L ^culture. The up-regulation of genes between days 2–4 were similar under both conditions. (**B**) qRT-PCR analysis of Mixl, Lim1, Cdx4 and Riken clone 8430415E04Rik. The Y-axis represents expression relative to the housekeeping gene HPRT. Error bars indicate ± SD from three biological replicates. (**C**) 102 genes transiently up regulated during the first 4 days of ES cell differentiation were clustered using a tree algorithm and Pearson correlation of > 0.9. The '**Brief**' group represents gene with very transient expression at day 3. (**D**) Coding region for 8430415E04Rik. The shaded areas represent the three Heat domains.

Many alternative differentiation programs occur simultaneously during ES cell differentiation. Therefore, it is difficult to find gene syn-expression patterns that identify specific programs using standard clustering methods. An alternative approach is to search for expression patterns that closely resemble genes of known function. For example, the T box gene, brachyury, is a well characterized specific marker of the primitive streak [29]. Brachyury expression is transient *in vivo*, a feature recapitulated during ES cell differentiation [2]. Using qRT-PCR, brachyury was induced ~1000 fold from D0 to day 4 and then rapidly silenced to baseline levels by D6 (Figure 1). The dynamics of brachyury expression were similar between serum and serum-free^B4L ^media (Figure 1 and 3A), but serum-free media was unable to support brachyury induction in the absence of BMP-4 (data not shown). Thus, BMP4 (or related factors in serum such as activin) is essential for mesoderm generation. Thirty genes were identified with a similar expression pattern to brachyury (Pearson correlation >0.9) (Table 2) including many genes with established expression or critical functions during primitive streak formation, such as *Mix1 *[30], *Lim1 *[31], *Sp5 *[32] and *eomesodermin *[33]. Transient expression of many of these 'streak-specific' genes was validated by qRT-PCR (Figure 3B and data not shown). In all cases there was a close correlation between the qRT-PCR results and the profiling data (Figure 3A).

**Table 2:** Brachyury-like (Primitive Streak) gene list (Pearson correlation >0.9 with brachyury)

**Description**	**Symbol**	**Corr**.	**Synonyms**	**Genbank ID**	**Expression/Function**
Brachyury (T)	T	1.00	cou;Low;Lr;;T1	NM_009309.1	[33, 136] Primitive streak
Eomesodermin *	Eomes	0.98	Tbr2	NM_010136.1	[33] Primitive streak
Fibroblast growth factor 8	Fgf8	0.97	Aigf;Fgf-8	NM_010205.1	[137] primitive streak expression
Mix1	Mixl1	0.97	Mm1;Mml	NM_013729.2	[30] KO primitive streak abnormal
Lysophospholipase isoform 1	A530050D06Rik	0.97		XM_203596	
LIM homeobox protein 1 *	Lhx1	0.96	Lim1; Lhx1	NM_008498	[31] Primitive streak expression
Mesoderm posterior 1	Mesp1	0.95		NM_008588.1	[138] nascent mesodermal cells
Sp5	Sp5	0.94		NM_022435.2	[32] Primitive streak expression
8430415E04Rik *	8430415E04Rik	0.94	unknown	NM_028980.1	
Axin2	Axin2	0.94	Axi1;Axil;Conductin	NM_015732.3	[139] Wnt signalling inhibitor
Ninein	Nin	0.93	mKIAA1565	NM_008697	[140] Centrosome anchoring
E330009J07Rik	E330009J07Rik	0.93	mKIAA1147	NM_175528	
Proteosome subunit, beta 8 *	Psmb8	0.93	Lmp-7;Lmp7	NM_010724.1	[141] reduced MHC class I
Solute carrier family 30 member 3	Slc30a3	0.93	Znt3	NM_011773	[142] KO no phenotype
DHHC-type Zn-finger containing protein	2400007G07Rik	0.92		AK010296	
Chemokine (C-C motif) ligand 20	Ccl20	0.92		NM_016960	[143] T and B cell regulation
Protease 28 subunit, beta *	Psme2b	0.92	PA28b-p;Psme2-like	NM_011191.1	[144] MHC Class I regulation
D130029J02Rik	D130029J02Rik	0.92		XM_150103	
F-box and leucine-rich repeat protein 14	Fbxl14	0.92	Fbx14l	NM_133940	
WD repeat & FYVE domain containing 1	Wdfy1	0.92	FENS-1;Jr1;WDF1	NM_027057	[145] Endosome and Golgi regulation
ADP-ribosylation factor-like 6 int. protein 2	Arl6ip2	0.92	Aip-2;AV334690	NM_019717.1	
Protease 28 subunit, alpha	Psme1	0.92	PA28a	NM_011189.1	[146] Proteasome activator MHC Class I
TAF10	Taf10	0.92	30 kDa;Taf2h;TAFII30	NM_020024.3	[147] KO ICM phenotype
Helicase with zinc finger domain	Helz	0.91		NM_198298	[148] Primitive streak expression
Transmembrane 4 superfamily member 10	Tm4sf10	0.91	BCMP1	NM_175771.2	
1190004M23Rik	1190004M23Rik	0.90			
Carbonic anhydrase 14	Car14	0.90		NM_011797.1	[149] KO no phenotype
Male enhanced antigen 1	Mea1	0.90	Mea-1	NM_010787.1	[150] Testis development
Delta-like 3, transcript variant 1	Dll3	0.90	pu;pudgy	NM_007866.1	[151] primitive streak expression
Ring finger protein 30	Rnf30	0.90	MURF;MURF-3	NM_021447.1	[152] Myogenic regulator

* More than one independent illumina probe for these genes were identified

Interestingly, four novel transcripts were identified in this list (Table 2). One of these, 8430415E04Rik, was detected by two independent probes, strongly supporting its 'streak-specific' expression; by qRT-PCR there was ~50 fold induction from day 2 to day 3 of differentiation and a decrease to near baseline levels by day 6 (Figure 3B). The gene contains 24 exons, and the full length cDNA encodes for a protein of 868 amino acids (Figure 3D) which is highly conserved in vertebrates (Additional file 4). It has little homology to other proteins apart from three predicted HEAT domains or *armadillo *domains (grey shading in Figure 3D). Drosophila *armadillo *interacts with β-catenin via its HEAT/armadillo repeat [34], which suggests the protein encoded by 8430415E04Rik may also be involved in protein-protein interactions.

The list of primitive streak genes in table 2 is smaller than that previously published [10] due to the very stringent criteria used to determine brachyury-like patterns of expression. Thus, to find further genes characteristic of epiblast, early and late primitive streak programs, probes were identified which were transiently up and down regulated at least two fold with peak expression at day 2, 3 or 4 of EB differentiation. Gene lists were combined (102 Illumina probes) and clustered into epiblast, early, mid and late streak groups plus a unique group in which expression was very transient at day 3 of differentiation (designated 'brief' at the bottom of Figure 3C). Also, lists were generated by searching for similarity of dynamic expression profiles to known genes which are accepted specific markers of specific stages of mouse development. For example, *Fgf5 *is specifically expressed in the epiblast [35], and *Wnt5a *is expressed in the late primitive streak [36]. The *Fgf5*-like (epiblast) list includes the methyltransferase, *Dnmt3b*, which was massively up-regulated in the first 2 days of EB differentiation (Additional file 5) and is known to be essential for mesoderm differentiation [37]. This list also includes *Pim2*, *Zic2*, *Wnt8a*, the SP1 (and possibly SP5) cofactor *Crsp2*, and *Irf1*. The W*nt5a*-like (late primitive streak) list contains 76 genes including many homeobox genes (*Hoxb2*, *Hoxd1*, *Hoxb6 *and *Hox8*/*Msx2*), *Cdx2*, *Hand1*, *Tbx3, Bmp4 *and the VEGF receptor *Flt1 *(Additional file 6). Many are expressed at E10 of mouse development in the remnants of the primitive streak [36], suggesting the *Wnt5a*-like list includes many genes known to pattern the embryo following formation of the germ layers. There is a significant overlap between the genes we identified as being epiblast-, early and late streak-specific and those recently identified using a similar EB differentiation system and Affymetrix arrays [10], and by other groups [2,38]. In addition, there are addition novel RIKEN cDNAs in these lists, which are likely to encode for interesting proteins worthy of further study (Additional files 5, 6).

Following the primitive streak wave of gene expression there was a dramatic activation of many genes (Figure 2A). Many of the most highly induced genes, such as globin genes, heme synthesis enzymes and haematopoietic transcription factors, are known to be expressed in the blood. qRT-PCR for globin genes confirmed > 10,000 fold induction between days 3 and 8 with a steady decline thereafter (Figure 1). Other mesoderm and endoderm programs are also activated at this stage, so it is difficult to mine for these different outcomes.

### Enhanced cardiac gene expression and reduced erythropoiesis in serum-free^B4L ^EB culture

**Figure 4:**
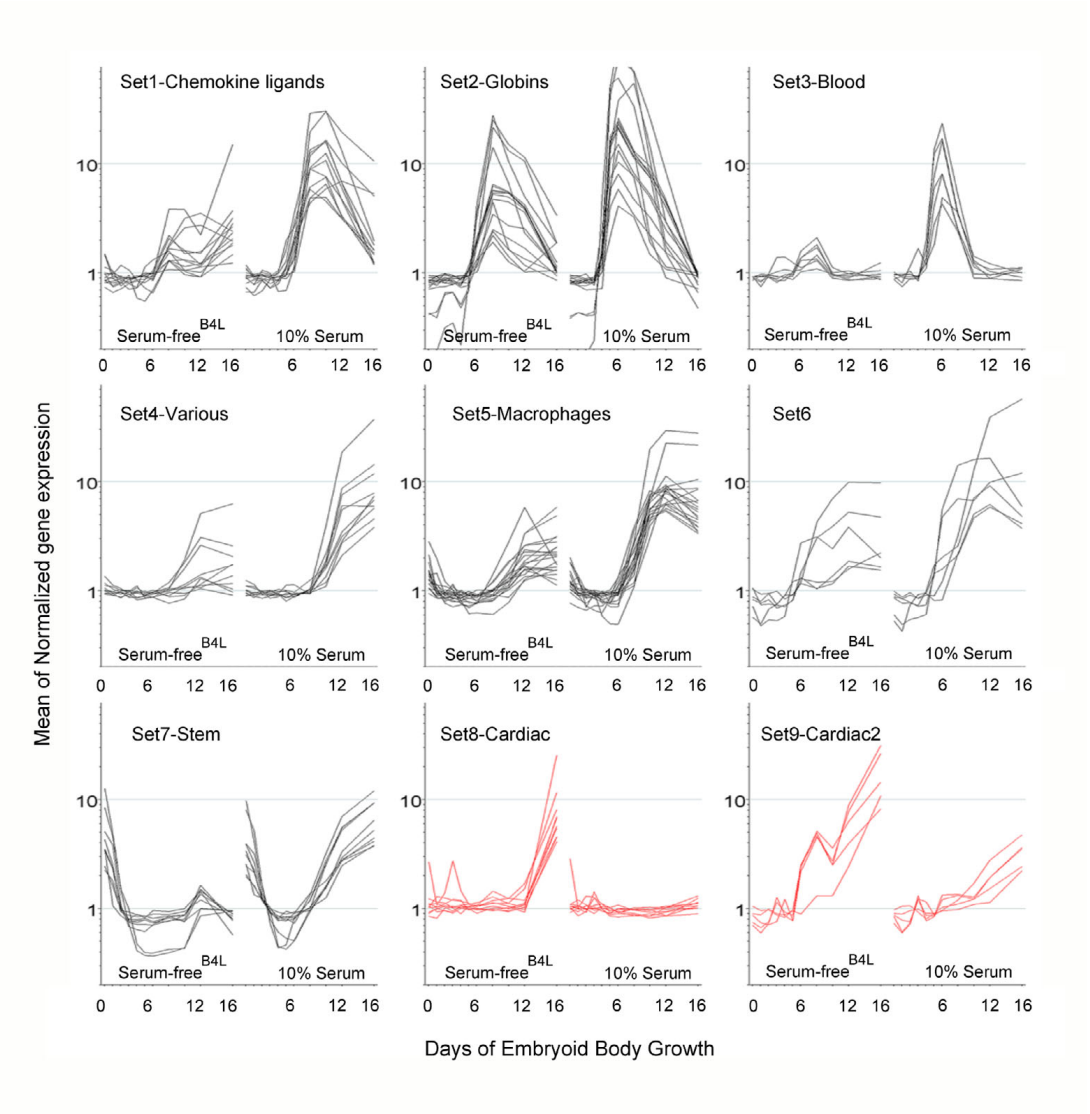
**QT clustering revealed sets of genes differentially expressed between serum and serum-free^B4L ^EB culture**. QT clustering revealed nine sets of genes with similar dynamic expression profiles (Pearson Correlation coefficient of > 0.9). In sets 1–7, expression is higher in serum (black sets). In sets 8–9, expression is greater in serum-free^B4L ^EB culture (red sets). Some sets have been named based on the similarity of overall gene function during differentiation pathways (see Table 3).

Overall there were very strong similarities between ES cell differentiation in serum and serum-free cultures supplemented with 2 ng/ml BMP4. For example, all 29 of the primitive streak gene set, as defined from serum-containing cultures, were also expressed transiently and to a similar level in serum-free^B4L ^media (Figure 3A). Genes differentially expressed at each EB time point (Day 1–16) were pooled and subjected to QT clustering to find sets differentially expressed between serum and serum-free^B4L ^EB culture. Using a minimal cut-off of five genes per group, nine gene sets were identified; seven showed higher expression in serum and two showed higher expression in serum-free^B4L ^(Table 3 and Figure 4).

**Table 3:** Genes differentially expressed during ES cell differentiation in serum versus serum-free defined media (BMP4 2 ng/ml)

**Set 1 – Chemokine ligands (Day 8)**	Symbol	**Synonyms**	**Genbank ID**	**BMP4**	**Serum**
Hydroxyprostaglandin dehydrogenase 15	Hpgd		NM_008278.1	2.22	4.4
Src-like adaptor	Sla	Slap	NM_009192	1.29	.90
Pleckstrin	Plek		NM_019549.1	1.31	4.9
Triggering receptor expressed on myeloid cells-like 1	Treml1		XM_128641.3	1.67	12.6
Glycoprotein 5 (platelet)	Gp5	GPV	NM_008148.2	1.52	8.9
2810484G07Rik				2.07	13.5
Chemokine (C-X-C motif) ligand 7	Cxcl7	b-TG1; CTAP3; LDGF; MDGF;NAP-2	NM_023785.1	1.07	4.8
Angiotensin receptor 1	Agtr1	AT1;AT1a;AT2R1	NM_177322.2	1.58	5.8
Arachidonate 12-lipoxygenase	Alox12	Alox12p;P-12LO	NM_007440.2	1.29	9.2
Chemokine (C-C motif) ligand 4	Ccl4	Act-2; MIP-1B	NM_013652	1.81	19.5
Chemokine (C-X-C motif) ligand 4	Cxcl4	Pf4;Scyb4	NM_019932.1	3.85	29.0
ATPase, Ca^2+ ^transporting, ubiquitous	Atp2a3	Serca3	NM_016745.2	1.30	6.1
Chemokine (C-C motif) ligand 3	Ccl3	Mip1a;Scya3	NM_011337.1	1.75	11.8

**Set 2 – Globins (Day 6)**	**Symbol**	**Synonyms**	**Genbank ID**	**BMP4**	**Serum**

β-major globin	Hbb-b1		AK002258	2.15	21.7
ζ-globin	Hba-x		NM_010405.2	4.30	128.3
ε-y globin	Hbb-y	E-y	NM_008221.2	1.80	38.1
Nuclear factor erythroid-2 (p45)	Nfe2	p45NFE2	NM_008685.2	1.32	10.4
α-globin	Hba-a1	Hba1	NM_008218.1	3.21	89.7
Band-3/anion exchanger 1	Slc4a1	Ae1;CD233;Band 3	NM_011403.1	1.19	26.2
Rik2510042H12			AK011092	1.01	4.1
Aminolevulinic acid synthase 2	Alas2	Alas-2	NM_009653.1	1.14	13.2
β h1-globin	Hbb-bh1	betaH1	NM_008219.2	3.65	61.2

**Set 3 – Blood (Day 8)**	**Symbol**	**Synonyms**	**Genbank ID**	**BMP4**	**Serum**

Alpha hemoglobin stabilising protein	AHSP	AHSP;EDRF	NM_133245.1	1.7	3.4
Myeloperoxidase	Mpo		NM_010824	1.1	3.6
	Slc38a5	SN2; JM24	NM_172479.1	1.3	2.5
Glycophorin A	Gypa	CD235a;GPA	NM_010369.2	2.1	5.2
Cbp/p300-interacting transactivator, with Glu/Asp-rich carboxy-terminal domain, 4	Cited4	Mrg2	NM_019563	1.3	2.5
Mitoferrin	Mscp	Slc25a37	NM_030054.2	1.5	2.4
Kruppel-like factor 1	Klf1	Eklf	NM_010635	1.8	3.6

**Set 4 – Various (Day 16)**	**Symbol**	**Synonyms**	**Genbank ID**	**BMP4**	**Serum**

FXYD domain-containing ion transport regulator 2	Fxyd2	Atp1g1	NM_007503	1.38	6.7
Thioether S-methyltransferase	Temt		NM_009349	0.97	6.0
Mus musculus FXYD domain-containing ion transport regulator 2, transcript variant a	Fxyd2	Atp1g1	NM_052824.1	1.16	5.7
Fibrinogen, alpha	Fga	Fib	NM_010196.1	2.06	11.8
Kininogen-1	Kng1		NM_023125.2	6.25	37.3
Serum amyloid A 3	Saa3	Saa-3	NM_011315	1.04	4.5
Deleted in azoospermia-like	Dazl	Dazla;Tpx-2;Tpx2	NM_010021.2	0.91	3.8
Fibrinogen, alpha	Fga	Fib	NM_010196.1	2.57	14.3
Chitinase 3-like 1	Chi3l1	brp-39;Brp39;Gp39	NM_007695.1	1.75	7.3
Kininogen II			NM_201375.1	1.73	7.9

**Set 5 – Macrophage (Day 16)**	**Symbol**	**Synonyms**	**Genbank ID**	**BMP4**	**Serum**

Neutrophil cytosolic factor 2	Ncf2	Ncf-2; p67phox	NM_010877	2.05	3.56
Complement component 1q beta	C1qb		NM_009777.1	1.13	4.63
Matrix metalloproteinase 12	Mmp12	Mmel	NM_008605.1	3.17	8.53
Complement component 3	C3	ASP;Plp	NM_009778.1	2.17	10.46
CD68 antigen	Cd68	gp110;Scard1	NM_009853	1.84	5.58
Macrophage expressed gene 1	Mpeg1		XM_129176	3.08	6.74
TYRO protein tyrosine kinase binding protein	Tyrobp	DAP12;KARAP;Ly83	NM_011662.2	2.80	4.81
Myosin IF	Myo1f		NM_053214.1	1.64	3.33
Hematopoietic protein 1	Hemp1		NM_153505.2	1.41	4.22
Glutathione S-transferase, alpha 3	Gsta3	Gst2-3	NM_010356.2	1.73	8.67
Glycoprotein (transmembrane) nmb	Gpnmb	Dchil;ipd	NM_053110	1.55	6.63
Pleckstrin homology, Sec7 and coiled/coil domains 4	Pscd4	2510004M07Rik	NM_028195	1.54	3.83
B-cell leukaemia/lymphoma-2 related protein A1b	Bcl2a1b	A1-b	NM_007534	2.49	6.35
Complement component 1q, gamma	C1qg	C1qc	NM_007574.1	1.26	5.27
Fc receptor, IgG, low affinity III	Fcgr3	CD16	NM_010188.2	2.10	5.84
Lysozyme	Lyzs	Lys;Lzm;;Lzp	NM_017372.2	5.80	21.72
Chemokine (C-C motif) ligand 9	Ccl9	CCF18;MRP-2;	NM_011338	2.26	3.85
B-cell leukemia 2 related protein A1d	Bcl2a1d	A1-d	NM_007536	2.51	4.49
EGF-like module containing, mucin-like, hormone receptor-like 1	Emr1	F4/80; Ly71;TM7LN3	NM_010130.1	1.54	3.44

**Set 6 – Day 16**	**Symobol**	**Synonyms**	**Genebank ID**	**BMP4**	**Serum**

Insulin-like growth factor 1	Igf1	C730016P09Rik;Igf-1;Igf-I	NM_010512.2	1.6	4.8
	Prss35		NM_178738.1	4.7	5.9
Protein tyrosine phosphatase, non-receptor type substrate 1	Ptpns1	CD172a; SHP-1;SHPS-1;	XM_149178.1	2.2	3.7
Apolipoprotein A-II	Apoa2	Alp-2;Apoa-2;ApoA-II;Hdl-1	NM_013474.1	9.8	57.4
Spondin 2, extracellular matrix protein	Spon2	;M-spondin;Mindin	NM_133903.2	2.0	12.0

**Set 7 – Stem and late Day 16**	**Symbol**	**Synonyms**	**Genbank ID**	**BMP4**	**Serum**

Fbox 15	Fbxo15			0.96	4.4
Aurora kinase C	Aurkc	AIE1;AIK3;Stk13	NM_020572.1	0.94	4.1
2200001I15Rik			NM_183278.1	0.94	6.4
Hypothetical protein LOC236546		AF067061	NM_199060.1	0.85	3.8
Eukaryotic translation initiation factor 1A	LOC195150		XM_111306.1	0.80	9.3
Similar to Pro-Pol-dUTPase polyprotein; RNaseH	LOC386244		XM_359136.1	0.88	5.2
Similar to hypothetical protein FLJ35105	LOC245109		XM_142517.3	0.89	3.7
Down regulated in renal cell carcinoma	Drr1	TU3A	AK044219	0.57	9.3

**Set 8 – Cardiac Day 16**	**Symbol**	**Synonyms**	**Genbank ID**	**BMP4**	**Serum**

ATPase, Ca++ transporting, cardiac muscle, fast twitch 1	Atp2a1	SERCA1	NM_007504.2	6.9	1.1
Cholinergic receptor, nicotinic, gamma	Chrng	Achr-3;Acrg	NM_009604.2	4.5	1.0
Myosin, light polypeptide 1			BC059087.1	8.0	1.1
Alpha actin, skeletal muscle	Acta1	Acta-2;Acts;Actsk-1	NM_009606.1	25.3	1.2
Tropomyosin 2 beta	Tpm2	Tpm-2;Trop-2	NM_009416.2	11.6	1.3
Cholinergic receptor, nicotinic, alpha	Chrna1	Achr-1;Acra	NM_007389.2	4.1	1.0
Troponin C2, fast	Tnnc2	Tncs	NM_009394.2	5.7	0.9
Neurofilament 3, medium	Nef3	NF-M;NF160;Nfm	NM_008691.1	4.5	1.1
Myosin heavy chain	Myh8		XM_354617	6.7	1.1
Cholinergic receptor, nicotinic, alpha 1	Chrna1	Achr-1;Acra	NM_007389.2	5.4	1.1

**Set 9 – Cardiac 2 Day 6**	**Symbol**	**Synonyms**	**Genbank ID**	**BMP4**	**Serum**

a-Actin, cardiac	Actc1	Actc-1	NM_009608.1	31.2	3.6
Somatostatin	Sst	Smst;SOM;SRIF;SS	NM_009215.1	10.8	2.2
Myosin, light polypeptide 7, regulatory	Myl7	MLC2a;MYL2A;;RLC-A	NM_022879.1	14.3	4.7
Myosin, heavy polypeptide 7, cardiac muscle, beta	Myh7	Myhc-b;Myhcb	NM_080728.1	8.2	2.4
a-Actin, cardiac	Actc1	Actc-1	NM_009608.1	26.3	3.5

The genes in set 9 were expressed more highly in serum-free^B4L ^media from day 6, and genes in set 8 showed up-regulation at day 12 and 16 in the absence of serum (Figure 4). Both sets are comprised almost exclusively of cardiac-specific genes such as myosin heavy chain 7 (*Myh7*), myosin light chain 7 (*Myl7*), the cardiac isoform of α-actin (*Actc1*), troponin (*Tnnc2*), tropomysin (*Tpm2*), the nicotinic cholinergic receptor (*Chrng*), and cardiac muscle fast twitch 1 specific ATPase (*Atp2a1*) (Table 3). Further interrogation of the array data revealed a cohort of cardiac transcription factors, including ISL1, MEF2C, Bop/Smyd1 and Hand2 [39], which were transiently up regulated 1.4 to 3.5 fold in serum-free^B4L ^media compared to serum at day 6 (Signet Login_Bruce). Importantly, this elevated cardiac gene expression program correlated with increased numbers of spontaneously beating EBs in serum-free^B4L ^media (data not shown). Enhanced expression (~100 fold) of *Actc1*, *Myl4 *and *Myl7 *in serum-free^B4L ^media compared with serum-containing media was confirmed by qRT-PCR (Figure 1). Taken together, this data strongly suggests cardiac muscle cell differentiation is either inhibited in serum or enhanced by the addition of BMP4 to serum-free culture.

The genes in set 2 were expressed in both serum and serum-free^B4L ^conditions immediately following primitive streak formation, but expressed to higher levels and slightly earlier in serum (Table 3 and Figure 4). Many of these genes are globins (both embryonic and definitive) suggesting erythropoiesis is activated more robustly in the presence of serum (See Discussion). *Alas2*, a gene encoding the erythroid specific isoform of the first and rate limiting enzyme in the heme biosynthesis pathway, and the erythroid specific transcription factor, p45-NF-E2 are also in set 2 [40]. qRT-PCR for α- and βh1-globin confirmed more rapid expression in serum versus SF^B4L ^media (Figure 1), suggesting factors in addition to BMP4 enhance activation of the blood program from a similar primitive streak platform.

The genes in set 3 were expressed at a similar time in serum but expressed at very low levels or not at all in serum-free^B4L ^media. Again, this set is highly enriched for erythroid specific genes such as alpha hemoglobin stabilising protein (*Ahsp*) [41], Glycophorin A, erythroid Kruppel-like factor (EKLF/*Klf1*) [42] and mitochondrial solute carrier protein (mitoferrin) [43] (Figure 4 and Table 3). Genes in sets 1 and 5 are activated more robustly at late time points in serum cultures. Most of these are specifically expressed in macrophages. Together, these results suggest delayed and less robust primitive haematopoiesis occurs in SF^B4L ^culture compared with serum, an observation confirmed by less robust erythroid cell generation and 'redness' of the EBs from day 6–10 of differentiation (data not shown).

### Rapid changes in Kruppel-like factor gene expression upon ES cell differentiation

Three Kruppel-like factor genes, *Klf2*, *Klf4 *and *Klf5*, were rapidly down regulated during the first few days of EB development (bold type in Table 1). All are members of the KLF family of transcription factors which are characterized by a similar C-terminal domain of three C_2_H_2 _zinc fingers which confers binding to CACC box elements in promoters and more distant regulatory elements [44,45]. *Klf2 *and *Klf4 *have N-terminal transcriptional action domains and act primarily as transcriptional activators, whereas *Klf5 *is best known as a transcriptional repressor [46]. Until recently, *Klf2 *and *Klf4 *were considered to have restricted expression patterns [47,48] with gene knockout animals showing late developmental defects in vasculogenesis and skin differentiation, respectively [49,50]. Recently however, *Klf4 *expression was noted in ES cells [19,51], and forced expression was shown to enhance Oct4 expression and promote self-renewal [51].

**Figure 5:**
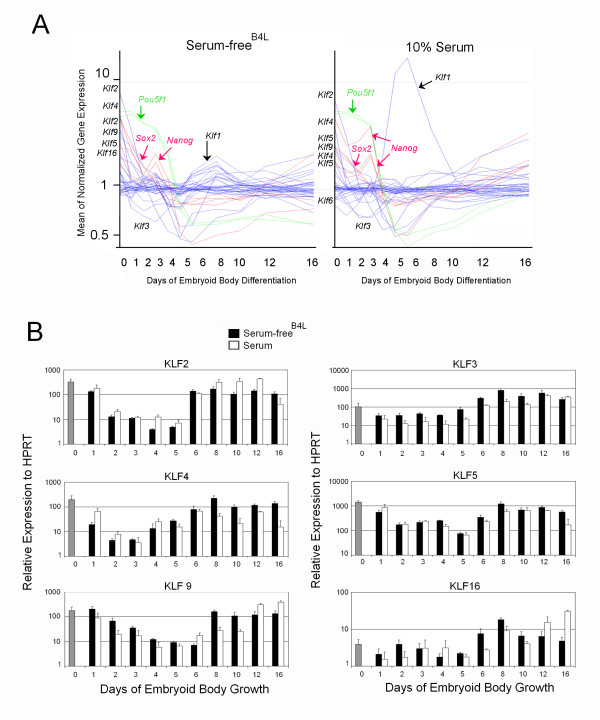
**Kruppel like factors (Klfs) are dynamically expressed during the first few days of ES cell differentiation**. (**A**) GeneSpring plot of normalized gene expression for all KLFs detected during ES cell differentiation in serum or serum-free^B4L ^culture. Most of the genes are listed on the y-axis in order from their highest relative expression in ES cells. There is dramatic up regulation of *Klf1 (Eklf) *only in serum following day 6 of differentiation. Plots representing *Nanog *(red), *Sox2 *(red) and *Pou5f1(Oct4) *(green) are shown for comparison. *Pou5f1 *and *Nanog *gene expression persists at high levels for 2–3 days after *Klf2*, *Klf4 *and *Klf5 *are down regulated. (**B**) Validation of changes in gene expression of six members of the KLF family by quantitative real time RT-PCR. Scheme as described for Figure 2B.

qRT-PCR confirmed the rapid down regulation of *Klf*2, *Klf4 *and *Klf5 *in both serum and serum-free^B4L ^differentiation conditions, although *Klf5 *expression was down regulated more slowly (Figure 5A and 5B, and Table 4). *Klf2*, *Klf4 *and *Klf5 *were all re-induced from day 3–5 of differentiation as mesoderm and endoderm developmental pathways are activated (Figure 5A and 5B). Sixteen of the 17 known KLFs were examined in detail throughout the entire differentiation time course (Table 4). *Klf9 *was also steadily down regulated (>10 fold) between ES cells and day 5 EBs in serum and serum-free^B4L ^media (Figure 5B). *Klf3*, a well characterized transcriptional repressor [52] via its ability to recruit the co-repressor, CtBP [53], displayed an interesting biphasic expression pattern with initial rapid down regulation then re-induction from days 5–6 (Figure 5B). In contrast, *Klf16 *displayed little change in expression levels over the 16 days of EB maturation (Figure 5B).

**Table 4:** Kruppel-like factor gene expression during ES cell differentiation

**Symbol**	**Synonyms**	**Genbank ID**	**Illumina^® ^Probe ID**	**Absolute Expression Level**				
				
				**ES Cell**	**EB (Day)**			
					
					**Serum-free**		**10% Serum**	
				
				**Day 0**	**3**	**6**	**3**	**6**
Klf1	Eklf	NM_010635	scl33570.4.1_25-S	110	105	125	114	**2138**
Klf2^a^	Lklf	NM_008452	scl016598.3_26-S	**2275**	269	157	272	248
Klf3^b^	Bklf;Tef-2	AK046659 NM_008453	ri|B430304G02| 3393-S	112	92	80	102	99
			scl0016599.2_295-S	894	406	733	374	881
			scl27779.8_253-S	638	182	422	251	442
Klf4^a^	Gklf; Zie	NM_010637	scl0016600.2_155-S	149	85	86	104	102
			scl0016600.2_249-S	**575**	89	109	112	127
			scl016600.7_29-S	101	69	64	86	83
Klf5^a^	Bteb2;IKLF	NM_009769	scl0012224.2_233-S	**1981**	699	232	699	274
			scl45215.1.1_294-S	79	91	83	112	105
			scl46026.3_338-S	183	91	78	112	99
Klf6	BCD1;CPBP;Klf6;Zf9	NM_011803	scl023849.4_8-S	1107	1697	1540	1996	1457
			scl45096.3_115-S	177	251	221	307	232
Klf7	9830124P08Rik	AK086122; NM_033563	ri|D930007C01| 2481-S	319	243	207	270	227
			scl0093691.2_69-S	334	281	202	345	231
			scl16685.5_486-S	439	398	305	440	309
Klf8	Klf8	NM_173780	scl0245671.1_6-S	136	113	116	139	120
Klf9^a^	BTEB-1;Klf9	NM_010638	scl53274.2_128-S	**2035**	910	205	1120	394
Klf10	Tieg1; Egra; Tieg	NM_013692 AK043433	scl47275.9_20-S	84	83	70	96	92
			ri|A730095A08| 2144-S	71	65	64	93	81
Klf11	Tieg3; Tieg2b; FKLF	NM_178357	scl0194655.4_70-S	71	68	67	86	78
Klf12	AP-2rep;B130052C06Rik	NM_010636	scl000297.1_3-S	75	70	70	104	93
			scl0016597.1_0-S	79	81	70	104	93
			scl016597.1_75-S	92	102	86	122	121
			scl45206.9.1_30-S	79	81	85	101	92
			scl45212.1.365_19-S	62	61	56	75	73
Klf13	Bteb3;FKLF2; RFLAT-1	NM_021366	scl31250.6.4_21-S	291	284	245	332	308
Klf14	1110025J03Rik;Epfn;Klf14	NM_031183	scl0083395.1_307-S	109	141	103	168	140
			scl13960.1.1_83-S	167	193	166	237	183
			scl13961.1.1_105-S	83	75	73	104	87
Klf15	1810013I09Rik;CKLF;KKLF	NM_023184	scl29751.3_94-S	118	125	101	156	120
Klf16^a^	BTEB4;DRRF	NM_078477	scl37728.2_209-S	**355**	181	177	249	167

a. Enriched in undifferentiated ES cells

b. Biphasic expression pattern (See Figure 5)

**Figure 6:**
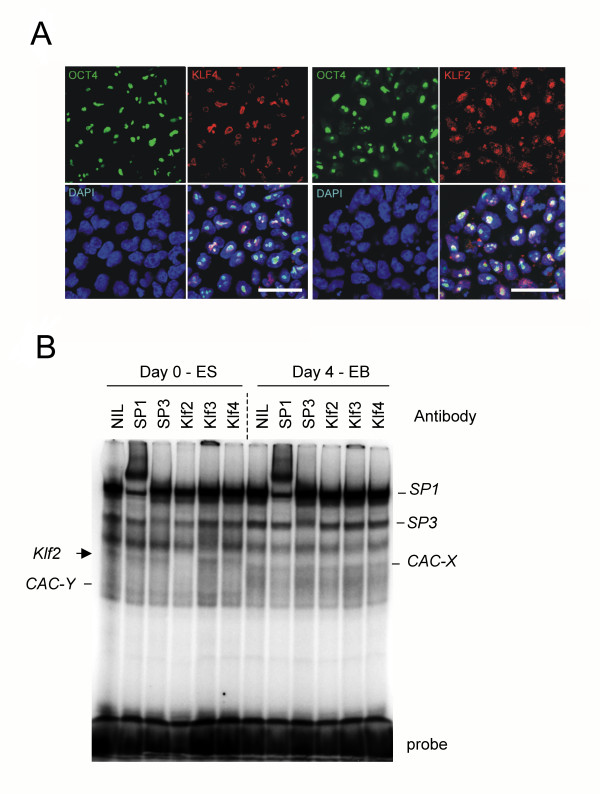
**Kruppel like factor expression and DNA binding activity during ES cell differentiation**. (**A**) Co-expression of Oct4 with KLF2 or KLF4 in ES cells. Indirect immuno-fluorescence shows co-localization of KLF2 and KLF4 with Oct4 in sub-nuclear compartments (possibly nucleoli). Individual confocal images for OCT4, KLF2, KLF4, and DAPI are shown with the corresponding composite image. Scale bar 40 μm. (**B**) Electro-mobility gel shift assay showing changes in DNA binding activities at a conserved CACC box site in the p18-INK4c gene promoter. Nuclear extracts were generated from ES cells or EBs differentiated for four days in serum. Super-shifts were performed with specific antisera for SP1, SP3, KLF2, KLF3, and KLF4 (See Methods). There is strong binding of endogenous Sp1 to the CACC element in ES cells and EB cells. KLF2 DNA-binding activity is present in ES cells as determined by a specific inhibition of binding of the indicated DNA complex with a KLF2 antibody. This activity is lost upon differentiation into EBs. The identity of the CACC box binding activity in EBs denoted CAC-X, and the binding activity in ES cells denoted CAC-Y, was not definitively identified using this panel of antibodies.

*Klf2 *and *Klf4 *co-localize with *OCT4 *in the nuclei of ES cells (Figure 6A). Interestingly, all three proteins were found to preferentially reside in a nuclear sub-compartment (possibly nucleoli for transcription factories), suggesting possible co-involvement in a protein complex or network. Also, endogenous KLF2 was detected by specific CACC box DNA-binding activity in undifferentiated ES cells, which was lost upon EB differentiation (Figure 6B). In contrast, endogenous ES cell KLF4 DNA binding activity was not detected using the p18INK4c promoter CACC box sequence, although recombinant KLF4 was shown to bind to this probe (data not shown). SP1 and SP3 are ubiquitously expressed CACC-box binding proteins, which are dominant in ES cell nuclear extracts (Figure 6B). We hypothesize KLF2, KLF3, KLF4, KLF5 and KLF6 compete with SP1 family members for DNA binding to key *cis *elements in various stem cell genes to regulate expression in positive and negative fashions (see Discussion).

### Enrichment of KLF-binding cis elements in the proximal promoters of stem cell specific genes

To investigate whether Kruppel-like factors might directly regulate stem cell gene expression, and therefore play a role in the maintenance or loss of 'stemness', the presence of KLF transcription factor binding sites (TFBS) within the proximal promoters of all genes in Table 1 was determined. Although a position weighted matrix (PWM) for KLF4 binding site specificity has been published [54], this site does not resemble the CACC box sequences known to bind other KLFs. Since KLF4 has identical DNA-binding residues in each of its three C-terminal C_2_H_2 _zinc fingers to other members of the Kruppel-like factor family [45], it should bind to similar sequences. Based on the crystal structure of the zinc fingers of Zif268 and SP1 bound to DNA [55,56], and testing of specific binding of CACC box variants to recombinant KLF1, KLF3 and KLF17 [45,52,57,58], a generic KLF TFBS has been proposed. Although a SELEX experiment for KLF1 binding has not been published, experiments from our group suggest a C at position 1, an A or C at position 3, an A at position 5, and an A, T or C at position 9 of the 9 bp consensus provides enhanced affinity for KLF1 binding. Taken together, these studies have enabled the development of a PWM (called here KLF-A) that should predict KLF4, KLF2 and other KLF TFBSs (see Methods and Additional file 7).

**Figure 7:**
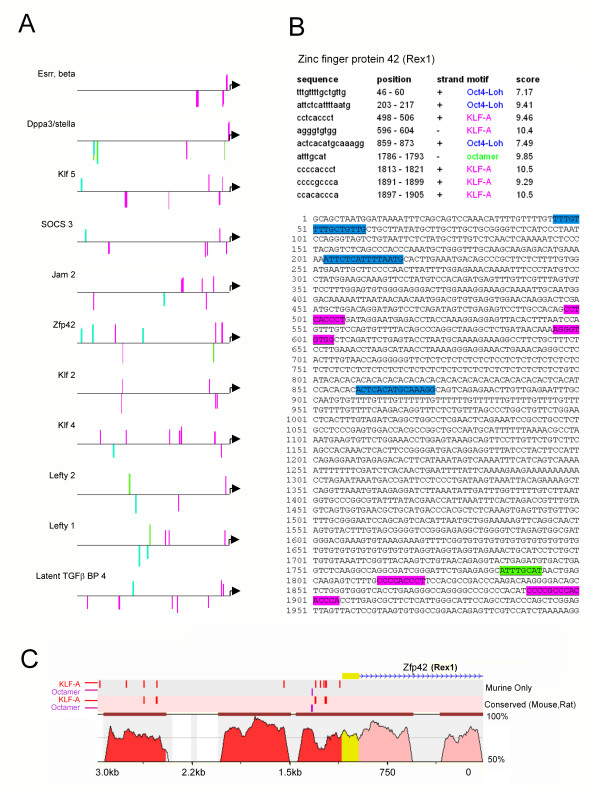
**KLF and octamer binding sites are highly enriched in stem cell gene promoters**. (**A**) A Clover analysis was used to identify over-represented transcription factor binding sites within the promoter sequences of all stem cell genes identified in Table 1. Representative gene promoters are shown, indicating KLF-A binding sites (pink), octamer sites (ATGCWAAT) (green) and extended Oct4 binding sites (Oct4-Loh)(cyan) [60]. (**B**) Clover output for 2 kb of promoter sequence of murine *Zfp42*/*Rex1*. The positions and sequences corresponding to PWMs for KLF-A, octamer and Oct4 occupancy sites are indicated in the table and colour coded in the sequence. The positions are relative to the transcriptional start site. (**C**) ECR Browser output of conserved sequence identity between mouse and rat in the promoter and part of the first intron of the *Zfp42 *gene. The blue arrows indicate the direction of transcription. Yellow indicates the extent of the first (non-coding) exon, pink indicates regions of sequence conservation in the first intron and red indicates regions of sequence conservation in the 5' upstream region. rVISTA was used to find all of the KLF-A and octamer sites in the murine gene (Murine only) and conserved sites between mouse and rat (Conserved mouse, rat).

Clover [59] was used to determine if defined TFBSs were statistically over-represented in the stem cell lists. We searched for PWMs for KLF-A, KLF4 [54], octamer, extended octamer, nanog, and Oct-Sox binary sites as well as negative control sites (gata-1 and E-box) in sequences 2 kb upstream of the TSSs of stem cell genes (Table 1), versus 2 kb of sequences upstream of the entire murine transcriptome (See Additional file 7 for details of PWMs). There was significant over-representation of KLF-A TFBS within the first 2 kb of promoter sequences from the stem list gene list (p value of < 0.01) (Figure 7A), as well as over-representation of octamer sites and extended Oct4-binding sites as defined from ChIP-PET data [60]. No statistically significant over-representation of KLF4 [54], NANOG, GATA1 or E-box TFBSs were identified. All genes in the rapidly down regulated gene list have at least one KLF-A type CACC site, or octamer or Oct4 [60] TFBS. A full list of the promoters with highlighted sites is available on request. Together, this data strongly suggests direct transcriptional regulation of many stem cell genes by KLFs.

The *Zfp42*/*Rex1 *gene promoter is presented as an example of output from the Clover program (Figure 7B). It contains five KLF-A, one octamer and three OCT4 TFBS. The two CACC box elements within the first 250 base pairs of the TSS represent classical extended KLF-binding sites [42,61]. These regions are the same as previously reported [62] although their functional importance has not been determined. Interestingly, two of the three KLF-A TFBS were evolutionarily conserved between the mouse and rat gene promoters (Figure 7C). Also, two further conserved CACC sites at -1.4 and -1.5 kb were identified within 500 bp of extended evolutionary conservation (>70% identity) (Figure 7C). This might act as a KLF dependent enhancer. Most of the stem cell genes in list 1 have conserved CACC box elements in their promoters [63-66]. In some cases these have been reported to bind the ubiquitous SP1, but our Clover analysis suggests they are also likely to bind KLFs (see Discussion).

## Discussion

Expression profiling of murine ES cell differentiation was undertaken over a 16-day time course. We compared gene expression in methylcellulose cultures containing serum versus chemically defined media containing LIF (1 U/ml) and low concentrations of BMP4 (2 ng/ml) [67]. The Illumina^® ^Sentrix Mouse 6 bead array provided a sensitive and detailed platform for analysis of dynamic gene expression. Using various data mining approaches, lists of stem cell-enriched genes and genes that are induced during the *in vitro *equivalent of epiblast and primitive streak stages of differentiation were generated. In combination with other ES cell profiling studies [7], our detailed expression data provides a useful resource for future reverse genetic approaches (i.e. siRNA knockdown) to study the function of these genes during ES cell differentiation and *in vivo *development. We also found a number of previously uncharacterized cDNAs (RIKEN clones) which could play important roles during development.

Importantly, the loss of pluripotency, measured by Oct4 gene expression, was comparable between serum and serum-free^B4L ^EB culture, following a predicted decrease over the first 6 days. Surprisingly, Oct4 gene expression gradually increased following day 6, an observation independent of the cell lines used (data not shown). It remains undetermined if expansion of Oct4 positive ES cells persists as undifferentiated populations within our EB culture system. Previous studies have identified the development of Oct4 positive primordial germ cells (PGC) following 12 days of ES cell differentiation [68-70], suggesting the Oct4 profile may alternatively represent the expansion of non-ES cell populations. Although the expansion of some undifferentiated ES cells is possible, the array profile does not suggest global persistence or up-regulation of ES gene expression late in the EB program. Also, markers of mesoderm induction such as brachyury and Mixl, do not show persistent up-regulation following peak expression at day 4, suggesting EBs are unlikely to harbour cells which are delayed or arrested from entering the differentiation program.

We found significant differences in cardiac gene expression during EB differentiation in serum and serum-free^B4L ^culture. Our current understanding of cardiac development provides possible insights into this observation. Briefly, *in vivo *studies have revealed BMPs secreted from the anterior lateral plate, and ill-defined signals from anterior primitive endoderm, are key inducers of cardiac development [71-73]. The administration of recombinant BMP2 or BMP4 to chick explant cultures induces cardiac differentiation in non-cardiogenic mesoderm [71] and *Bmp2 *knockout animals develop cardiac abnormalities [74]. Conversely, Wnts secreted by the neural tube are strong suppressors of cardiogenesis. Together, these opposing signals act to establish the borders of the heart field [71,75]. By mimicking the environment that establishes cardiogenesis *in vivo*, assays capable of directing cardiomyocyte production from ES cells have been established. BMPs can efficiently enhance the cardiac program when added to EB culture [76,77], whereas BMP inhibition drastically suppresses this outcome. In our hands cultures supplemented with BMP4 (2 ng/ml) supported cardiomyocyte maturation (as determined by expression of cardiac specific genes) with greatest expression detected at Day 8–10. This correlated with increased numbers of spontaneously beating EBs, and the timing of initial spontaneous contractions during murine embryogenesis [78]. The cardiac program was significantly reduced in culture containing 10% serum, an observation supported by a number of other studies [79,80]. This suggests the constituents of serum are inhibitory to cardiomyocyte development. Although a detailed assessment of cardiomyocyte differentiation was not the focus of this analysis, the array output, qRT-PCR profiles and morphological observations described supports the usefulness of this assay in future investigations. In addition, the defined constituents of serum-free^B4L ^media provides an excellent opportunity to identify additional recombinant factors required to further expand cardiac progenitor cell production from EBs.

In contrast, the hematopoietic program was more pronounced within EBs grown in 10% serum than BMP4 alone (Figure 1 and 4). In particular, EKLF (*Klf1*) expression was significantly reduced in serum-free media supplemented with BMP4 (2 ng/ml) compared with serum (Figure 5A and Table 4). EKLF is essential for regulation of a large cohort of erythroid specific genes [81,82], many of which were identified in sets 2 and 3 (Figure 4). Thus, it is likely that reduced EKLF expression in SF^B4L ^media directly results in inhibition of a cascade of erythroid gene expression. Previous work showed the addition of BMP (at 5 ng/ml) to serum-free ES cell culture induces EKLF expression and restores hematopoietic cell differentiation [83]. Although a weak hematopoietic program was observed in SF^B4L ^culture, the concentration of BMP4 used was less than that used by Adelman et al. Thus, we suggest a robust cardiac (anterior-ventral mesoderm) gene expression program is induced by low conentrations of BMP4 (or other BMPs), whereas a robust blood gene expression program (posterior-ventral mesoderm) requires >2 ng/ml of BMP4 or additional growth factors. Recently, mesoderm derived progenitor cell populations for hematopoietic and cardiac lineages were studied during EB development using ES cells in which GFP is targeted to the brachyury locus [80]. Within day 3.25 EBs, GFP^+^Flk1^+ ^cells were shown to represent hematopoietic precursors, whereas GFP^+^Flk1^- ^cells were significantly enriched for cardiac progenitor populations. It would be interesting to determine if the ratio of Flk1^+^/Flk1^- ^within the brachyury positive population is altered between our two culture conditions.

A number of the Kruppel-like factor family of transcription factors were dynamically expressed in the first few days of ES cell differentiation. It was initially surprising to find highly enriched *Klf2 *and *Klf4 *expression in ES cells, since both are considered markers of terminally differentiated cell types such as skin [50], gut [84], vascular smooth muscle [49,85] and lymphocytes [86]. However, recent evidence suggests KLFs may regulate stem cell function, since *Klf4 *is enriched in ES cells [19] and forced over-expression within these cells can maintain pluripotency in the absence of LIF [51]. Furthermore, *Klf4 *can bind the Lefty1 gene core promoter co-operatively with *Oct4 *and *Sox2 *[65]. Although Lefty 1 and 2 are best characterized as repressors of nodal, acting to regulate left-right patterning [87], our results and those of others, suggest a possible redundant role for lefty proteins during the maintenance of ES cell pluripotency [65]. Similarly to the Oct4 expression profile, many of the KLF family members also increased in expression late in the differentiation program. As mentioned, the KLFs are expressed in diverse tissues during development. It is therefore expected that the profiles obtained reflect the generation and early specification of mesoderm and endoderm cells following primitive streak gene activation at day 4.

Based on the likely identical binding specificity of the KLF family, established transcriptional activation and repression roles of certain family members [44], and bioinformatic evidence of a high prevalence of KLF TFBSs in many stem cell genes, we propose KLF competition for occupancy of these CACC box elements might determine self-renewal versus differentiation of ES cells. According to this model, high levels of KLF2 and KLF4 expression in undifferentiated ES cells would lead to occupancy of CACC box elements in promoters of stem cell genes such as *Pou5f1/Oct4*, *Nanog, Esrrb, Zfp42/Rex1, Lefty1 *and *Lefty2*. Furthermore, the *Klf2 *and *Klf4 *genes themselves have CACC box elements in their proximal promoters (Figure 7A), suggesting a positive feedback loop within ES cells is likely. Interestingly, gene knockout and lentiviral shRNA gene knockdown of *Klf2 *or *Klf4*, does not lead to an obvious stem cell defect [19,49,50,85], suggesting these two KLFs may have functionally redundant roles during the maintenance of pluripotency. Future analysis of KLF2/KLF4 double knockout ES lines or the knockdown of both proteins using RNAi technologies will be necessary to validate this hypothesis. Upon ES cell differentiation, down regulation of *Klf2 *and *Klf4 *was very rapid, whereas down regulation of *Oct4 *and *Nanog *was delayed for one to two days. We suggest loss of KLF2 and KLF4 binding to *Pouf1/Oct4*, *Nanog*, *Esrrb *and perhaps other stem cell promoters, could be directly responsible for their down regulation. Moreover, *Klf5 *was down regulated more slowly, and *Klf3 *was up-regulated in the first two days of differentiation, suggesting these KLFs may function primarily as transcriptional repressors at the *Pouf1*/*Oct4*, *nanog, Zfp42 *and *Esrrb *gene promoters. Once this differentiation driving transcription network is activated we suggest KLF3, KLF5 and KLF9 can accelerate loss of the stem cell state by directly repressing expression of the *Klf2 *and *Klf4 *genes themselves. Therefore, a combination of cross regulation of transcriptional outputs, and competition for occupancy of key stem cell promoters by KLF proteins with differing biochemical properties but identical DNA-binding capacities, may determine self-renewal versus differentiation outcomes. Again validation of this model requires functional analysis. It is important to note we have not proven occupancy of stem cell gene promoters by the KLFs *in vivo*. Chromatin immuno-precipitation (ChIP) experiments in ES cells and differentiated populations are essential to validate this model, however ChiP grade antibodies are not currently available.

This scenario is not necessarily limited to ES cell differentiation. A similar program may take place in many adult stem and progenitor cells populations. Certainly competition between *Klf4 *and *Klf5 *has been suggested to regulate cell growth [88], and *Klf1 *and *Klf3 *compete for binding and have opposing effects in blood cells [89]. Furthermore, an imbalance between occupancy of key stem cell or proliferation gene promoters by KLFs, with differing activating and repressing functions, could be responsible for the development or progression of many common forms of cancer. For example, loss of hetero-zygosity for *Klf4 *and *Klf5 *has been found in colon, stomach, breast, prostate and liver cancers [90,91].

In addition to the proposed complex transcriptional interplay between KLFs and stem cell gene promoters, direct protein-protein interactions and protein networks involving KLFs are possible. We showed sub-nuclear localization of KLF2 and KLF4 with OCT4 in ES cells (Figure 6A), and KLF4 can co-operate with OCT4 and SOX2 to drive expression of the Lefty1 core promoter [65]. Also, very recent biochemical purification of NANOG-interacting proteins discovered KLF4 as part of a NANOG-OCT4 network in ES cells (Stuart Orkin, personal communication). This is also consistent with the ability of KLF4 to co-operate with OCT4, SOX2 and c-MYC to drive de-differentiation of fibroblasts into ES-like cells [24].

## Conclusion

Defining the genetic regulation of ES cell self renewal and differentiation will be instrumental for the development of future cell based therapies. Using 47 K Illumina^® ^Sentrix bead arrays, the differential expression of genes during 16 days of ES cell differentiation was determined. Hierarchical gene clustering and correlation statistical analyses lead to the identification of a small cohort of genes which define the stem cell state. Historically, ES cell differentiation is achieved in culture media containing 10–15% FBS. A direct comparison between ES cell differentiation in serum and a serum-free media was undertaken. Surprisingly, global gene expression profiles were comparable between culture conditions, with the exception of mesoderm derived cardiac and haematopoietic transcripts. These results support the use of defined serum-free approaches for the directed differentiation of ES cell commitment. Expression of many KLF family members were enriched in ES cells and rapidly down regulated upon differentiation. KLF2 and KLF4 were co-localisation with OCT4 in ES cell nuclei and KLF transcription factor binding sites were over-represented within the promoters of the putative ES cell gene list (p < 0.01). Taken together, this data strongly suggests KLF family members regulate the maintenance of ES cell pluripotency.

## Methods

### ES cell culture, embryoid body formation and immunohistochemistry

W9.5 ES cell differentiation was performed as described (Bruce et al., Differentiation, in press). For immuno-fluorescence, feeder depleted W9.5 ES cells were seeded onto sterile gelatin-coated glass cover slips in ES cell media, fixed in 4% PFA for 10 min then washed in PBS. Cover slips were boiled in 10 mM citric acid (pH 6.4) for 10 min, washed in PBS and permeabilized in 0.18% triton X-100 in PBS for 10 mins. For OCT4/KLF4 detection, the cover slips were blocked in 1% BSA then incubated with rabbit polyclonal antibody raised against OCT4 (Abcam #ab19857) and goat Anti-mouse KLF4 (R&D Systems #AF3158) overnight at 4°C, then incubated in goat anti-rabbit Alexa-488 (1:400) (Molecular Probes #A11008) and donkey anti-goat Alexa-647 (1:400) for 1 hour. For co-detection of OCT4/KLF2, the Zenon Rabbit IgG labelling kit was used (Molecular Probes #Z-25308). 1 μg of KLF2 rabbit polyclonal antibody (Santa Cruz SC-28675) was labeled with Alexa647 Fab fragment following the manufacturer's instructions. Cover slips already stained for OCT4 (Alexa488) were incubated with labeled KLF2 for 1 hr then washed and post-fixed in 4% PFA to ensure strong signal detection. DAPI 1:5000 (Molecular Probes #D3571) was used to detect nuclear localization. Cover slips were mounted on SuperFrost^® ^Plus slides (Menzel-Glaser) with VectaShield mounting media (Vector Laboratories H-1000). Confocal microscopy was performed on LSM 510 META Carl Zeiss microscope system.

### RNA extraction, cDNA synthesis and real-time RT-PCR data analysis

Total RNA was made using TRIzol (Invitrogen), and cDNA was synthesized from 2 μg of DNase1-treated total RNA using Superscript III (Invitrogen) and oligo-DT_12–15 _(Promega) according to the manufacturer's instructions. Quantitative RT-PCR (qRT-PCR) was carried out using the Applied Biosystems SYBR-green dye system and 7500 Real Time Cycler in 96-well plates. Cycling variables were as follows: 50°C for 2 minutes, 95°C for 10 minutes, then 40 cycles of 15-second denaturation at 95°C and 1-minute at appropriate annealing temperatures, optimized for each set of primers based on dissociation curves (Additional file 8). Expression levels were normalized to HPRT as determined from the ratio of delta CT values. Mean of relative expression ± SD was determined from three biological replicates. Sequencing of gel-purified amplicons was performed to ensure correct product amplification.

### Micro-array hybridization and analysis

Expression profiling was performed using Sentrix Mouse-6 Expression BeadChip arrays from Illumina^®^. RNA was assessed for integrity using the Agilent Bioanalyzer 2100 and RNA integrity (RIN) scores above 9.5 were present in all samples. Amplification was performed with 500 ng of total RNA using the Illumina TotalPrep RNA Amplification kit (Ambion) with a 12 hour in vitro transcription reaction period. The quantity and quality of biotin-UTP incorporated cRNA was also assessed on the Agilent Bioanalyzer 2100. Amplified cRNA (1500 ng per array) was hybridized to Mouse-6.v1 BeadChip arrays according to the manufacturer guidelines and detected with Fluorolink Streptavidin-Cy3 (Amersham Biosciences). Arrays were scanned using the Illumina BeadStation Scanner. The raw intensity values obtained for the scanned array images were compiled using the proprietary BeadStudio v1.5.1.3 software and imported into GeneSpring GX v7.3.1 (Agilent). A mouse Illumina probe set was defined in the GeneSpring Workgroup using the Illumina targetIDs as the unique identifiers and annotated according to array content files supplied by illumina^®^. Data normalization was performed by first setting all measurements less than 0.01 to 0.01, then applying per chip normalization to the 50^th ^percentile, and per gene normalization to the median.

From an interpretation which included the three FBS experiments, a non-parametric Welch ANOVA (where variances were not assumed equal) was performed on all 46,120 probes to find a subset of genes whose expression varied significantly throughout the differentiation time course. A Benjamini and Hochberg False Discovery Rate multiple testing correction was applied to reduce the number of false positives. This yielded a set of 7,967 probes that showed statistically significant (p < 0.05) differences in expression by Welch t-test. This approach simplified running the data mining algorithms to find syn-expression patterns by removing genes such as housekeeping genes which did not display differential expression, or genes which did not reach a threshold of expression at any time points.

Genome interpretations were generated in GeneSpring GXv7.3.1 (Agilent) in which the mean relative expression level for each gene was represented at each time point relative to the average of all time points. A number of statistical approaches were used to find syn-expression patterns. A Pearson correlation (between 0.9 and 1.0) was performed to find genes with similar expression profiles to *Pouf1/Oct4*, *nanog*, *sox2*, *fgf5*, *wnt5 *and *brachyury*. Hierarchical clustering was performed using the gene by gene and group by group 'tree' algorithm within the GeneSpring (Agilent) program. This compares each probe with every other in the lists to generate similarity statistics and a tree representation of similarity based on a Pearson correlation. Also, quality threshold (QT) clustering [92] was used to define distinct subsets of co-expressed genes. The entire data set for the experiments is available via GEO as well as via our instance of Genespring (contact corresponding author).

### Electro-mobility gel shift assays (EMSA)

Nuclear extracts were made from undifferentiated ES cells and embryoid bodies grown for four days in 10% serum. Electromobility gel shift assays (EMSA) were performed as previously described [61] using double stranded oligonucleotide probes corresponding to an extended CACC site in the p18INK4c promoter (sense strand 5'-gttgggcggggcgtgggcggggcc-3') (Tallack, et al., submitted). Supershifts were performed with specific antibodies raised against SP1 (Santa Cruz SC-059x), SP3 (Santa Cruz SC-644x), KLF2 (Santa Cruz SC-28675), KLF3 [52] and KLF4 (Santa Cruz SC-12538x).

### Bioinformatic searches for CACC sites and other over represented elements in promoters of stem cell genes

The data-mining tool Biomart (accessed via Ensembl release 36 of the mouse genome), was used to collate 2 kb of sequences upstream of the transcriptional start sites (TSSs) of all genes listed in Table 1. The Clover program, run via the MotifViz web interface [59], was used to search for over-representation of position weighted matrices (PWMs) for octamer [93], Oct4 [60], Oct-Sox [60], KLF4 [54], E-box, GATA-1 [94] and CACC (KLF-A) elements in these promoter sets. The PWMs are listed in Additional file 7. The ECR Browser [95] was used to determine phylogenetic conservation of cis elements between mouse, rat and human genomes.

## Abbreviations

BSA, bovine serum albumin; EB, embryoid body; EMSA, electromobility gel shift assay; ES, embryonic stem; FBS, fetal bovine serum; KL, Fkruppel-like factor; LIF, leukemia inhibitory factor; MEF, mouse embryonic fibroblasts; PFA, paraformaldehyde; PWM, position weighted matrix; TFBS, transcription factor binding sites.

## Competing interests

The author(s) declares that there are no competing interests.

## Authors' contributions

SB established the EB differentiation assay, collected and purified RNA samples, designed and carried out the quantitative RT-PCR studies and drafted the manuscript. BG carried out the micro array protocol. LB Carried out experimentation of mesoderm specific gene expression. MG performed the array statistical analysis and annotation. SG participated in the design of the study and performed the statistical analysis. AP conceived the study, and participated in its design and coordination and helped to draft the manuscript. All authors read and approved the final manuscript.

## Supplementary Material

Additional file 1 Oct4 gene list. The data provided lists all genes expressed during 16 days of embryoid body differentiation with similarity to Oct4 (Pearson correlation >0.9). Click here for file

Additional file 2 Nanog gene list. The data provided lists all genes expressed during 16 days of embryoid body differentiation with similarity to Nanog (Pearson correlation >0.9). Click here for file

Additional file 3 Sox2 gene list. The data provided lists all genes expressed during 16 days of embryoid body differentiation with similarity to Sox2 (Pearson correlation >0.9). Click here for file

Additional file 4 Sequence alignment of RIKEN clone 8430415E04RIK. The data provides an alignment of RIKEN clone 8430415E04RIK using human, mouse, chick and zebrafish sequences. Click here for file

Additional file 5 FGF5 gene list. The data provided lists all genes expressed during 16 days of embryoid body differentiation with similarity to FGF5 (Pearson correlation >0.9). Click here for file

Additional file 6 Wnt5a gene list. The data provided lists all genes expressed during 16 days of embryoid body differentiation with similarity to Wnt5 (Pearson correlation >0.9). Click here for file

Additional file 7 Position Weighted Matrices. Provides published data on position weighted matrices for Oct, Sox, KLF-A, KLF4, Nanog, E-box, Gata-1 and their variants. Click here for file

Additional file 8 Oligonucleotide sequences used for qRT-PCR analysis. Lists the oligonucleotide sequences and cycling conditions used for the qRT-PCR analysis. Click here for file
