# Supplementary material for: Dynamic transcription programs during ES cell differentiation towards mesoderm in serum versus serum-freeBMP4 culture
Source: BMC Genomics. 2007 Oct 10;8:365. doi: 10.1186/1471-2164-8-365 (PMC2204012; doi:10.1186/1471-2164-8-365)
Supplement: Additional file 1 — Oct4 gene list. The data provided lists all genes expressed during 16 days of embryoid body differentiation with similarity to Oct4 (Pearson correlation >0.9). [file 1471-2164-8-365-S1.doc]

**Additional file 1:** Oct4 gene list (Pearson correlation >0.9)

| **Description** | **Symbol** | **Corr.** | Synonyms | **Genbank ID** |
| --- | --- | --- | --- | --- |
| POU domain, class 5, transcription factor 1 | Pou5f1 | 1.000 | Oct-3;Oct-3/4;Oct-4;Oct3;Otf-4;Otf3;Otf4 | NM_013633 |
| ets variant gene 5 | Etv5 | 0.996 | 1110005E01Rik;8430401F14Rik;ERM | NM_023794 |
| cDNA sequence BC037006 | BC037006 | 0.994 |  | XM_109956 |
| RIKEN cDNA 2310050B20 | 2310050B20Rik | 0.990 | 9430024O13Rik | NM_025537 |
| RIKEN cDNA 1110006G06 | 1110006G06Rik | 0.988 | B230110F21Rik | NM_028661 |
| Peroxisomal membrane protein 2 | Pxmp2 | 0.988 | 22kDa;PMP22 | NM_008993 |
| RIKEN cDNA A030007L17 | A030007L17Rik | 0.987 |  | NM_026637 |
| Lemur tyrosine kinase 2 | Lmtk2 | 0.986 |  | XM_132499 |
| Sal-like 1 (Drosophila) | Sall1 | 0.985 | Msal-3 | NM_021390 |
| Absent in melanoma 1-like | Aim1l | 0.984 |  | XM_131754 |
| Mitochondrial ribosomal protein L16 | Mrpl16 | 0.984 | 2310039D06Rik | NM_025606 |
| RIKEN cDNA 2900006F19 | 2900006F19Rik | 0.984 |  | XM_355346 |
| Coilin | Coil | 0.983 | Cln80;MGC37472;p80-coilin | NM_016706 |
| Transformation related protein 73 | Trp73 | 0.983 | p73 | NM_011642 |
| RIKEN cDNA A030007L17 | A030007L17Rik | 0.983 |  | NM_026637 |
| Similar to hypothetical protein FLJ10884 | LOC381591 | 0.982 |  | XM_355549 |
| Thyrotropin releasing hormone | Trh | 0.982 |  | NM_009426 |
| RIKEN cDNA E430003D02 | E430003D02Rik | 0.982 |  | AK088068 |
| Spermine synthase | Sms | 0.981 | Gy;gyro | NM_009214 |
| Glutamate oxaloacetate transaminase 1, soluble | Got1 | 0.981 | cAspAT;Got-1 | NM_010324 |
| RIKEN cDNA 1700023F06 | 1700023F06Rik | 0.981 |  | XM_126649 |
| RIKEN cDNA D930048N14 | D930048N14Rik | 0.981 | R75254 | NM_175289 |
| Glycine decarboxylase | Gldc | 0.981 | D030049L12Rik;D19Wsu57e | NM_138595 |
| Mitogen-activated protein kinase-activated protein kinase 3 | Mapkapk3 | 0.979 |  | NM_178907 |
| Nodal | Nodal | 0.979 | Tg.413d | NM_013611 |
| Cyclin E1 | Ccne1 | 0.978 | Ccne;cyclin E | NM_007633 |
| Aminoacylase 1 | Acy1 | 0.978 | 1110014J22Rik;Acy-1 | NM_025371 |
| RIKEN cDNA 1190003J15 | 1190003J15Rik | 0.977 |  | XM_133915 |
| Open reading frame 21 | ORF21 | 0.977 | C21orf18 | NM_145482 |
| RIKEN cDNA D130071O13 | D130071O13Rik | 0.977 |  | AK051750 |
| Importin 8 | Ipo8 | 0.976 |  | XM_132974 |
| BC048355 | BC048355 | 0.976 |  | NM_207161 |
| chromobox homolog 7 | Cbx7 | 0.976 |  | NM_144811 |
| Transmembrane protein 8 (five membrane-spanning domains) | Tmem8 | 0.976 | M83 | NM_021793 |
| Hook homolog 1 (Drosophila) | Hook1 | 0.975 | A930033L17Rik;azh | NM_030014 |
| cDNA sequence BC023741 | BC023741 | 0.975 |  | XM_148582 |
| ES cell-expressed Ras | Eras | 0.975 | Ha-Ras2;HRAS2;HRasp | NM_181548 |
| Cat eye syndrome chromosome region 2 | Cecr2 | 0.975 |  | XM_489839 |
| RIKEN cDNA 5830457O10 | 5830457O10Rik | 0.975 |  | NM_145412 |
| TG interacting factor | Tgif | 0.974 |  | NM_009372 |
| Insulin degrading enzyme | Ide | 0.974 | 1300012G03Rik;4833415K22Rik | NM_031156 |
| RIKEN cDNA 1110008J03 | 1110008J03Rik | 0.973 | AI853657 | NM_029096 |
| CCR4 carbon catabolite repression 4-like (S. cerevisiae) | Ccrn4l | 0.973 | Ccr4;nocturnin | NM_009834 |
| Spire homolog 2 | Spire2 | 0.973 |  | NM_172287 |
| Synaptotagmin 9 | Syt9 | 0.973 | Syt5;Sytv | NM_021889 |
| Breast carcinoma amplified sequence 1 | Bcas1 | 0.973 | 2210416M21Rik;9030223A09Rik;NABC1 | NM_029815 |
| RIKEN cDNA 2610018L09 | 2610018L09Rik | 0.973 | 5730511P15Rik;AV083614 | NM_172254 |
| Akorin, ring finger protein, 1 | Mkrn1 | 0.973 |  | AK005137 |
| RIKEN cDNA 5430425C04 | 5430425C04Rik | 0.972 |  | NM_178381 |
| Tight junction protein 2 | Tjp2 | 0.972 | ZO-2 | NM_011597 |
| RIKEN cDNA 2410012H22 | 2410012H22Rik | 0.972 |  | XM_126343 |
| Coagulation factor II (thrombin) receptor-like 1 | F2rl1 | 0.972 | Gpcr11;PAR-2;Par2 | NM_007974 |
| RIKEN cDNA 2900002G04 | 2900002G04Rik | 0.972 |  | XM_136135 |
| Torsin family 3, member A | Tor3a | 0.972 | Adir | NM_023141 |
| Pyrroline-5-carboxylate reductase family, member 2 | Pycr2 | 0.971 | 1810018M05Rik;Leftb;P5cr2 | NM_133705 |
| Upstream transcription factor 1 | Usf1 | 0.971 |  | NM_009480 |
| Similar to zinc finger protein 206 | LOC332221 | 0.971 |  | XM_285225 |
| Solute carrier family 29 (nucleoside transporters), member 4 | Slc29a4 | 0.971 | ENT4;MGC38048 | NM_146257 |
| Zinc finger protein of the cerebellum 3 | Zic3 | 0.971 | Bn | NM_009575 |
| Testis specific gene A2 | Tsga2 | 0.971 |  | NM_025290 |
| Synapsin I | Syn1 | 0.971 | Syn-1 | NM_013680 |
| DNA segment, Chr 7, Roswell Park 2 complex, expressed | D7Rp2e | 0.971 | D7Rp2;D7Rp2-r;D7Rp2-s;RP2-r;RP2-s | NM_033080 |
| Cadherin EGF LAG seven-pass G-type receptor 3 | Celsr3 | 0.970 | mKIAA0812 | NM_080437 |
| RIKEN cDNA 2810012D02 | 2810012D02Rik | 0.970 |  |  |
| Cytochrome b5 reductase 1 | Nqo3a2 | 0.970 |  | NM_028057 |
| Deoxyuridine triphosphatase | Dutp | 0.970 | 5031412I06Rik;D2Bwg0749e;dUTPase | NM_023595 |
| Synapsin I | Syn1 | 0.970 | Syn-1 | NM_013680 |
| Methyltransferase-like 1 | Mettl1 | 0.970 |  | NM_010792 |
| cDNA sequence BC034099 | BC034099 | 0.969 | A830048M07Rik;MGC31514 | NM_146256 |
| Core-binding factor, alpha subunit 2; translocated to, 2 homolog | Cbfa2t2h | 0.969 | A430091M07;C330013D05Rik;MTGR1 | NM_172860 |
| Solute carrier family 20, member 2 | Slc20a2 | 0.969 | MolPit2;Pit-2;Pit2;Ram-1;Ram1 | NM_011394 |
| Solute carrier family 7, member 7 | Slc7a7 | 0.969 | my+lat1 | NM_011405 |
| RIKEN cDNA A730016F12, transcript variant 1 | A730016F12Rik | 0.969 |  | NM_170673 |
| Phospholipase A2, group IB, pancreas | Pla2g1b | 0.968 | Pla2a;sPLA2IB | NM_011107 |
| FK506 binding protein 5 | Fkbp5 | 0.967 | 51kDa;D17Ertd592e;Dit1;FKBP51 | NM_010220 |
| RIKEN cDNA 2410007B07 | 2410007B07Rik | 0.967 |  |  |
| Apolipoprotein B editing complex 3 | Apobec3 | 0.967 | Arp3;CEM15;MGC7002 | NM_030255 |
| Myc induced nuclear antigen | Mina | 0.967 | 1810047J07Rik;2410057H13Rik;3830408E23Rik | NM_025910 |
| RIKEN cDNA 1700019D03 | 1700019D03Rik | 0.967 | 2410136C24Rik;MGC58779 | NM_144953 |
| B-box and SPRY domain containing | Bspry | 0.967 |  | NM_138653 |
| PR domain containing 4 | Prdm4 | 0.967 |  | XM_125812 |
| Polyhomeotic-like 1 (Drosophila) | Phc1 | 0.967 | Edr;Edr1;Mph1;Rae-28;rae28 | NM_007905 |
| Pleckstrin homology domain containing, family B member 1 | Plekhb1 | 0.966 | evt-1;PHR1;Phret1 | NM_013746 |
| RIKEN cDNA 1700097N02 | 1700097N02Rik | 0.966 |  |  |
| Calsyntenin 1 | Clstn1 | 0.966 | 1810034E21Rik;Cst-1;Cstn1 | NM_023051 |
| Bruno-like 4, RNA binding protein (Drosophila) | Brunol4 | 0.966 | Brul4;BRUNOL-4;C130060B05Rik;CELF4 | NM_133195 |
| Sal-like 1 (Drosophila) | Sall1 | 0.966 | Msal-3 | NM_021390 |
| ets variant gene 1 | Etv1 | 0.966 | ER81;Etsrp81 | NM_007960 |
| RIKEN cDNA 9430038I01 | 9430038I01Rik | 0.966 |  | AK020460 |
| Paladin | Pald | 0.965 | mKIAA1274;MMPAL | NM_013753 |
| DEAD (Asp-Glu-Ala-Asp) box polypeptide 24 | Ddx24 | 0.965 | 1700055J08Rik;2510027P10Rik | NM_020494 |
| WD repeat domain 31 | Wdr31 | 0.965 | 5430402I10Rik;spWD | NM_023597 |
| RIKEN cDNA E430034L04 | E430034L04Rik | 0.965 | G3BP;G3BP2 | NM_011816 |
| Protein phosphatase 5, catalytic subunit | Ppp5c | 0.965 | PP5 | NM_011155 |
| Pogo transposable element with ZNF domain | Pogz | 0.965 | 9530006B08Rik | NM_172683 |
| RIKEN cDNA 9430038I01 | 9430038I01Rik | 0.964 |  | XM_133909 |
| RIKEN cDNA 2210008M09 | 2210008M09Rik | 0.964 |  | NM_194055 |
| Solute carrier family 7, member 3 | Slc7a3 | 0.964 | Atrc3;CAT3;SLC7A1;SLC7A2 | NM_007515 |
| RIKEN cDNA A930026C06 | A930026C06Rik | 0.964 |  |  |
| cDNA sequence BC037006 | BC037006 | 0.964 |  | XM_109956 |
| Eph receptor A2 | Epha2 | 0.964 | Eck;Myk2;Sek-2;Sek2 | NM_010139 |
| HIV-1 Rev binding protein 2 | Hrb2 | 0.964 | 2610511F02Rik;D10Ertd773e | NM_178610 |
| 3-monooxgenase/tryptophan 5-monooxgenase activation protein | Ywhag | 0.964 | D7Bwg1348e | NM_018871 |
| Moloney leukemia virus 10-like 1 (Mov10l1) | Mov10l1 | 0.964 | CHAMP;Csm | NM_031260 |
| squamous cell carcinoma antigen recognized by T-cells 3 | Sart3 | 0.963 |  | NM_016926 |
| Nucleolar protein family 6 (RNA-associated) (Nol6), variant alpha | Nol6 | 0.963 | Nrap | NM_139237 |
| Teratocarcinoma-derived growth factor | Tdgf1 | 0.963 | CR1;cripto | NM_011562 |
| FYVE, RhoGEF and PH domain containing 1 | Fgd1 | 0.963 | ZFYVE3 | NM_008001 |
| CTD (carboxy-terminal domain, RNA polymerase II, polypeptide A) | Ctdp1 | 0.963 | 4930563P03Rik | NM_026295 |
| Cadherin 1 | Cdh1 | 0.963 | E-cadherin;Ecad;Um;UVO;uvomorulin | NM_009864 |
| Solute carrier family 27 (fatty acid transporter), member 2 | Slc27a2 | 0.963 | FATP2;Vlac;Vlacs;VLCS | NM_011978 |
| Expressed sequence AA960558 | AA960558 | 0.963 | C920009D07Rik | NM_133942 |
| Silver | Si | 0.963 | D10H12S53E;D12S53Eh;gp100;gp87;Pmel17 | NM_021882 |
| RIKEN cDNA 2410008K03 | 2410008K03Rik | 0.962 |  | XM_125970 |
| Bcl-2-related ovarian killer protein | Bok | 0.962 | matador;Mtd | NM_016778 |
| ets variant gene 4 (E1A enhancer binding protein, E1AF) | Etv4 | 0.962 | Pea-3;Pea3 | NM_008815 |
| Hosphomannomutase 1 | Pmm1 | 0.961 |  | AK013805 |
| expressed sequence C80638 | C80638 | 0.961 |  | NM_178877 |
| RIKEN cDNA 1810045K06 | 1810045K06Rik | 0.961 |  | XM_144142 |
| WD repeat domain 4 | Wdr4 | 0.961 | D530049K22Rik | NM_021322 |
| RIKEN cDNA 7330423F06 | 7330423F06Rik | 0.961 |  |  |
| RIKEN cDNA 2310047B19 | 2310047B19Rik | 0.961 |  | XM_150151 |
| Envoplakin | Evpl | 0.961 |  | NM_025276 |
| RIKEN cDNA 1110012J17 | 1110012J17Rik | 0.961 | D130071O13;MGC77963;mKIAA0802;t8219b25 | NM_172963 |
| RIKEN cDNA 2210411K11 | 2210411K11Rik | 0.961 |  | XM_355846 |
| Hypothetical Ubiquitin carboxyl-terminal hydrolase family 2 | 9630032J03Rik | 0.961 |  | AK079342 |
| FCH domain only 1 | Fcho1 | 0.960 | 3322402E17Rik | NM_028715 |
| DEAD (Asp-Glu-Ala-Asp) box polypeptide 54 | Ddx54 | 0.960 | 2410015A15Rik;APR-5;DP97 | NM_028041 |
| Solute carrier family 6 (neurotransmitter transporter), member 15 | Slc6a15 | 0.960 |  | NM_175328 |
| B-box and SPRY domain containing | Bspry | 0.960 |  | NM_138653 |
| WD repeat domain 31 | Wdr31 | 0.960 | 5430402I10Rik;spWD | NM_023597 |
| cDNA sequence AK122525 | AK122525 | 0.960 | mKIAA1553 | NM_199028 |
| RIKEN cDNA C130034I18 | C130034I18Rik | 0.960 |  | NM_177233 |
| Paraneoplastic antigen MA2 | Pnma2 | 0.959 | A830049P17Rik | NM_175498 |
| CCR4 carbon catabolite repression 4-like (S. cerevisiae) | Ccrn4l | 0.959 | Ccr4;nocturnin | NM_009834 |
| Polr3a | Polr3a | 0.959 |  | NM_183157 |
| RIKEN cDNA 2810046L04 | 2810046L04Rik | 0.959 | 9330161F11 | NM_173382 |
| Phosphoribosyl pyrophosphate synthetase-associated protein 2 | Prpsap2 | 0.958 | A230054F23Rik;MGC36957 | NM_144806 |
| TAR (HIV) RNA binding protein 2 | Tarbp2 | 0.958 | Prbp | NM_009319 |
| translocase of outer mitochondrial membrane 34 | Tomm34 | 0.958 | 2610100K07Rik;TOM34 | NM_025996 |
| Disabled homolog 1 (Drosophila) | Dab1 | 0.958 | scm;scr;scrambler;yot | NM_010014 |
| RIKEN cDNA 1110033L15 | 1110033L15Rik | 0.958 |  |  |
| Similar to teratocarcinoma expressed, serine rich | LOC224276 | 0.958 |  | XM_147217 |
| DNA segment, Chr 9, ERATO Doi 280, expressed | D9Ertd280e | 0.958 | D930024E11 | NM_177775 |
| Deoxyhypusine synthase | Dhps | 0.958 | Dhs;MGC49129;MGC74384 | NM_201408 |
| Cytochrome c oxidase, subunit VIIc | Cox7c | 0.958 | Cox7c1;COXVIIc | NM_007749 |
| Fat tumor suppressor homolog (Drosophila) | Fath | 0.957 |  | XM_134149 |
| cDNA sequence BC002230 | BC002230 | 0.957 |  | NM_183155 |
| RIKEN cDNA 0610010D24 | 0610010D24Rik | 0.956 |  | NM_026681 |
| L(3)mbt-like 2 (Drosophila) | L3mbtl2 | 0.956 | 4732493N06Rik;M4mbt;MGC31247 | NM_145993 |
| Similar to RNP particle component | LOC386124 | 0.956 |  | XM_359080 |
| Glycerol kinase (Gyk), transcript variant 2 | Gyk | 0.956 | GK | NM_008194 |
| Neuronal pentraxin 2 | Nptx2 | 0.956 | narp;np2 | NM_016789 |
| Carbonyl reductase 3 | Cbr3 | 0.956 | 1110001J05Rik | NM_173047 |
| Synaptogyrin 3 | Syngr3 | 0.955 |  | NM_011522 |
| Phosphomannomutase 1 | Pmm1 | 0.955 |  | NM_013872 |
| Hypothetical LOC237436 | LOC237436 | 0.955 |  | XM_137276 |
| Exportin 6 | Xpo6 | 0.955 | C230091E20Rik;mKIAA0370;Ranbp20 | NM_028816 |
| Transcriptional adaptor 2 (ADA2 homolog, yeast)-like | Tada2l | 0.955 | AV319371;D030022J10;D030022J10Rik | NM_172562 |
| DEAD (Asp-Glu-Ala-Asp) box polypeptide 25 | Ddx25 | 0.955 | GRTH | NM_013932 |
| Tight junction protein 2 | Tjp2 | 0.954 | ZO-2 | NM_011597 |
| Collapsin response mediator protein 1 | Crmp1 | 0.954 | Dpysl1;DRP-1;Ulip3 | NM_007765 |
| Branched chain aminotransferase 2, mitochondrial | Bcat2 | 0.954 | Bcat-2;Eca40 | NM_009737 |
| DNA segment, Chr 5, 0834 expressed | D5Bwg0834e | 0.954 | MGC38387 | NM_144819 |
| Expressed sequence C79127 | C79127 | 0.954 | A930002H20 | NM_177691 |
| Opioid receptor, sigma 1 | Oprs1 | 0.954 | mSigmaR1 | NM_011014 |
| High mobility group box 2-like 1 | Hmgb2l1 | 0.954 | 4733401K04Rik;E430025G12 | NM_178017 |
| RIKEN cDNA 2900011O08 | 2900011O08Rik | 0.954 |  | NM_144518 |
| Ubiquitin specific protease 10 | Usp10 | 0.954 | mKIAA0190;UBPO;Uchrp | NM_009462 |
| DNA segment, Chr 7, Roswell Park 2 complex | D7Rp2e | 0.954 | D7Rp2;D7Rp2-r;D7Rp2-s;RP2-r;RP2-s | NM_033080 |
| Glutamate dehydrogenase 1 | Glud1 | 0.954 |  | NM_008133 |
| RIKEN cDNA 6330580J24 | 6330580J24Rik | 0.953 |  | NM_026378 |
| Engulfment and cell motility 1, ced-12 homolog (C. elegans) | Elmo1 | 0.953 | 6330578D22Rik;C230095H21Rik;CED-12 | NM_198093 |
| Oxidation resistance 1 | Oxr1 | 0.953 | 2210416C20Rik;C7;C7B | AK040881 |
| Bromodomain and PHD finger containing, 1 | Brpf1 | 0.953 | 4833438B11Rik;4930540D11Rik | NM_030178 |
| Methylenetetrahydrofolate dehydrogenase | Mthfd1 | 0.953 | DCS;E430024A07Rik;Mthfd;NEUT2 | NM_138745 |
| Similar to teratocarcinoma expressed, serine rich | Tera-pending | 0.953 | Tera | XM_125257 |
| Polymerase (RNA) III (DNA directed) polypeptide E | Polr3e | 0.953 |  | NM_025298 |
| RIKEN cDNA 2310051N18 | 2310051N18Rik | 0.953 |  | XM_134026 |
| RIKEN cDNA 6330514A18 | 6330514A18Rik | 0.953 | 6330514A18 | NM_183152 |
| ATPase type 13A | Atp13a | 0.952 | catp;Cgi152 | NM_133224 |
| Exosome component 2 | Exosc2 | 0.952 | MGC30456;Rrp4 | NM_144886 |
| Suppression of tumorigenicity 14 (colon carcinoma) | St14 | 0.952 | Epithin;MT-SP1;Prss14 | NM_011176 |
| RIKEN cDNA 9830004M20 | 9830004M20Rik | 0.952 | AW108387 | NM_175347 |
| DNA methyltransferase 3B | Dnmt3b | 0.952 |  | NM_010068 |
| Hspb associated protein 1 | Hspbap1 | 0.952 | 3830421G21Rik | NM_175111 |
| DNA segment, Chr 5 0834 expressed | D5Bwg0834e | 0.952 | MGC38387 | NM_144819 |
| Actinin alpha 3 | Actn3 | 0.952 |  | NM_013456 |
| RIKEN cDNA 9630037P07 | 9630037P07Rik | 0.952 |  | XM_149022 |
| RiIKEN full-length enriched library, clone:B930098N04 |  | 0.952 |  | AK081191 |
| RIKEN full-length enriched library, clone:D130049D24 |  | 0.951 |  | AK051443 |
| Expressed sequence AI429152 | AI429152 | 0.951 | MGC25461 | NM_153126 |
| Formin 2 | Fmn2 | 0.951 |  | NM_019445 |
| Branched chain aminotransferase 1, cytosolic | Bcat1 | 0.951 | Bcat-1;BCATc;Eca39 | NM_007532 |
| Tubulin, alpha 4 | Tuba4 | 0.951 | M[a]4 | NM_009447 |
| RIKEN cDNA 9430023L20 | 9430023L20Rik | 0.951 |  | NM_026566 |
| Small nuclear RNA activating complex, polypeptide 4 | Snapc4 | 0.951 | 5730436L13;5730436L13Rik | NM_172339 |
| Zinc finger protein 365 | Zfp365 | 0.951 | 6330417K12;mKIAA0844 | NM_178679 |
| cDNA sequence BC023823 | BC023823 | 0.951 | MGC38336 | NM_153566 |
| RIKEN cDNA E430034L04 | E430034L04Rik | 0.950 | G3BP;G3BP2 | NM_011816 |
| RIKEN cDNA G430022H21 | G430022H21Rik | 0.950 | mKIAA1627 | NM_201638 |
| Similar to hypothetical protein MGC15668 | LOC242642 | 0.950 |  | XM_143930 |
| Similar to GTP-binding protein ragB | LOC245670 | 0.950 |  | XM_142239 |
| Sprouty homolog 4 (Drosophila) | Spry4 | 0.950 | A030006O18Rik;sprouty4 | NM_011898 |
| RIEKN cDNA 9530025L08 | 9530025L08Rik | 0.950 |  |  |
| Dead end homolog 1 (zebrafish) | Dnd1 | 0.950 | MGC41452;RBMS4 | NM_173383 |
| Expressed sequence AW060766 | AW060766 | 0.950 | A130090N03 | NM_198033 |
| Exportin, tRNA (nuclear export receptor for tRNAs) | Xpot | 0.950 |  | XM_125902 |
| RIKEN cDNA 1700028N11 | 1700028N11Rik | 0.949 |  | NM_029341 |
| Ribosomal protein L38 | Rpl38 | 0.949 | 0610025G13Rik | NM_023372 |
| Deltex 1 homolog (Drosophila) | Dtx1 | 0.949 | Fxit1 | NM_008052 |
| Monogenic, audiogenic seizure susceptibility 1 | Mass1 | 0.949 |  | AK053565 |
| cDNA sequence BC020354 | BC020354 | 0.949 |  | NM_198110 |
| RAS protein activator like 1 (GAP1 like) | Rasal1 | 0.949 | MRASAL | NM_013832 |
| Leucine rich repeat and fibronectin type III domain containing 1 | Lrfn1 | 0.949 | MGC7599 | NM_030562 |
| RIKEN cDNA B230113M03 | B230113M03Rik | 0.949 |  | XM_109923 |
| Hyaluronan and proteoglycan link protein 4 | Hapln4 | 0.949 | 9330174O11;Bral2;Lpr4 | NM_177900 |
| 4-nitrophenylphosphatase domain P25-like protein homolog 1 | Nipsnap1 | 0.949 |  | NM_008698 |
| Aminoacylase 1 | Acy1 | 0.949 | 1110014J22Rik;Acy-1 | NM_025371 |
| RIKEN cDNA 2310005L22 | 2310005L22Rik | 0.949 |  |  |
| Similar to hypothetical protein BC008207 | LOC238136 | 0.949 |  | XM_138060 |
| Tnf receptor-associated factor 6 | Traf6 | 0.948 | 2310003F17Rik;C630032O20Rik | NM_009424 |
| Solute carrier family 3, member 2 | Slc3a2 | 0.948 | Mdu1;Mgp-2hc | NM_008577 |
| Absent in melanoma 1-like | Aim1l | 0.948 |  | XM_131754 |
| Debranching enzyme homolog 1 | Dbr1 | 0.948 |  | NM_031403 |
| RIKEN cDNA A230102I05 | A230102I05Rik | 0.948 | MGC31575 | NM_146179 |
| cDNA sequence BC017634 | BC017634 | 0.948 | MGC28873 | NM_145621 |
| Mannose-6-phosphate receptor, cation dependent | M6pr | 0.948 | CD-MPR;Mpr46 | NM_010749 |
| Mdn1 | Mdn1 | 0.948 |  | NM_133874 |
| RIKEN cDNA 2310004L02 | 2310004L02Rik | 0.948 | C030044E10Rik | NM_025504 |
| Transcriptional adaptor 2 | Tada2l | 0.948 | AV319371;D030022J10;D030022J10Rik | NM_172562 |
| Phospholipase A2, group XIIA | Pla2g12a | 0.947 | GXII;MGC58884;mGXII-1;mGXII-1-PLA2;Pla2g12 | NM_023196 |
| Tryptophanyl-tRNA synthetase | Wars | 0.947 | WRS | NM_011710 |
| Serum/glucocorticoid regulated kinase | Sgk | 0.947 | Sgk1 | NM_011361 |
| Solute carrier family 5 (sodium iodide symporter), member 5 | Slc5a5 | 0.947 | NIS | NM_053248 |
| Phosphoribosyl pyrophosphate synthetase-associated protein 1 | Prpsap1 | 0.946 |  | XM_181343 |
| Pleckstrin homology domain containing, family F member 2 | Plekhf2 | 0.946 | 1110070J07Rik;ZFYVE18 | NM_175175 |
| LONE NT2RP7008406 | 5730455A04Rik | 0.946 |  | AK077577 |
| Solute carrier family 27 (fatty acid transporter), member 2 | Slc27a2 | 0.946 | FATP2;Vlac;Vlacs;VLCS | NM_011978 |
| Solute carrier family 34 (sodium phosphate), member 3 | Slc34a3 | 0.946 | NptIIc | NM_080854 |
| Downregulated in Zic1 deficient cerebellum | Dorz1 | 0.946 | 1110013B16Rik | NM_145919 |
| ATP-binding cassette, sub-family F (GCN20), member 2 | Abcf2 | 0.946 | D13Ertd614e;E430001O06 | NM_013853 |
| Transducin-like enhancer of split 4, homolog of Drosophila E(spl) | Tle4 | 0.946 | Bce-1;Bce1;ESTM13;ESTM14;Grg4;X83333 | NM_011600 |
| Excision repair cross-complementing rodent repair deficiency, 3 | Ercc3 | 0.946 | Ercc-3;XPB | NM_133658 |
| RIKEN cDNA 3110030K17 | 3110030K17Rik | 0.945 |  | XM_131770 |
| Transcription factor CP2-like 3 | Tcfcp2l3 | 0.945 | 0610015A08Rik;BOM;Grhl2 | NM_026496 |
| YTH domain family 1 | Ythdf1 | 0.945 |  | NM_173761 |
| Suppression of tumorigenicity 14 (colon carcinoma) | St14 | 0.945 | Epithin;MT-SP1;Prss14 | NM_011176 |
| Gamma-aminobutyric acid (GABA-A) receptor, subunit beta 3 | Gabrb3 | 0.945 | A230092K12Rik;Cp1;Gabrb-3 | NM_008071 |
| Calcitonin/calcitonin-related polypeptide, alpha | Calca | 0.945 | CA;Calc;Calc1;Cgrp;CGRP-1;CGRP1;Ct;Ctn | NM_007587 |
| Cytokine receptor-like factor 1 | Crlf1 | 0.945 | CLF-1;CRLM3;NR6.1 | NM_018827 |
| Ubiquitin specific protease 7 | Usp7 | 0.944 |  | XM_148584 |
| Solute carrier family 25, member 15 | Slc25a15 | 0.944 | D630044L02Rik;Ornt1 | NM_181325 |
| RIKEN cDNA 4930449I23 | 4930449I23Rik | 0.944 |  | XM_132052 |
| RIKEN cDNA 1110008J03 | 1110008J03Rik | 0.944 | AI853657 | NM_029096 |
| Zinc finger, SWIM domain containing 1 | Zswim1 | 0.944 | 2410003H12Rik | NM_028028 |
| Makorin, ring finger protein, 1 | Mkrn1 | 0.944 |  | NM_018810 |
| RIKEN cDNA 4930424G05 | 4930424G05Rik | 0.944 |  | NM_026251 |
| Guanine nucleotide binding protein, beta polypeptide 1-like | Gnb1l | 0.944 | ESTM55;Wdr14;Wdvcf | NM_023120 |
| DiGeorge syndrome critical region gene 8 | Dgcr8 | 0.943 | D16H22S1742E;D16H22S788E;D16Wis2;Gy1;N41 | NM_033324 |
| Lymphocyte protein tyrosine kinase | Lck | 0.943 | Hck-3;p56<lck> | NM_010693 |
| Adherin 1 | Cdh1 | 0.943 | E-cadherin;Ecad;Um;UVO;uvomorulin | AK041755 |
| Unknown (protein for MGC:58818) | MGC58818 | 0.943 |  | NM_198637 |
| RIKEN cDNA 9030409E16 | 9030409E16Rik | 0.943 |  | NM_025781 |
| RIKEN cDNA E130115I21 | E130115I21Rik | 0.943 |  |  |
| RIKEN cDNA 2810453I06 | 2810453I06Rik | 0.943 | 1500026F15Rik | NM_026050 |
| Aquarius | Aqr | 0.943 | mKIAA0560 | NM_009702 |
| CHK2 checkpoint homolog (S. pombe) | Chek2 | 0.943 | Cds1;CHK2;HUCDS1;Rad53 | NM_016681 |
| Protein phosphatase 1B, magnesium dependent, beta isoform | Ppm1b | 0.943 | PP2CB | NM_011151 |
| Exosome component 5 | Exosc5 | 0.943 | D7Wsu180e | NM_138586 |
| Expressed sequence AI849286 | AI849286 | 0.943 |  | XM_194372 |
| ER1 (FRAGMENT) | E030034J16Rik | 0.942 |  | AK087212 |
| TAR (HIV) RNA binding protein 2 | Tarbp2 | 0.942 | Prbp | NM_009319 |
| PDZ domain containing 4 | Pdzk4 | 0.942 |  | XM_359337 |
| RIKEN cDNA 9630044O09 | 9630044O09Rik | 0.942 | AA675320 | NM_198014 |
| CXXC finger 6, transcript variant 1 | Cxxc6 | 0.942 |  | XM_125673 |
| Vclin C | Ccnc | 0.942 | CG1C | AK037957 |
| RIKEN cDNA B930007L02 | B930007L02Rik | 0.942 |  | XM_356186 |
| Tight junction protein 2 | Tjp2 | 0.942 | ZO-2 | NM_011597 |
| ULFOTRANSFERASE-RELATED PROTEIN | 2400007A17Rik | 0.942 |  | AK010293 |
| Serum/glucocorticoid regulated kinase | Sgk | 0.941 | Sgk1 | NM_011361 |
| RIKEN cDNA C130020C07 | C130020C07Rik | 0.941 |  | AK047886 |
| Additional sex combs like 1 | Asxl1 | 0.941 |  | XM_149245 |
| DNA (cytosine-5-)-methyltransferase 3-like | Dnmt3l | 0.941 | D6Ertd14e | NM_019448 |
| Leucine zipper-EF-hand containing transmembrane protein 1 | Letm1 | 0.941 |  | NM_019694 |
| Internexin neuronal intermediate filament protein, alpha | Ina | 0.941 | MGC25352;NF-66 | NM_146100 |
| TAR (HIV) RNA binding protein 2 | Tarbp2 | 0.941 | Prbp | NM_009319 |
| Myc induced nuclear antigen | Mina | 0.941 | 1810047J07Rik;2410057H13Rik;3830408E23Rik | NM_025910 |
| Paired basic amino acid cleaving system 4 | Pace4 | 0.941 |  | XM_355911 |
| Similar to NADPH-DEPENDENT FMN AND FAD OXIDOREDUCTASE | 4930447P04Rik | 0.941 |  | AK019617 |
| Expressed sequence AI838661 | AI838661 | 0.941 |  | NM_133884 |
| RIKEN cDNA 4732473B16 | 4732473B16Rik | 0.940 | AU045678 | NM_175307 |
| RIKEN cDNA A830021K08 | A830021K08Rik | 0.940 |  |  |
| Surfeit gene 6 | Surf6 | 0.940 | D2Wsu129e;Surf-6 | NM_009298 |
| Transaldolase 1 | Taldo1 | 0.940 |  | NM_011528 |
| Tripartite motif protein 28 | Trim28 | 0.940 | KAP-1;KRIP-1;Tif1b;Tif1beta | NM_011588 |
| cDNA sequence BC004022 | BC004022 | 0.940 | MGC7607;N4bp1 | XM_134557 |
| Torsin family 3, member A | Tor3a | 0.940 | Adir | NM_023141 |
| cDNA sequence BC003281 | BC003281 | 0.940 | Bagl;Bal;MGC7868 | NM_030253 |
| Expressed sequence C86302 | C86302 | 0.940 | B130036O03 | NM_172746 |
| Methionine adenosyltransferase II, alpha | Mat2a | 0.940 | D630045P18Rik;MGC6545 | NM_145569 |
| Stathmin-like 2 | Stmn2 | 0.940 | SCG10;Scgn10;Stmb2 | NM_025285 |
| Elongation factor RNA polymerase II-like 3 | Ell3 | 0.940 | A930015D22Rik;MGC31450 | NM_145973 |
| Similar to RIKEN cDNA 2410116I05 | LOC385959 | 0.940 |  | XM_359014 |
| RNA polymerase 1-2 | Rpo1-2 | 0.940 | 128kDa;D630020H17Rik;RPA116;RPA135;RPA2 | NM_009086 |
| Replication factor C (activator 1) 5 | Rfc5 | 0.940 | 2610020K06Rik | XM_132348 |
| RIKEN full-length enriched library, clone:E430018M12 |  | 0.940 |  | AK088483 |
| SWI/SNF related, actin dependent regulator of chromatin, a3 | Smarca3 | 0.939 | P113;Snf2l3 | NM_009210 |
| Ubiquinol-cytochrome c reductase core protein 1 | Uqcrc1 | 0.939 | 1110032G10Rik | NM_025407 |
| Von Willebrand factor A domain containing 2 | Amaco | 0.939 |  | NM_172840 |
| Expressed sequence AA408556 | AA408556 | 0.939 | mKIAA0690 | NM_199447 |
| ATP-binding cassette, sub-family F (GCN20), member 1 | Abcf1 | 0.939 | Abc50;D17Wsu166e;GCN20 | NM_013854 |
| Phospholipase A2, group XIIA | Pla2g12a | 0.939 | GXII;MGC58884;mGXII-1;mGXII-1-PLA2;Pla2g12 | NM_023196 |
| Adaptor protein complex AP-1, mu 2 subunit | Ap1m2 | 0.938 | [m]1B;D9Ertd818e;mu1B | NM_009678 |
| Purine-nucleoside phosphorylase | Pnp | 0.938 | Np;Np-1;Np-2 | NM_013632 |
| RIKEN cDNA 1700007E06 | 1700007E06Rik | 0.938 |  |  |
| Aldehyde dehydrogenase 4 family, member A1 | Aldh4a1 | 0.938 | ALDH4;E330022C09;P5CDH;P5CDhL;P5CDhS | NM_175438 |
| Growth factor receptor bound protein 7 | Grb7 | 0.938 |  | NM_010346 |
| RIKEN cDNA 9630033F20 | 9630033F20Rik | 0.938 |  | NM_177003 |
| RIKEN cDNA F830014G06 | F830014G06Rik | 0.938 |  | AK089746 |
| CCR4-NOT transcription complex, subunit 10 | Cnot10 | 0.937 | 2600001P13Rik;MGC37693 | NM_153585 |
| Translocase of outer mitochondrial membrane 40 homolog | Tomm40 | 0.937 | Mom35;Tom40 | NM_016871 |
| THAP domain containing, apoptosis associated protein 1 | Thap1 | 0.937 | 4833431A01Rik | NM_199042 |
| Chromodomain helicase DNA binding protein 5 | Chd5 | 0.937 |  | XM_196334 |
| SECIS binding protein 2 | Secisbp2 | 0.937 |  | XM_127336 |
| Adaptor protein complex AP-1, mu 2 subunit | Ap1m2 | 0.937 | [m]1B;D9Ertd818e;mu1B | NM_009678 |
| RIKEN cDNA 4933424A10 | 4933424A10Rik | 0.937 |  | NM_177293 |
| ATP-binding cassette, sub-family C (CFTR/MRP), member 4 | Abcc4 | 0.936 |  | XM_139262 |
| Pipecolic acid oxidase | Pipox | 0.936 | LPIPOX;Pso | NM_008952 |
| RIKEN cDNA 2410075B13 | 2410075B13Rik | 0.936 | MGC37569 | NM_146059 |
| Integrin, alpha E, epithelial-associated | Itgae | 0.936 | A530055J10;alpha-E1;CD103 | NM_008399 |
| RIKEN cDNA 9130210N20 | 9130210N20Rik | 0.936 | 9130210N20 | NM_172607 |
| UDP-GlcNAc:betaGal beta-1,3-N-acetylglucosaminyltransferase 7 | B3gnt7 | 0.936 | beta-3GnT7;C330001H22Rik | NM_145222 |
| Preferentially expressed antigen in melanoma like 4 | Pramel4 | 0.936 |  | NM_178248 |
| Cytidine 5-triphosphate synthase | Ctps | 0.936 |  | NM_016748 |
| DIRAS family, GTP-binding RAS-like 1 | Diras1 | 0.936 | Gbts1;RIG | NM_145217 |
| Pleckstrin homology domain containing, family F member 2 | Plekhf2 | 0.935 | 1110070J07Rik;ZFYVE18 | NM_175175 |
| Pre-mRNA processing factor 8 | Prpf8 | 0.935 | D11Bwg0410e;DBF3/PRP8;Prp8;Sfprp8l | NM_138659 |
| RIKEN cDNA 3110082D06 gene | 3110082D06Rik | 0.935 |  | XM_140020 |
| Laminin, alpha 5 | Lama5 | 0.935 |  | XM_203796 |
| G1 to phase transition 2 | Gspt2 | 0.935 |  | NM_008179 |
| Neighbor of Cox4 | Noc4 | 0.935 |  | NM_010926 |
| Solute carrier family 25, member 26 | Slc25a26 | 0.934 | 4933433F13Rik;D6Bwg0781e;Slc25a6 | NM_026255 |
| Phospholipase A2, group XIIA | Pla2g12a | 0.934 | GXII;MGC58884;mGXII-1;mGXII-1-PLA2;Pla2g12 | NM_023196 |
| TG interacting factor | Tgif | 0.934 |  | NM_009372 |
| Serine/threonine kinase 4 | Stk4 | 0.934 | Kas-2;Mst1;Ysk3 | NM_021420 |
| Sulfotransferase family 4A, member 1 | Sult4a1 | 0.934 | 2400007A17Rik;BR-STL-1;Sultx3 | NM_013873 |
| CCR4-NOT transcription complex, subunit 3 | Cnot3 | 0.934 | MGC40675 | NM_146176 |
| RIKEN cDNA 2310075G12 | 2310075G12Rik | 0.934 | 1110014L05Rik | NM_027162 |
| Hypothetical GTP1/OBG family containing protein | 2810405J23Rik | 0.934 |  | AK012998 |
| Ubiquitin specific protease 7 | Usp7 | 0.934 |  | NM_001003918 |
| Kelch domain containing 4 | Klhdc4 | 0.934 |  | NM_145605 |
| Rab geranylgeranyl transferase, a subunit | Rabggta | 0.934 | gm | NM_019519 |
| Cytochrome c-1 | Cyc1 | 0.934 | 2610002H19Rik | NM_025567 |
| IKEN full-length enriched library, clone:1110054O05 |  | 0.934 |  | AK004256 |
| MutS homolog 6 (E. coli) | Msh6 | 0.934 | GTBP;Gtmbp | NM_010830 |
| Glycosyltransferase 28 domain containing 1 | Glt28d1 | 0.934 |  | NM_026247 |
| Poly (ADP-ribose) glycohydrolase | Parg | 0.934 |  | NM_011960 |
| NADPH dependent diflavin oxidoreductase 1 | Ndor1 | 0.933 |  | NM_178239 |
| N-ethylmaleimide sensitive fusion protein attachment protein beta | Napb | 0.933 | b-SNAP;Brp14;E161;I47;SNARE | NM_019632 |
| RIKEN cDNA C030019I05 | C030019I05Rik | 0.933 |  | NM_177075 |
| RIKEN cDNA A930004K21 | A930004K21Rik | 0.933 |  | NM_172673 |
| RIKEN cDNA 0610039J04 | 0610039J04Rik | 0.933 |  | XM_134310 |
| Sterile alpha motif domain containing 10 | Samd10 | 0.933 |  | NM_172676 |
| Galactose-1-phosphate uridyl transferase | Galt | 0.933 |  | NM_016658 |
| Zinc finger protein 598 | Zfp598 | 0.933 |  | NM_183149 |
| BTB (POZ) domain containing 3 | Btbd3 | 0.933 | MGC25591;mKIAA0952 | NM_145534 |
| RIKEN cDNA 2310057J16 | 2310057J16Rik | 0.933 |  | XM_133997 |
| Lipin 3 | Lpin3 | 0.933 | 9130206L11Rik | NM_022883 |
| Ribosomal protein S6 kinase-like 1 | Rps6kl1 | 0.933 | A830084F09Rik;MGC38756 | NM_146244 |
| Nucleoporin 210 | Nup210 | 0.933 | 9830001L10;gp190;gp210;Pom210 | NM_018815 |
| Low density lipoprotein receptor-related protein 11 | Lrp11 | 0.933 | 6330533B21;9830160H19Rik | NM_172784 |
| Nucleotide binding protein 2 | Nubp2 | 0.933 | D17Wsu11e | NM_011956 |
| Hook homolog 2 (Drosophila) | Hook2 | 0.933 | A630054I03Rik;MGC28586 | NM_133255 |
| Branched chain aminotransferase 1, cytosolic | Bcat1 | 0.933 | Bcat-1;BCATc;Eca39 | NM_007532 |
| RAN guanine nucleotide release factor | Rangnrf | 0.933 | 2400006H24Rik;Mog1 | NM_021329 |
| Disabled homolog 1 (Drosophila) | Dab1 | 0.933 | scm;scr;scrambler;yot | NM_010014 |
| Integrin, alpha E, epithelial-associated | Itgae | 0.933 | A530055J10;alpha-E1;CD103 | NM_008399 |
| RIKEN cDNA 1700063H04 | 1700063H04Rik | 0.932 |  | XM_132752 |
| Insulin degrading enzyme | Ide | 0.932 | 1300012G03Rik;4833415K22Rik | NM_031156 |
| POU domain, class 3, transcription factor 1 | Pou3f1 | 0.932 | Oct-6;Otf6;Scip;Test1;Tst-1 | NM_011141 |
| RIKEN cDNA 2810439M11 | 2810439M11Rik | 0.932 |  | NM_183091 |
| Similar to ACY1L2 protein | LOC242377 | 0.932 |  | XM_143698 |
| Similar to hypothetical protein FLJ36878 | LOC380705 | 0.932 |  | XM_354618 |
| Uridine phosphorylase 1 | Upp1 | 0.932 | UdRPase;Up;UPase;Upp | NM_009477 |
| RIKEN cDNA 4833439L19 | 4833439L19Rik | 0.932 | 4930558H15Rik;C81457 | NM_133797 |
| Eukaryotic translation initiation factor 4, gamma 1 | Eif4g1 | 0.932 |  | XM_358318 |
| RIKEN cDNA 2310008H09 | 2310008H09Rik | 0.932 | Tsg118 | NM_023197 |
| EBNA1 binding protein 2 | Ebna1bp2 | 0.932 | 1810014B19Rik;Ebp2;Nobp;p40 | NM_026932 |
| DOT1-like, histone H3 methyltransferase (S. cerevisiae) | Dot1l | 0.931 | A630076O07;mDot1 | NM_199322 |
| Similar to Transmembrane gamma-carboxyglutamic acid protein 3 | LOC208748 | 0.931 |  | XM_141833 |
| Glutaminase 2 | Gls2 | 0.931 |  | XM_125928 |
| Zinc finger protein 106 | Zfp106 | 0.931 | Cd-1;H3a | NM_011743 |
| Bromodomain containing 2 | Brd2 | 0.931 | D17H6S113E;Frg-1;Fsrg1;NAT;Ring3;Rnf3 | NM_010238 |
| Diptheria toxin resistance protein | Dph2l2 | 0.931 | 9130020C19Rik | NM_026344 |
| Forkhead box N4 | Foxn4 | 0.931 |  | NM_148935 |
| cDNA sequence BC060631 | BC060631 | 0.931 | MGC79206;mKIAA0431 | NM_177700 |
| Cat eye syndrome chromosome region, candidate 5 homolog | Cecr5 | 0.931 | MGC25951 | NM_144815 |
| Orthodenticle homolog 2 (Drosophila) | Otx2 | 0.931 | E130306E05Rik | NM_144841 |
| Hspb associated protein 1 | Hspbap1 | 0.931 | 3830421G21Rik | NM_175111 |
| Cytochrome P450, family 2, subfamily j, polypeptide 13 | Cyp2j13 | 0.931 |  | NM_145548 |
| RIKEN cDNA 2310010I15 | 2310010I15Rik | 0.930 |  | AK009272 |
| Ubiquitin carboxy-terminal hydrolase L1 | Uchl1 | 0.930 | gad;PGP9.5 | NM_011670 |
| Solute carrier family 12 (potassium/chloride transporters), member 8 | Slc12a8 | 0.930 | CCC9;E330020C02Rik | NM_134251 |
| Matrix metalloproteinase 17 | Mmp17 | 0.930 | MT4-MMP | NM_011846 |
| Synaptotagmin 5 | Syt5 | 0.930 | Syt9;SytIX;SytV | NM_016908 |
| MYB binding protein (P160) 1a | Mybbp1a | 0.930 | P160 | NM_016776 |
| Hypothetical LOC333190 | LOC333190 | 0.930 |  | XM_289489 |
| RIKEN cDNA 1700094G20 | 1700094G20Rik | 0.930 |  | AK007064 |
| Hypothetical protein E130320D18 | Dock5 | 0.929 |  | NM_172811 |
| Alanyl-tRNA synthetase | Aars | 0.929 | MGC37368 | NM_146217 |
| Transcription factor-like 5 | Tcfl5 | 0.929 | Figlb | NM_178254 |
| Undifferentiated embryonic cell transcription factor 1 | Utf1 | 0.929 |  | NM_009482 |
| Dual-specificity tyrosine-(Y)-phosphorylation regulated kinase 3 | Dyrk3 | 0.929 |  | NM_145508 |
| RIKEN cDNA A630097K09 | A630097K09Rik | 0.929 |  | AK042505 |
| Hypothetical Ankyrin repeat profile/Ankyrin repeat region circular | D030049N18Rik | 0.929 |  | AK050983 |
| Mitochondrial ribosomal protein L4 | Mrpl4 | 0.929 | 1110017G11Rik | NM_023167 |
| RIKEN cDNA 3830422N12 | 3830422N12Rik | 0.929 | 3830422N12;NY-SAR-35;NYSAR35 | NM_174993 |
| Treacher Collins Franceschetti syndrome 1, homolog | Tcof1 | 0.929 |  | NM_011552 |
| cDNA sequence BC024969 | BC024969 | 0.929 |  | XM_140497 |
| NOL1/NOP2/Sun domain family 2 | Nsun2 | 0.928 |  | NM_145354 |
| Terminal uridylyl transferase 1, U6 snRNA-specific | Rbm21 | 0.928 |  | NM_197993 |
| Glutathione S-transferase, alpha 4 | Gsta4 | 0.928 | mGsta4 | NM_010357 |
| Regulating synaptic membrane exocytosis 3 | Rims3 | 0.928 | A730060M23Rik;mKIAA0237;Nim3;Rim3 | NM_182929 |
| RAD51-like 3 (S. cerevisiae) | Rad51l3 | 0.928 | R51H3;Rad51d | NM_011235 |
| Tumor suppressing subtransferable candidate 1 | Tssc1 | 0.928 |  | NM_201357 |
| Replication protein A2 | Rpa2 | 0.928 | Rf-A2 | NM_011284 |
| Collapsin response mediator protein 5 | Crmp5-pending | 0.927 |  | AK082132 |
| Ring finger protein 134 | Rnf134 | 0.927 | 4933407A11Rik;MBLR | NM_027654 |
| PWP2 (periodic tryptophan protein) homolog, yeast | Pwp2h | 0.927 | 6530411D08Rik;Pwp2;wdp103 | NM_029546 |
| Ectonucleoside triphosphate diphosphohydrolase 6 | Entpd6 | 0.927 | 2700026H11Rik;Cd39l2;NTPDase-6 | NM_172117 |
| cDNA sequence BC052885 | 6530405K19 | 0.927 |  | NM_183021 |
| Anaplastic lymphoma kinase | Alk | 0.927 | CD246;Tcrz | NM_007439 |
| ATX1 (antioxidant protein 1) homolog 1 (yeast) | Atox1 | 0.927 | ATX1 | NM_009720 |
| Immunoglobulin superfamily, member 9 | Igsf9 | 0.927 | 644ETD8;Kiaa1355-hp;mKIAA1355;Ncaml;NRT1 | NM_033608 |
| U3 small nucleolar ribonucleoprotein, homolog A | Utp14a | 0.927 |  | XM_135857 |
| Polymerase (RNA) III (DNA directed) polypeptide B | Polr3b | 0.926 |  | NM_027423 |
| RIKEN cDNA 2810453I06 | 2810453I06Rik | 0.926 | 1500026F15Rik | NM_026050 |
| Sstathmin-like 3 | Stmn3 | 0.926 | Sclip | NM_009133 |
| Hypoxia up-regulated 1 | Hyou1 | 0.926 | Cab140;CBP-140;Grp170;Orp150 | NM_021395 |
| Suppressor of cytokine signaling 2 | Socs2 | 0.926 | 8030460M17;CIS2;Cish2;hg;JAB;SOCS-2;SSI-2 | NM_007706 |
| Dynamin 1-like | Dnm1l | 0.926 | 6330417M19Rik;Drp1 | NM_152816 |
| Biquitin specific protease 26 | Usp26 | 0.926 |  | AK082829 |
| Uracil-DNA glycosylase | Ung | 0.926 | UNG1;UNG2 | NM_011677 |
| RIKEN cDNA C330016H24 | C330016H24Rik | 0.926 | C630038K21Rik | NM_026563 |
| BCL2-associated athanogene 4 | Bag4 | 0.926 | 2410112I15Rik;SODD | NM_026121 |
| RIKEN cDNA 2010009L17 | 2010009L17Rik | 0.926 | 2310029P06Rik;Peg3 | NM_025630 |
| Nuclear transcription factor-Y beta | Nfyb | 0.926 | Cbf-A | NM_010914 |
| Expressed sequence AA408296 | AA408296 | 0.926 |  | NM_145415 |
| Peroxisome proliferative activated receptorcoactivator1 | Pprc1 | 0.925 |  | XM_359412 |
| cDNA sequence BC027061 | BC027061 | 0.925 |  | NM_183165 |
| Rho GDP dissociation inhibitor (GDI) gamma | Arhgdig | 0.925 | Gdi5;Rho-GDI-3;Rho-GDI2;RIP2 | NM_008113 |
| RIKEN full-length enriched library, clone:A430076C13 |  | 0.925 |  | AK079817 |
| Testis expressed gene 10 | Tex10 | 0.925 | clone 18330;MGC11792 | NM_172304 |
| Kelch domain containing 4 | Klhdc4 | 0.925 |  | NM_145605 |
| Hypothetical PHD-finger containing protein | 2010009J12Rik | 0.925 |  | AK008164 |
| RIKEN cDNA 4833439L19 | 4833439L19Rik | 0.925 | 4930558H15Rik;C81457 | NM_133797 |
| Nuclear factor of activated T-cells, calcineurin-dependent 2 | Nfatc2 | 0.925 | NFAT1;NFAT1-D;Nfatp | NM_010899 |
| RIKEN cDNA 2900011O08 | 2900011O08Rik | 0.925 |  | NM_144518 |
| RIKEN cDNA C030023A16 | C030023A16Rik | 0.925 |  | AK047745 |
| Tripartite motif protein 13 | Trim13 | 0.925 | 3110001L12Rik;LEU5;Rfp2 | NM_023233 |
| RNA (guanine-9-) methyltransferase domain containing 2 | Rg9mtd2 | 0.924 | 3110023L08Rik;Rnmtd2 | NM_175389 |
| RIKEN cDNA 4632409L19 | 4632409L19Rik | 0.924 |  | XM_355376 |
| RIKEN cDNA E430023L22 | E430023L22Rik | 0.924 |  | AK088688 |
| RIEKN cDNA B230208H11 | B230208H11Rik | 0.924 |  |  |
| Similar to mKIAA0133 protein | LOC382038 | 0.924 |  | XM_356112 |
| RNA polymerase I transcription factor homolog | Rrn3 | 0.924 |  | XM_358764 |
| Protein phosphatase 1A, alpha isoform | Ppm1a | 0.924 | 2900017D14Rik;MMPa-2;MPPa-1 | NM_008910 |
| Hypothetical Glycosyl transferase, family 2 containing protein | B230205C01Rik | 0.924 |  | AK045461 |
| Peroxiredoxin 1 | Prdx1 | 0.924 | NkefA;OSF-3;PAG;Paga;PrxI;Tdpx2;TDX2;TPx-A | NM_011034 |
| Phospholipase A2, group IVB (cytosolic) | Pla2g4b | 0.923 |  | XM_358347 |
| Phospholipase A2, group VI | Pla2g6 | 0.923 |  | NM_016915 |
| RIKEN cDNA 1110033C18 gene | 1110033C18Rik | 0.923 | AI585913 | NM_133964 |
| Suppressor of cytokine signaling 4 | Socs4 | 0.923 | 3110032M18Rik;Socs7 | NM_080843 |
| RIKEN cDNA 9930116P15 | 9930116P15Rik | 0.923 |  | XM_127961 |
| ATPase, H+/K+ transporting, alpha polypeptide | Atp4a | 0.923 |  | NM_018731 |
| RIKEN cDNA 5830415F09 | 5830415F09Rik | 0.923 |  | XM_131394 |
| Hypothetical Low density lipoprotein (LDL)-receptor class A | 6430526J12Rik | 0.923 |  | AK032360 |
| RIKEN cDNA 2010204K13 | 2010204K13Rik | 0.923 | 0610010M13Rik;Cip7 | AK008437 |
| UDP-glucuronosyltransferase 8 | Ugt8 | 0.922 | Cgt | NM_011674 |
| Solute carrier family 25 (mitochondrial carrier: glutamate), member 22 | Slc25a22 | 0.922 | 1300006L01Rik | NM_026646 |
| Mitogen-activated protein kinase kinase kinase 9 | Map3k9 | 0.922 | E130314H24Rik;Mlk1;Prke1 | NM_177395 |
| Flavoprotein oxidoreductase MICAL3 | Mical3 | 0.922 | C130040D16Rik;MICAL-3;mKIAA1364 | NM_153396 |
| Ubiquitin specific peptidase 36, transcript variant 1 | Usp36 | 0.922 |  | XM_126772 |
| Adhesion regulating molecule 1 | Adrm1 | 0.922 | 2510006J17Rik;ARM-1;Arm1;Gp110 | NM_019822 |
| Neighbor of Cox4 | Noc4 | 0.922 |  | NM_010926 |
| Additional sex combs like 1 | LOC228790 | 0.922 |  | XM_149245 |
| RIKEN cDNA 9230117E06 | 9230117E06Rik | 0.922 |  |  |
| Galactose-1-phosphate uridyl transferase | Galt | 0.921 |  | NM_016658 |
| RIKEN cDNA 4933425K02 | 4933425K02Rik | 0.921 | 1700094G20Rik;4933424B12Rik | AK015318 |
| RIKEN cDNA 2310003C21 | 2310003C21Rik | 0.921 |  |  |
| RIKEN cDNA A930009M04 | A930009M04Rik | 0.921 |  | NM_177266 |
| Regulating synaptic membrane exocytosis 2 | Rims2 | 0.921 | mKIAA0751;Rab3ip2;RIM2;Serg2;Syt3-rs | NM_053271 |
| Pleckstrin homology domain containing, family K member 1 | Plekhk1 | 0.921 | B130039D23Rik;Mbf;RTKN2 | NM_133244 |
| RIKEN cDNA 5730438N18 | 5730438N18Rik | 0.921 |  | NM_027460 |
| SET domain-containing protein 7 | Set7 | 0.921 | 1600028F23Rik;mKIAA1717 | NM_080793 |
| Similar to transcriptional factor | LOC234741 | 0.921 |  | XM_134580 |
| Similar to prepro-Neuropeptide W polypeptide | LOC381073 | 0.921 |  | XM_354994 |
| RIKEN cDNA 1300013J15 | 1300013J15Rik | 0.921 |  | NM_026183 |
| DNA segment, Chr 4, ERATO Doi 22, expressed | D4Ertd22e | 0.921 | 1110022I03 | NM_174996 |
| cDNA sequence BC024537 | BC024537 | 0.921 | MGC37588 | NM_146237 |
| Spermine synthase | Sms | 0.920 | Gy;gyro | NM_009214 |
| Expressed sequence AI527266 | AI527266 | 0.920 |  | XM_132830 |
| RIKEN cDNA D030041I09 | D030041I09Rik | 0.920 | C330007D07;mKIAA0479 | NM_175460 |
| Phosphoribosyl pyrophosphate synthetase 1 | Prps1 | 0.920 | 2310010D17Rik;Prps-1 | NM_021463 |
| RIKEN cDNA 4933406J04 | 4933406J04Rik | 0.920 | AI507137 | NM_175333 |
| RIKEN full-length enriched library, clone:2400008K01 |  | 0.920 |  | AK075923 |
| SEC23B (S. cerevisiae) | Sec23b | 0.920 |  | NM_019787 |
| RIKEN cDNA 2700083B06 | 2700083B06Rik | 0.920 |  | NM_026531 |
| Inositol 1,3,4-triphosphate 5/6 kinase | Itpk1 | 0.920 |  | NM_172584 |
| Adaptor protein complex AP-1, mu 2 subunit | Ap1m2 | 0.920 | [m]1B;D9Ertd818e;mu1B | NM_009678 |
| Hypothetical gene supported by AK087313 | LOC381223 | 0.920 |  | XM_358529 |
| Envoplakin | Evpl | 0.920 |  | NM_025276 |
| Eukaryotic translation initiation factor 2B, subunit 2 beta | Eif2b2 | 0.920 | EIF-2Bbeta;EIF2B | NM_145445 |
| 3-hydroxybutyrate dehydrogenase (heart, mitochondrial) | Bdh | 0.920 | 2310032J20Rik | NM_175177 |
| DEAH (Asp-Glu-Ala-His) box polypeptide 8 | Dhx8 | 0.919 | Ddx8;mDEAH6;MGC31290 | NM_144831 |
| Ankyrin repeat domain 27 (VPS9 domain) | Ankrd27 | 0.919 | D330003H11Rik;MGC25907;Varp | NM_145633 |
| RIKEN cDNA 9130011J04 | 9130011J04Rik | 0.919 |  |  |
| Rap guanine nucleotide exchange factor (GEF) 1 | Rapgef1 | 0.919 | 4932418O06Rik;C3G;Grf2 | NM_054050 |
| RIKEN cDNA 9430034D17 | 9430034D17Rik | 0.919 |  | NM_029891 |
| GA repeat binding protein, alpha | Gabpa | 0.919 |  | NM_008065 |
| Protein tyrosine phosphatase, receptor type, F | Ptprf | 0.919 | LAR;LARS | NM_011213 |
| RIKEN cDNA 6030408C04 | 6030408C04Rik | 0.919 |  | XM_126912 |
| Seven in absentia 1B | Siah1b | 0.919 | Sinh1b | NM_009173 |
| RIKEN cDNA D630039M01 | D630039M01Rik | 0.919 |  | AK052733 |
| Cell division cycle 25 homolog A (S. cerevisiae) | Cdc25a | 0.919 |  | NM_007658 |
| Protein tyrosine phosphatase-like p, member b | Ptplb | 0.919 | 6330408J20Rik | NM_023587 |
| RIKEN cDNA 2810017I02 | 2810017I02Rik | 0.918 |  |  |
| Early growth response 4 | Egr4 | 0.918 | NGF1-C;NGFI-C;NGFIC;pAT133 | NM_020596 |
| nucleoporin 93 | Nup93 | 0.918 | 2410008G02Rik | NM_172410 |
| RIKEN cDNA 2610304F09 | 2610304F09Rik | 0.918 | 6720406D06 | NM_172965 |
| MutS homolog 2 (E. coli) | Msh2 | 0.918 |  | NM_008628 |
| Zinc finger protein 259 | Zfp259 | 0.918 | ZPR1 | NM_011752 |
| Sialyltransferase 7 | Siat7b | 0.918 | Siat7 | NM_009180 |
| RIKEN cDNA 2410022L05 | 2410022L05Rik | 0.918 | 1700029I03Rik;MGC11316 | NM_025556 |
| Dystrophin | Dmd | 0.918 | Dp427;DXSmh7;DXSmh9;mdx;pke | NM_007868 |
| RIKEN cDNA B130016L12 | B130016L12Rik | 0.918 |  | NM_144835 |
| Cyclin-dependent kinase-like 2 | Cdkl2 | 0.917 | 5330436L21Rik;KKIAMRE;Kkm | NM_177270 |
| RIKEN cDNA C130064E22 | C130064E22Rik | 0.917 |  | AK048477 |
| Uracil-DNA glycosylase | Ung | 0.917 | UNG1;UNG2 | NM_011677 |
| RIKEN cDNA A330066M24 | A330066M24Rik | 0.917 |  | XM_355247 |
| DEAH (Asp-Glu-Ala-His) box polypeptide 37 | Dhx37 | 0.917 |  | NM_203319 |
| Nitrilase family, member 2 | Nit2 | 0.917 |  | NM_023175 |
| RIKEN cDNA C330016K18 | C330016K18Rik | 0.917 |  | XM_132642 |
| RIKEN cDNA 6330579B17 | 6330579B17Rik | 0.917 | AI790434 | NM_026494 |
| DEAD (Asp-Glu-Ala-Asp) box polypeptide 20 | Ddx20 | 0.917 | dp103;GEMIN3 | NM_017397 |
| CTD-BINDING SR-LIKE PROTEIN RA4 homolog | B130053I10Rik | 0.917 |  | AK045266 |
| RIKEN cDNA 2410004F06 | 2410004F06Rik | 0.917 | 2410070K17Rik | NM_028034 |
| Potassium channel, subfamily K, member 1 | Kcnk1 | 0.917 | TWIK-1 | NM_008430 |
| Glutathione S-transferase omega 1 | Gsto1 | 0.917 | GSTX;p28 | NM_010362 |
| RIKEN cDNA 4933428G09 | 4933428G09Rik | 0.917 |  | NM_025755 |
| G elongation factor | Gfm | 0.917 | D3Wsu133e | NM_138591 |
| ATP-binding cassette, sub-family F (GCN20), member 1 | Abcf1 | 0.917 | Abc50;D17Wsu166e;GCN20 | NM_013854 |
| RIKEN cDNA 3632413B07 | 3632413B07Rik | 0.916 | 2010005I16Rik;MGC25852;mKIAA1125;Prkcbp1 | NM_172270 |
| RIKEN cDNA D030014N22 | D030014N22Rik | 0.916 | 4932441F21 | NM_178734 |
| Hypothetical RNA-binding region RNP-1 | A430091O22Rik | 0.916 | 4732493M14 | AK040404 |
| Procollagen C-endopeptidase enhancer 2 | Pcolce2 | 0.916 | 2400001O18Rik;Pcpe2 | NM_029620 |
| Hexokinase 2 | Hk2 | 0.916 | HKII | NM_013820 |
| Cyclic nucleotide gated channel beta 1b | Cngb1b | 0.916 |  | XM_286113 |
| Density-regulated protein | Denr | 0.916 | 1500003K04Rik | NM_026603 |
| Mitochondrial transcription termination factor | MTERF | 0.916 |  | NM_172135 |
| RIKEN cDNA E130309D02 | E130309D02Rik | 0.916 | A630028N22 | NM_172726 |
| Dystrobrevin alpha, transcript variant 2 | Dtna | 0.916 | A0;adbn;Dtn | NM_207650 |
| Signaling intermediate in Toll pathway-evolutionarily conserved | Sitpec | 0.916 | ECSIT | NM_012029 |
| Hypothetical RNA-binding region RNP-1 | 4921532D18Rik | 0.916 |  | AK014994 |
| Mitochondrial ribosomal protein S25 (Mrps25) | Mrps25 | 0.916 | 2810429N01Rik;Rpms25 | NM_025578 |
| Seven in absentia 1B | Siah1b | 0.916 | Sinh1b | NM_009173 |
| Fat tumor suppressor homolog (Drosophila) | Fath | 0.916 |  | XM_134149 |
| Brain acyl-CoA hydrolase | Bach | 0.916 | 2410041A17Rik;ACH1;ACT;CTE-II;LACH1 | NM_133348 |
| SET domain, bifurcated 1 | Setdb1 | 0.916 | ESET | NM_018877 |
| Metallothionein 2 | Mt2 | 0.915 | Mt-2;MT-II | NM_008630 |
| RIKEN cDNA 2500003M10 | 2500003M10Rik | 0.915 |  | NM_023215 |
| Chromodomain helicase DNA binding protein 1-like | Chd1l | 0.915 | 4432404A22Rik;Snf2p | NM_026539 |
| Ring finger protein 44 | Rnf44 | 0.915 | AI854545;mKIAA1100 | NM_134064 |
| Mannose-P-dolichol utilization defect 1 | Mpdu1 | 0.915 | LEC35;SL15;Supl15h | NM_011900 |
| RIKEN cDNA A030007L17 | A030007L17Rik | 0.915 |  | NM_026637 |
| Poly(A) binding protein, nuclear 1 | Pabpn1 | 0.915 | mPABII;PAB2;Pabp3 | NM_019402 |
| Protein phosphatase 1G (formerly 2C), gamma isoform | Ppm1g | 0.915 | Fin13 | NM_008014 |
| Dystrophin | dystrophin | 0.915 |  | M68859 |
| Retinol binding protein 7, cellular | Rbp7 | 0.914 | 1110002J23Rik;CRBP-III | NM_022020 |
| Exportin 5 | Xpo5 | 0.914 | 2410004H11Rik;2700038C24Rik;RanBp21 | NM_028198 |
| Myeloid leukemia factor 2 | Mlf2 | 0.914 |  | NM_145385 |
| RNA, U3 small nucleolar interacting protein 2 | Rnu3ip2 | 0.914 | 55kDa;D9Wsu10e;MGC25949;U3-55k | NM_145620 |
| Armadillo repeat gene deleted in velo-cardio-facial syndrome | Arvcf | 0.914 |  | AK053865 |
| Zcchc7 | Zcchc7 | 0.914 |  | NM_138590 |
| A kinase (PRKA) anchor protein (gravin) 12 | Akap12 | 0.914 | Srcs5;SSeCKS;Tsga12 | NM_031185 |
| RAN guanine nucleotide release factor | Rangnrf | 0.914 | 2400006H24Rik;Mog1 | NM_021329 |
| Carboxy-terminal domain, RNA polymerase II, polypeptide A, subunit 1 | Ctdp1 | 0.914 | 4930563P03Rik | NM_026295 |
| Mitochondrial ribosomal protein L38 | Mrpl38 | 0.914 | MGC41062;MGC8310;MRP-L3;Rpml3 | AK013126 |
| RAN GTPase activating protein 1 | Rangap1 | 0.914 | Fug1 | NM_011241 |
| Expressed sequence C80913 | C80913 | 0.914 | NNX3;Rmp | NM_011274 |
| Polymerase (RNA) II (DNA directed) polypeptide I | Polr2i | 0.914 |  | XM_133304 |
| Branched chain aminotransferase 1, cytosolic | Bcat1 | 0.913 | Bcat-1;BCATc;Eca39 | NM_007532 |
| Cytochrome P450, family 11, subfamily a, polypeptide 1 | Cyp11a1 | 0.913 | Cyp11a;Cypxia1;D9Ertd411e;P450scc | NM_019779 |
| Transcription elongation factor A (SII), 3 | Tcea3 | 0.913 | S-II | NM_011542 |
| Loop tail associated protein | Ltap | 0.913 | Kiaa1215;loopLp;Lpp1;stbm;Vang1l2;Vangl2 | NM_033509 |
| Deoxyribonuclease II alpha | Dnase2a | 0.913 | Dnase2 | NM_010062 |
| Replication protein A1 | Rpa1 | 0.913 | 5031405K23Rik;70kDa;RF-A;RP-A;Rpa | NM_026653 |
| RIKEN cDNA 2600005C20 | 2600005C20Rik | 0.913 | D030064A17;Kiaa0179;mKIAA0179 | NM_028244 |
| Bromodomain adjacent to zinc finger domain, 2A | Baz2a | 0.913 | C030005G16Rik;mKIAA0314;Tip5;Walp3 | NM_054078 |
| Zinc finger protein 259 | Zfp259 | 0.913 | ZPR1 | AK044472 |
| Deoxyribonuclease II alpha | Dnase2a | 0.913 | Dnase2 | NM_010062 |
| Qtrtd1 | Qtrtd1 | 0.913 |  | XM_148491 |
| Adaptor protein with pleckstrin homology and src | Aps-pending | 0.913 |  | AK017604 |
| Aryl hydrocarbon receptor nuclear translocator | Arnt | 0.913 | D3Ertd557e;Drnt;ESTM42;Hif1b | AK037762 |
| Tubulin, alpha 6 | Tuba6 | 0.912 | M[a]6 | XM_147357 |
| RIKEN cDNA 2610024N01 | 2610024N01Rik | 0.912 |  | NM_027931 |
| Helicase (DNA) B | Helb | 0.912 | D10Ertd664e | NM_080446 |
| Transcriptional regulator, SIN3A (yeast) | Sin3a | 0.912 | Sin3 | NM_011378 |
| RIKEN cDNA 2700097O09 | 2700097O09Rik | 0.912 |  | NM_028314 |
| Nanos homolog 1 (Drosophila) | Nanos1 | 0.912 |  | NM_178421 |
| Mitochondrial ribosomal protein S2 | Mrps2 | 0.912 |  | NM_080452 |
| Protein kinase C and casein kinase substrate in neurons 1 | Pacsin1 | 0.912 | A830061D09Rik;H74;mKIAA1379;syndapin | NM_011861 |
| RIKEN cDNA 1700007K13 | 1700007K13Rik | 0.912 |  | XM_130125 |
| RIKEN cDNA A630077B13 | A630077B13Rik | 0.912 |  | NM_175449 |
| Growth differentiation factor 1 | Gdf1 | 0.912 | Gdf-1;UOG-1 | NM_008107 |
| Sphingomyelin phosphodiesterase, acid-like 3B | Smpdl3b | 0.911 | 1110054A24Rik;Asml3b | NM_133888 |
| Microspherule protein 1 | Mcrs1 | 0.911 | ICP22BP;MSP58;P78 | NM_016766 |
| Thioredoxin-like 4 | Txnl4 | 0.911 | Dim1;U5-15kD;U5-15kDa | NM_025299 |
| RIKEN cDNA 1810015A11 | 1810015A11Rik | 0.911 |  | XM_148046 |
| RIKEN cDNA 2510003B16 | 2510003B16Rik | 0.911 |  |  |
| ArsA (bacterial) arsenite transporter, ATP-binding, homolog 1 | Asna1 | 0.911 | 1810048H22Rik;ArsA | NM_019652 |
| Protein phosphatase 1B, magnesium dependent, beta isoform | Ppm1b | 0.910 | PP2CB | NM_011151 |
| G two S phase expressed protein 1 | Gtse1 | 0.910 | B99;Gtse-1 | NM_013882 |
| Phosphoribosylglycinamide formyltransferase | Gart | 0.910 | Gaps;Prgs | NM_010256 |
| Nudix (nucleoside diphosphate linked moiety X)-type motif 1 | Nudt1 | 0.910 | Mth1 | NM_008637 |
| Neutrophil cytosolic factor 1 | Ncf1 | 0.910 | Ncf-1;NOXO2;p47<phox>;p47phox | NM_010876 |
| RIKEN cDNA 2310044G17 | 2310044G17Rik | 0.910 | mKIAA1737 | NM_173735 |
| RIKEN cDNA 2810422O20 | 2810422O20Rik | 0.910 |  | NM_027279 |
| Ataxin 7-like 3, transcript variant 1 | Atxn7l3 | 0.910 |  | XM_126779 |
| Mitochondrial ribosomal protein S18B | Mrps18b | 0.910 | 2400002C15Rik | NM_025878 |
| Frizzled homolog 9 (Drosophila) | Fzd9 | 0.910 |  | XM_284144 |
| DNA segment, Chr 2, ERATO Doi 145, expressed | D2Ertd145e | 0.910 | 5730435J01Rik;6530403D07Rik;Rif1 | NM_175238 |
| RIKEN cDNA 1700018O18 | 1700018O18Rik | 0.910 |  | XM_131683 |
| Tyrosine kinase, non-receptor, 1 | Tnk1 | 0.910 | Kos1 | NM_031880 |
| Gamma-aminobutyric acid (GABA-A) receptor, subunit beta 3 | Gabrb3 | 0.909 | A230092K12Rik;Cp1;Gabrb-3 | NM_008071 |
| Expressed sequence C86987 | C86987 | 0.909 |  | XM_127523 |
| RIKEN cDNA 2610018I03 | 2610018I03Rik | 0.909 |  | XM_135023 |
| Nuclear factor of activated T-cells, calcineurin-dependent 2 interacting | Nfatc2ip | 0.909 | NIP45 | NM_010900 |
| RIKEN cDNA 1810019J16 | 1810019J16Rik | 0.909 |  | NM_133707 |
| Unc-13 homolog B | Unc13b | 0.909 |  | NM_021468 |
| Tripartite motif protein 27 | Trim27 | 0.909 | Rfp | NM_009054 |
| Similar to Aldo-keto reductase family 1, member B3 | LOC216674 | 0.909 |  | XM_126090 |
| Cyclin C | Ccnc | 0.909 | CG1C | AK009615 |
| RIKEN cDNA 2610020N02 | 2610020N02Rik | 0.909 |  | NM_181470 |
| Small nuclear ribonucleoprotein polypeptide A | Snrpa | 0.908 | Rnu1a-1;Rnu1a1;U1A | NM_015782 |
| RIKEN cDNA A330067K20 | A330067K20Rik | 0.908 |  | AK039587 |
| RIKEN cDNA E430018M08 | E430018M08Rik | 0.908 | 5830436H09 | NM_173445 |
| Ubiquitination factor E4A, UFD2 homolog (S. cerevisiae) | Ube4a | 0.908 | 4732444G18Rik;9930123J21Rik;UFD2b | NM_145400 |
| Ectodermal-neural cortex 1 | Enc1 | 0.908 | Nrpb;PIG10 | NM_007930 |
| RIKEN cDNA 6330503K22 | 6330503K22Rik | 0.908 | AI427129 | NM_182995 |
| RIKEN cDNA 9130604K18 | 9130604K18Rik | 0.908 |  | AK078977 |
| Ring finger protein 126 | Rnf126 | 0.908 | 2610010O19Rik | NM_144528 |
| Solute carrier family 7, member 5 | Slc7a5 | 0.908 | D0H16S474E;TA1 | NM_011404 |
| RIKEN cDNA 1700027M01 | 1700027M01Rik | 0.908 | 1110056G13Rik;9130022E05Rik | NM_023544 |
| RIKEN cDNA 2610103J23 | 2610103J23Rik | 0.908 | D8Bwg1112e;Lyric | NM_026002 |
| RIKEN cDNA A830039B04 | A830039B04Rik | 0.908 |  | NM_177045 |
| TGFB-induced factor 2 | Tgif2 | 0.908 | 4921501K24;5730599O09Rik | NM_173396 |
| Endonuclease G | Endog | 0.908 |  | NM_007931 |
| Upstream binding transcription factor, RNA polymerase I | Ubtf | 0.908 | NOR-90;Tcfubf;UBF;UBF1 | NM_011551 |
| RIKEN cDNA 2410085M17 | 2410085M17Rik | 0.907 |  |  |
| Excision repair cross-complementing rodent repair deficiency, | Ercc2 | 0.907 | Ercc-2;XPD | NM_007949 |
| DEAD (Asp-Glu-Ala-Asp) box polypeptide 24 | Ddx24 | 0.907 | 1700055J08Rik;2510027P10Rik | NM_020494 |
| TATA box binding protein -associated factor, RNA polymerase I, C | Taf1c | 0.907 | mTAFI95;Tafi95 | NM_021441 |
| Argininosuccinate synthetase 1 | Ass1 | 0.907 | ASS;Ass-1 | NM_007494 |
| RIKEN cDNA D930009B21 | D930009B21Rik | 0.907 |  | AK086163 |
| RIKEN cDNA B130016L12 | B130016L12Rik | 0.907 |  | NM_144835 |
| Myosin VC, transcript variant 1 | Myo5c | 0.907 |  | XM_198225 |
| Hypothetical Ankyrin repeat profile | A130082N24Rik | 0.907 |  | AK038156 |
| RIKEN cDNA 3200002M19 | 3200002M19Rik | 0.907 | 6330414C15Rik | NM_027532 |
| Weakly similar to CYTOHESIN 4 | 5830469K17Rik | 0.906 |  | AK030956 |
| Similar to IQ motif containing GTPase activating protein 3 | LOC383946 | 0.906 |  | XM_357341 |
| RIKEN full-length enriched library, clone:E030019A03 |  | 0.906 |  | AK053156 |
| RIEKN cDNA 1110049N09 | 1110049N09Rik | 0.906 |  | AK004213 |
| ATP-binding cassette, sub-family F (GCN20), member 2 | Abcf2 | 0.906 | 0710005O05Rik;D13Ertd614e;E430001O06 | NM_013853 |
| Neuropathy target esterase | Nte | 0.906 | MSws | NM_015801 |
| Epiplakin 1 | Eppk1 | 0.906 | 6230424I18Rik;EPIPL;EPIPL1 | NM_144848 |
| Expressed sequence AI462438 | AI462438 | 0.906 | Heab | NM_133840 |
| RIKEN cDNA 1810022K09 | 1810022K09Rik | 0.906 |  | XM_485210 |
| SKB1 homolog (S. pombe) | Skb1 | 0.906 | Jbp1 | NM_013768 |
| RIKEN cDNA 1500002M01 | 1500002M01Rik | 0.906 |  | NM_133702 |
| Zinc finger protein 64 | Zfp64 | 0.905 |  | NM_009564 |
| RIKEN cDNA 2610528H13 | 2610528H13Rik | 0.905 | MGC38132 | NM_145944 |
| XPA binding protein 1 | Xab1 | 0.905 | 2410004J02Rik;MBDIN;NTPBP | NM_133756 |
| Gem (nuclear organelle) associated protein 4 | Gemin4 | 0.905 | 4932415L08Rik;MGC28146 | NM_177367 |
| RIKEN cDNA 5730410I19 | 5730410I19Rik | 0.905 |  | NM_025666 |
| FK506 binding protein 11 | Fkbp11 | 0.905 | 1110002O23Rik | NM_024169 |
| Ribonuclease H1 | Rnaseh1 | 0.905 |  | NM_011275 |
| CD2 antigen (cytoplasmic tail) binding protein 2 | Cd2bp2 | 0.905 | 1500011B02Rik;2410024K20Rik | NM_027353 |
| Mitochondrial ribosomal protein S25 | Mrps25 | 0.905 | 2810429N01Rik;Rpms25 | NM_025578 |
| Expressed sequence AI118078 | AI118078 | 0.905 | 9630029F15 | NM_172923 |
| RIKEN cDNA 2610036A22 | 2610036A22Rik | 0.905 |  |  |
| RIKEN cDNA 1190002H09 | 1190002H09Rik | 0.905 |  | XM_355059 |
| Transcription factor 15 | Tcf15 | 0.905 | bHLH-EC2;Meso1;paraxis | NM_009328 |
| SDA1 domain containing 1 | Sdad1 | 0.905 | 4931421J16 | NM_172713 |
| RIKEN cDNA 9430051O21 | 9430051O21Rik | 0.905 |  |  |
| EBNA1 binding protein 2 | Ebna1bp2 | 0.905 | 1810014B19Rik;Ebp2;Nobp;p40 | NM_026932 |
| Proviral integration site 2 | Pim2 | 0.905 | DXCch3;Pim-2 | NM_138606 |
| Splicing factor, arginine/serine-rich 6 | Sfrs6 | 0.905 | 1210001E11Rik | NM_026499 |
| Gem (nuclear organelle) associated protein 5 | Gemin5 | 0.905 | C330013N08 | NM_172558 |
| Solute carrier family 29 (nucleoside transporters), member 2 | Slc29a2 | 0.905 | Der12;ENT2;HNP36 | NM_007854 |
| transformation related protein 53 | Trp53 | 0.905 | p53 | NM_011640 |
| Protein kinase C, alpha binding protein | Prkcabp | 0.905 | Pick1 | NM_008837 |
| RIKEN cDNA 5830467P10 | 5830467P10Rik | 0.904 | 5830467P10 | NM_198029 |
| Phosphatidylinositol transfer protein, cytoplasmic 1 | Pitpnc1 | 0.904 |  | NM_145823 |
| Similar to hypothetical protein FLJ12457 | LOC230805 | 0.904 |  | XM_144090 |
| EF hand calcium binding protein 2 | Efcbp2 | 0.904 | Necab2 | NM_054095 |
| major urinary protein 1 | Mup1 | 0.904 | 2610016E04Rik;Ltn-1;Lvtn-1;Mup-1;Mup-a;Up-1 | NM_031188 |
| RIKEN cDNA 1110025L05 | 1110025L05Rik | 0.904 |  | NM_175103 |
| RIKEN cDNA 2210013M04 | 2210013M04Rik | 0.904 |  | NM_178595 |
| TAF15 RNA polymerase II | Taf15 | 0.904 | 2610111C21Rik;68kDa;Taf2n;TAFII68 | NM_027427 |
| RIKEN cDNA 1500004F05 | 1500004F05Rik | 0.904 |  |  |
| Translocase of outer mitochondrial membrane 40 homolog | Tomm40 | 0.904 | Mom35;Tom40 | NM_016871 |
| Hypothetical KRAB box containing protein | C430020H24Rik | 0.904 |  | AK049536 |
| RASD family, member 2 | Rasd2 | 0.904 |  | XM_204287 |
| SH3 and cysteine rich domain 2 | Stac2 | 0.904 |  | NM_146028 |
| Rho-related BTB domain containing 2 | Rhobtb2 | 0.904 | Dbc2;E130206H14Rik;MGC28699;mKIAA0717 | NM_153514 |
| RIKEN cDNA 1190005I06 | 1190005I06Rik | 0.904 |  | NM_197988 |
| Nucleotide binding protein 2 | Nubp2 | 0.904 | D17Wsu11e | NM_011956 |
| Expressed sequence AU016977 | AU016977 | 0.903 | 9530023G02 | NM_175016 |
| Olfactory receptor 380 | Olfr380 | 0.903 | MOR135-1 | NM_147025 |
| RIKEN cDNA B230333C21 | B230333C21Rik | 0.903 |  | XM_111900 |
| CDK5 regulatory subunit associated protein 1 | Cdk5rap1 | 0.903 | 2310066P17Rik | NM_025876 |
| BCL2/adenovirus E1B 19kD interacting protein like | Bnipl | 0.903 | BNIP-S;BNIPL-1;BNIPL2;MGC8103;PP73;PP753 | NM_134253 |
| cDNA sequence BC021438 | BC021438 | 0.903 | MGC29361 | NM_145416 |
| cytochrome b-561 | Cyb561 | 0.903 |  | NM_007805 |
| RIKEN cDNA 2310075G12 | 2310075G12Rik | 0.903 | 1110014L05Rik | NM_027162 |
| Nucleolar protein 8 | Nol8 | 0.903 |  | XM_484255 |
| MARVEL (membrane-associating) domain containing 3 | Marveld3 | 0.903 |  | NM_028584 |
| Galanin receptor 2 | Galr2 | 0.903 | mGalR | NM_010254 |
| RIKEN cDNA 2300003C06 | 2300003C06Rik | 0.903 |  | NM_028001 |
| Surfeit gene 2 | Surf2 | 0.903 | Surf-2 | NM_013678 |
| Transducin-like enhancer of split 1, homolog of Drosophila E | Tle1 | 0.903 | C230057C06Rik;Grg1 | AK046402 |
| Jagged 2 | Jag2 | 0.902 | D12Ggc2e;mJagged2-1;Serh;sm | NM_010588 |
| Programmed cell death protein 11 | Pdcd11 | 0.902 |  | NM_011053 |
| Troponin C2, fast | Tnnc2 | 0.902 | Tncs | NM_009394 |
| RIKEN cDNA 2310008H09 | 2310008H09Rik | 0.902 | Tsg118 | NM_023197 |
| RIKEN cDNA 5730410I19 | 5730410I19Rik | 0.902 |  | NM_025666 |
| RIKEN cDNA 6030411F23 | 6030411F23Rik | 0.902 | B7H3;B7RP-2 | NM_133983 |
| Metallothionein 3 | Mt3 | 0.902 | MT-3 | NM_013603 |
| Phosphatase and tensin homolog | Pten | 0.902 | 2310035O07Rik;A130070J02Rik;MMAC1;TEP1 | AK051115 |
| Relaxin 1 | Rln1 | 0.902 |  | XM_129225 |
| RIKEN cDNA 9930116P15 | 9930116P15Rik | 0.902 |  | XM_127961 |
| RIKEN cDNA G430055L02 | G430055L02Rik | 0.902 |  | NM_145520 |
| RIKEN cDNA 4832412D13 | 4832412D13Rik | 0.902 |  | AK076431 |
| Embryonic lethal, abnormal vision, Drosophila-like 2 | Elavl2 | 0.902 | Hub;mel-N1 | NM_207686 |
| RIKEN cDNA 2310042N02 | 2310042N02Rik | 0.902 | 2310074C17Rik | NM_024246 |
| Interleukin 7 | Il7 | 0.902 | Il-7 | NM_008371 |
| ADP-ribosyltransferase (NAD+; poly (ADP-ribose) polymerase) 1 | Adprt1 | 0.902 | Adprp;C80510;PARP;parp-1;PARP1;sPARP-1 | NM_007415 |
| Kirsten rat sarcoma oncogene 2, expressed | Kras2 | 0.901 | K-ras;Ki-ras;Kras-2;MGC7141 | NM_021284 |
| RIKEN cDNA 2010001M06 | 2010001M06Rik | 0.901 |  |  |
| Similar to MSP23 | LOC234882 | 0.901 |  | XM_125096 |
| Zinc finger protein 219 | Zfp219 | 0.901 | 2010302A17Rik | NM_027248 |
| Cytochrome c oxidase assembly protein | Cox11 | 0.901 |  | NM_199008 |
| WD repeat domain 4 | Wdr4 | 0.901 | D530049K22Rik | NM_021322 |
| RIKEN cDNA 2010319A12 | 2010319A12Rik | 0.901 |  | AK008582 |
| RIKEN cDNA 1200009K13 | 1200009K13Rik | 0.901 | Pairbp1 | NM_025814 |
| Methyltransferase-like 1 | Mettl1 | 0.901 |  | NM_010792 |
| Similar to enhancer of invasion 10 | LOC239083 | 0.901 |  | XM_139038 |
| DNA methyltransferase 3B | Dnmt3b | 0.901 |  | AF151973 |
| Progressive external ophthalmoplegia 1 (human) | Peo1 | 0.901 | MGC41750;PEO;Twinl | NM_153796 |
| Phosphoribosylglycinamide formyltransferase | Gart | 0.901 | Gaps;Prgs | NM_010256 |
| Phospholipase A2, group X | Pla2g10 | 0.901 | mGXsPLA2;PLA2GX;sPLA2-X | XM_148336 |
| Choline kinase alpha | Chka | 0.900 | Chk;ChoK;EtnK-alpha | NM_013490 |
| Pleckstrin 2 | Plek2 | 0.900 |  | NM_013738 |
| cDNA sequence BC003281 | BC003281 | 0.900 | Bagl;Bal;MGC7868 | NM_030253 |
| Opioid receptor, sigma 1 | Oprs1 | 0.900 | mSigmaR1 | NM_011014 |
| SH3-domain GRB2-like 2 | Sh3gl2 | 0.900 | 9530001L19Rik;EEN-B1;Sh3d2a;SH3PA | NM_019535 |
| Bernardinelli-Seip congenital lipodystrophy 2 homolog | Bscl2 | 0.900 | 2900097C17Rik;Gng3lg;seipin | NM_008144 |
| Adaptor protein complex AP-2, alpha 1 subunit | Ap2a1 | 0.900 | Adtaa | NM_007458 |
| Matrix metallopeptidase 25 | Mmp25 | 0.900 |  | XM_139838 |
| Complexin 1 | Cplx1 | 0.900 | 921-S | NM_007756 |
| Transmembrane protein 8 (five membrane-spanning domains) | Tmem8 | 0.900 | M83 | NM_021793 |
