# Supplementary material for: Dynamic transcription programs during ES cell differentiation towards mesoderm in serum versus serum-freeBMP4 culture
Source: BMC Genomics. 2007 Oct 10;8:365. doi: 10.1186/1471-2164-8-365 (PMC2204012; doi:10.1186/1471-2164-8-365)
Supplement: Additional file 2 — Nanog gene list. The data provided lists all genes expressed during 16 days of embryoid body differentiation with similarity to Nanog (Pearson correlation >0.9). [file 1471-2164-8-365-S2.doc]

**Additional file 2:** Nanog gene list (Pearson correlation >0.9)

| **Description** | **Symbol** | **Corr.** | Synonyms | **Genbank ID** |
| --- | --- | --- | --- | --- |
| Nanog | Nanog | 1.00 |  | XM_132755.1 |
| Sortilin-related receptor, LDLR class A | Sorl1 | 0.98 | 2900010L19Rik;gp250;LR11;mSorLA | NM_011436 |
| CDP-diacylglycerol synthase 1 | Cds1 | 0.98 | 4833409J18Rik | NM_173370.3 |
| Rap1, GTPase-activating protein 1 | Rap1ga1 | 0.97 |  | XM_149500 |
| Epithelial V-like antigen 1 | Eva1 | 0.97 |  | NM_007962.2 |
| Tcf20 | Tcf20 | 0.96 | 2810438H08Rik;mKIAA0292;SPBP | NM_013836.2 |
| Connective tissue growth factor | Ctgf | 0.96 | Fisp12;Hcs24 | NM_010217 |
| Suppressor of cytokine signaling 2 | Socs2 | 0.96 | 8030460M17;CIS2;Cish2;hg;JAB;SOCS-2;SSI-2 | NM_007706.1 |
| 9330175B01Rik | 9330175B01Rik | 0.96 |  | XM_128267.4 |
| Sorting nexin 10 | Snx10 | 0.96 | 2410004M09Rik | NM_028035.2 |
| Endometrial bleeding associated factor | Ebaf | 0.95 | 6030463A22Rik;Lefta;Lefty2 | NM_177099.3 |
| Mcf.2 transforming sequence-like | Mcf2l | 0.95 | C130040G20Rik;Dbs;mKIAA0362 | NM_178076.2 |
| LIM-domain containing, protein kinase | Limk1 | 0.95 |  | NM_010717.1 |
| Lactate dehydrogenase 2, B chain | Ldh2 | 0.95 | Ldh-2;Ldhb | NM_008492.2 |
| Serine protease inhibitor, Kunitz type 2 | Spint2 | 0.95 | HAI-2 | NM_011464.1 |
| DnaJ (Hsp40) homolog, subfamily B, member 5 | Dnajb5 | 0.95 | 1110058L06Rik;Hsc40;Hsp40-3 | NM_019874.3 |
| L-lactate dehydrogenase B chain | LOC226017 | 0.95 |  | XM_129164.2 |
| Adenosine deaminase, RNA-specific | Adar | 0.95 | Adar1;mZaADAR | NM_019655.2 |
| 3-hydroxybutyrate dehydrogenase | Bdh | 0.95 | 2310032J20Rik | NM_175177 |
| Solute carrier family 4 (anion exchanger), member 8 | Slc4a8 | 0.95 | kNBC-3 | NM_021530 |
| Insulin-like growth factor binding protein 2 | Igfbp2 | 0.95 | Igfbp-2 | NM_008342.2 |
| RIKEN cDNA 4932417I16 | 4932417I16Rik | 0.94 |  | XM_134593.3 |
| Plakophilin 3 | Pkp3 | 0.94 | 2310056L12Rik | NM_019762.1 |
| Claudin 4 | Cldn4 | 0.94 | CEP-R;Cpetr;Cpetr1 | NM_009903.1 |
| Chromodomain helicase DNA binding protein 5 | Chd5 | 0.94 |  | XM_196334 |
| Cytochrome b-561 | Cyb561 | 0.94 |  | NM_007805.2 |
| Cell cycle related kinase | Ccrk | 0.94 |  | NM_053180.2 |
| ADP-ribosylation factor related protein 2 | Arfrp2 | 0.94 | A430036I03;C230032K13Rik | NM_172595.1 |
| Poliovirus receptor-related 2 | Pvrl2 | 0.94 | Cd112;MPH;nectin-2;Pvr;Pvs | NM_008990.2 |
| Hyperpolarization-activated, cyclic nucleotide-gated K+ 2 | Hcn2 | 0.94 | BCNG2;HAC1 | NM_008226.1 |
| RIKEN cDNA 4930555L03 | 4930555L03Rik | 0.94 |  | XM_204030.1 |
| SEC14-like 2 | Sec14l2 | 0.94 | 1300013M05Rik;TAP | NM_144520.1 |
| Serum/glucocorticoid regulated kinase | Sgk | 0.94 | Sgk1 | NM_011361.1 |
| Solute carrier family 7, member 4 | Slc7a4 | 0.93 | AI853530;MGC27672 | NM_144852 |
| Zinc finger CCCH type domain containing 1 | Zc3hdc1 | 0.93 | 9930021O16 | NM_172893.1 |
| RAS guanyl releasing protein 1 | Rasgrp1 | 0.93 | Rasgrp | NM_011246.2 |
| Microtubule-associated protein 7 | Mtap7 | 0.93 | E-MAP-115;MAP7 | NM_008635.1 |
| Erythrocyte protein band 4.1-like 4b | Epb4.1l4b | 0.93 | D4Ertd346e;Ehm2 | NM_019427 |
| Metallothionein 1 | Mt1 | 0.93 | Mt-1;MT-I | NM_013602.2 |
| Huntingtin-associated protein 1 , transcript variant 1 | Hap1 | 0.93 | HAP-1;MGC31449 | NM_177981.1 |
| SNF1-like kinase | Snf1lk | 0.93 | Msk | NM_010831.1 |
| Expressed sequence AW413632 | AW413632 | 0.93 | 9030212F24 | NM_177588.1 |
| Argininosuccinate synthetase 1 | Ass1 | 0.93 | ASS;Ass-1 | NM_007494.2 |
| Microtubule-associated protein 7 | Mtap7 | 0.93 | E-MAP-115;MAP7 | NM_008635 |
| FCH domain only 1 | Fcho1 | 0.93 | 3322402E17Rik | NM_028715.2 |
| Sortilin-related receptor, LDLR class A repeats-containing | Sorl1 | 0.93 | 2900010L19Rik;gp250;LR11;mSorLA | NM_011436 |
| Snail homolog 3 | Snai3 | 0.93 | Smuc;Zfp293 | NM_013914.2 |
| RIKEN cDNA 2610200G18 gene | 2610200G18Rik | 0.93 | 2810426C15Rik | NM_025998.1 |
| Aldehyde dehydrogenase 4 family, member A1 | Aldh4a1 | 0.93 | ALDH4;E330022C09;P5CD;P5CDH;P5CDhS | NM_175438 |
| Laminin, alpha 5 | Lama5 | 0.93 |  | XM_203796.1 |
| SH3 and cysteine rich domain 2 | Stac2 | 0.93 |  | NM_146028.2 |
| RIKEN cDNA 3830431G21 | 3830431G21Rik | 0.93 |  | XM_126991.3 |
| Epoxide hydrolase 2 | Ephx2 | 0.93 | Eph2 | NM_007940.2 |
| Cyclin M2 | Cnnm2 | 0.92 | Acdp2 | NM_033569.1 |
| Ras and Rab interactor 1 | Rin1 | 0.92 |  | NM_145495.1 |
| Similar to D52 | LOC234486 | 0.92 |  | XM_134462.2 |
| RIKEN cDNA 6820401H01 | 6820401H01Rik | 0.92 |  |  |
| RIKEN cDNA 1300010F03 | 1300010F03Rik | 0.92 |  | XM_127737.3 |
| Bone marrow stromal cell antigen 2 | Bst2 | 0.92 |  | NM_198095.1 |
| Mitogen activated protein kinase 13 | Mapk13 | 0.92 | SAPK4;Serk4 | NM_011950.1 |
| Argininosuccinate synthetase 1 | Ass1 | 0.92 | ASS;Ass-1 | NM_007494.2 |
| 3-hydroxybutyrate dehydrogenase | Bdh | 0.92 | 2310032J20Rik | NM_175177.3 |
| Cerebellin 3 precursor protein | Cbln3 | 0.92 |  | NM_019820.2 |
| RIKEN cDNA 3830422N12 | 3830422N12Rik | 0.92 | 3830422N12;NY-SAR-35;NYSAR35 | NM_174993.1 |
| Basic transcription element binding protein 1 | Bteb1 | 0.92 | BTEB-1;Klf9 | NM_010638.2 |
| RIKEN cDNA 5730592L21 | 5730592L21Rik | 0.92 |  | NM_029720.1 |
| Tight junction protein 2 | Tjp2 | 0.92 | ZO-2 | NM_011597.1 |
| Serum/glucocorticoid regulated kinase | Sgk | 0.92 | Sgk1 | NM_011361 |
| Growth arrest-specific 2 like 3 | Gas2l3 | 0.92 |  | XM_137276 |
| RIKEN cDNA 4933403L16 | 4933403L16Rik | 0.92 |  | XM_205476.3 |
| Transmembrane 4 superfamily member 5 | Tm4sf5 | 0.92 | 2010003F10Rik | NM_029360.1 |
| Myosin Va | Myo5a | 0.92 | Dbv;dilute;flail;flr;MVa;Myo5;MyoVA;Sev-1 | NM_010864.1 |
| Tumor protein p53 inducible protein 11 | Tp53i11 | 0.92 |  | XM_203859 |
| Serum response factor | Srf | 0.92 |  | NM_020493.1 |
| Thiosulfate sulfurtransferase, mitochondrial | Tst | 0.92 | Rhodanese | NM_009437.2 |
| cDNA sequence BC037135 | BC037135 | 0.92 | C430041M20 | NM_173763.2 |
| Amyotrophic lateral sclerosis 2 | Als2 | 0.92 | Als2cr6;MGC27807;mKIAA1563 | NM_028717.2 |
| Solute carrier family 27 (fatty acid transporter), member 2 | Slc27a2 | 0.92 | FATP2;Vlac;Vlacs;VLCS | NM_011978 |
| TBC1 domain family, member 2, transcript variant 1 | Tbc1d2 | 0.92 |  | XM_283964 |
| Mitogen activated protein kinase 8 interacting protein | Mapk8ip | 0.91 | IB1;JIP-1;MAPK8IP1;Prkm8ip | NM_011162.2 |
| RIKEN cDNA 2210013M04 | 2210013M04Rik | 0.91 |  | NM_178595.2 |
| Glycosyltransferase-like 1B | Gyltl1b | 0.91 | 5730485C17;5730485C17Rik;Largel | NM_172670.1 |
| RIKEN cDNA A930026I22 | A930026I22Rik | 0.91 |  |  |
| Spermine synthase | Sms | 0.91 | Gy;gyro | NM_009214.2 |
| RIKEN cDNA A430039L15 | Sec8 | 0.91 |  | AK039983 |
| Similar to KIAA1762 protein | LOC239102 | 0.91 |  | XM_139193.4 |
| Excision repair cross-complementing rodent repair deficiency group 4 | Ercc4 | 0.91 | Xpf | NM_015769.1 |
| Integrin alpha 6 | Itga6 | 0.91 | 5033401O05Rik;Cd49f | NM_008397 |
| Poly(rC) binding protein 3 | Pcbp3 | 0.91 | AlphaCP-3 | NM_021568.1 |
| Cingulin | Cgn | 0.91 |  | XM_131052.4 |
| Sema domain, immunoglobulin domain, transmembrane domain, 4A | Sema4a | 0.91 | Semab;SemB | NM_013658.2 |
| Glucosaminyl transferase 2, I-branching enzyme, transcript variant 3 | Gcnt2 | 0.91 | 5330430K10Rik;IGnTA;IGnTB;IGnTC | NM_008105.2 |
| Protein kinase C, zeta | Prkcz | 0.91 | Pkcz;zetaPKC | NM_008860.1 |
| Cadherin 1 | Cdh1 | 0.91 | E-cadherin;Ecad;Um;UVO;uvomorulin | NM_009864.1 |
| RIKEN cDNA 1600023A02 | 1600023A02Rik | 0.91 |  | NM_026323.1 |
| DNA segment, Chr 4, Brigham & Womens Genetics 0951 expressed | D4Bwg0951e | 0.91 | 1110029A09Rik;bM350F23.1 | NM_026821.2 |
| Leucine-rich repeat-containing 5 | Lrrc5 | 0.91 | 4930525N13Rik;A930019F03 | NM_178701.2 |
| Similar to hypothetical protein FLJ40362 | LOC381951 | 0.91 |  | XM_355977.1 |
| Rho/rac guanine nucleotide exchange factor (GEF) 18 | Arhgef18 | 0.91 | AI467246;D030053O22Rik | NM_133962.3 |
| Tight junction protein 3 | Tjp3 | 0.91 | ZO-3 | NM_013769.1 |
| Claudin 6 | Cldn6 | 0.91 |  | NM_018777.2 |
| RIKEN cDNA 6330530A05 gene | 6330530A05Rik | 0.91 | MGC48203 | NM_172383.1 |
| Receptor (calcitonin) activity modifying protein 3 | Ramp3 | 0.91 |  | NM_019511.1 |
| RWD domain containing 2 | Rwdd2 | 0.91 | 1700030C20Rik | NM_027100 |
| RIKEN cDNA 4931414L13 gene | 4931414L13Rik | 0.91 | AI448583 | NM_175321.2 |
| Villin 2 | Vil2 | 0.91 | cytovillin;ezrin;p81 | NM_009510.1 |
| DNA segment, Chr 19, ERATO Doi 144 | D19Ertd144e | 0.91 | 5830466O21Rik;Doc-1r | NM_026373.1 |
| Periplakin | Ppl | 0.91 | AW553870 | NM_008909 |
| Tight junction protein 2 | Tjp2 | 0.91 | ZO-2 | NM_011597 |
| SPARC related modular calcium binding 1 | Smoc1 | 0.90 | 2600002F22Rik;SRG | NM_022316.1 |
| Nanos homolog 1 (Drosophila) | Nanos1 | 0.90 |  | NM_178421.2 |
| Grancalcin | Gca | 0.90 | 5133401E04Rik | NM_145523.2 |
| RIKEN cDNA 0610039P13 | 0610039P13Rik | 0.90 | AI450555 | NM_028752.1 |
| Testis specific gene A2 | Tsga2 | 0.90 |  | NM_025290.2 |
| WAP four-disulfide core domain 2 | Wfdc2 | 0.90 |  | NM_026323.1 |
| RIKEN cDNA D030051N19 | D030051N19Rik | 0.90 | 2310079H06Rik;A130023A14;mKIAA1736 | NM_172669 |
| SH3 domain binding glutamic acid-rich protein like 2 | Sh3bgrl2 | 0.90 |  | NM_172507.2 |
| RIKEN cDNA 4930583C14 | 4930583C14Rik | 0.90 |  | NM_029472.1 |
| X-linked lymphocyte-regulated 3b | Xlr3a | 0.90 |  | NM_011726.1 |
| Similar to MAP-kinase phosphatase | LOC240672 | 0.90 |  | XM_140740.3 |
