# Supplementary material for: Dynamic transcription programs during ES cell differentiation towards mesoderm in serum versus serum-freeBMP4 culture
Source: BMC Genomics. 2007 Oct 10;8:365. doi: 10.1186/1471-2164-8-365 (PMC2204012; doi:10.1186/1471-2164-8-365)
Supplement: Additional file 3 — Sox2 gene list. The data provided lists all genes expressed during 16 days of embryoid body differentiation with similarity to Sox2 (Pearson correlation >0.9). [file 1471-2164-8-365-S3.doc]

**Additional file 3:** Sox2 gene list (Pearson correlation >0.9)

| **Description** | **Symbol** | **Corr.** | **Synonyms** | **Genbank ID** |
| --- | --- | --- | --- | --- |
| SRY-box containing gene 2 | Sox2 | 1.00 | Sox-2 | NM_011443.2 |
| Aryl hydrocarbon receptor nuclear translocator-like | Arntl | 0.974 | Arnt3;Bmal1;MOP3 | NM_007489.1 |
| L-threonine dehydrogenase | Tdh | 0.972 |  | NM_021480.4 |
| ADP-ribosylation factor guanine nucleotide-exchange factor 2 | Arfgef2 | 0.972 |  | XM_130646.3 |
| A kinase (PRKA) anchor protein 1 | Akap1 | 0.971 | Akap;C76494;C81186;S-AKAP84 | NM_009648.1 |
| Aminoadipate-semialdehyde synthase | Aass | 0.971 | LKR/SDH;LOR/SDH;Lorsdh | NM_013930.2 |
| Similar to RNI-like protein | LOC381907 | 0.97 |  | XM_355925.1 |
| Integrin, alpha E, epithelial-associated | Itgae | 0.97 | A530055J10;alpha-E1;CD103 | NM_008399.1 |
| RIKEN cDNA 3100002J23 | 3100002J23Rik | 0.97 |  | XM_484029 |
| RIKEN cDNA 4933406J07 gene | 4933406J07Rik | 0.968 |  | XM_133947.2 |
| Glucosaminyl (N-acetyl) transferase 2 | Gcnt2 | 0.968 | 5330430K10Rik;IGnTA;IGnTB;IGnTC | NM_008105.2 |
| Death inducer-obliterator 1 | Dido1 | 0.967 |  | XM_283823.2 |
| Repressor of GATA | Rog | 0.966 | 4930524C15Rik;FAXF;FAZF;Tzfp | NM_021397 |
| RIKEN cDNA 2410012C07 | 2410012C07Rik | 0.964 |  | XM_133920.3 |
| RIKEN cDNA 2410019P08 | D4Ertd432e | 0.964 |  | XM_112612.3 |
| Cordon-bleu | Cobl | 0.963 | 4732460E13;C530045F18Rik;mKIAA0633 | NM_172496.2 |
| Immunoglobulin-like domain containing receptor 1 | AU041483 | 0.963 | Ildr1 | NM_134109.1 |
| RIKEN cDNA 4930539E08 | 4930539E08Rik | 0.962 | 9130014G02 | NM_172450.1 |
| syntrophin, basic 2 | Sntb2 | 0.962 | Snt2 | NM_009229.2 |
| SH3-domain GRB2-like 2 | Sh3gl2 | 0.96 | 9530001L19Rik;EEN-B1;Sh3d2a;SH3PA | NM_019535.2 |
| RIKEN cDNA 2900074C18 gene | 2900074C18Rik | 0.96 |  | XM_355870.1 |
| RIKEN cDNA 4933408N05 gene | 4933408N05Rik | 0.96 |  | XM_150119.1 |
| Serine threonine kinase 31 | Stk31 | 0.96 | C330007K24Rik | NM_029916.1 |
| DNA segment, Chr X, Immunex 50 | DXImx50e | 0.958 | AA030924;Sfc25 | NM_207202.1 |
| Testis expressed gene 11 | Tex11 | 0.958 | 4930565P14Rik | NM_031384.1 |
| Lipase, member H | Liph | 0.958 | D16Wsu119e;Lpdlr;mPA-PLA1;PLA1B | NM_153404.1 |
| DNA segment, Chr 11, ERATO Doi 636 | D11Ertd636e | 0.958 | 0610010C04Rik;1500000C01Rik | NM_029794.1 |
| Hypothetical KRAB box containing protein | C430020H24Rik | 0.957 |  | AK049536 |
| ELAV (embryonic lethal, abnormal vision, Drosophila)-like 2 | Elavl2 | 0.956 | Hub;mel-N1 | NM_207686.1 |
| Pleckstrin homology domain containing, family A member 4 | Plekha4 | 0.956 | 2410005C22Rik;PEPP1 | NM_148927.1 |
| PHD finger protein 17 | Phf17 | 0.956 | D530048A03Rik;Jade1;mKIAA1807 | NM_172303.3 |
| Similar to reduced expression 2 | LOC383940 | 0.956 |  | XM_357336.1 |
| Piwi like homolog 2 (Drosophila) | Piwil2 | 0.956 | mili;Piwil1l | NM_021308.1 |
| Williams Beuren syndrome chromosome region 21 | Wbscr21 | 0.956 | 1110054D16Rik | NM_145215.1 |
| RIKEN cDNA 2410088K16 | 2410088K16Rik | 0.954 |  | XM_149067.1 |
| DnaJ (Hsp40) homolog, subfamily C, member 6 | Dnajc6 | 0.954 | 2810027M23Rik;mKIAA0473 | NM_198412.1 |
| Erythrocyte protein band 4.1-like 4a | Epb4.1l4a | 0.954 | Epb4.1l4;NBL4 | NM_013512.1 |
| Similar to AIM2 protein | LOC383619 | 0.954 |  | XM_357160.1 |
| Stromal antigen 3 | Stag3 | 0.954 | SA-2 | NM_016964.1 |
| RIKEN cDNA 2410141K09 | 2410141K09Rik | 0.953 |  | NM_183119.1 |
| Testis expressed gene 19 | Tex19 | 0.953 | 2410081M02Rik | NM_028602.2 |
| Huntingtin-associated protein 1 | Hap1 | 0.953 | HAP-1;MGC31449 | NM_177981.1 |
| RIKEN cDNA A830080D01 | A830080D01Rik | 0.953 |  | XM_356366 |
| Zinc finger protein 295 | Zfp295 | 0.952 | 5430437K12Rik;mKIAA1227;Znf295 | NM_175428.2 |
| Tektin 1 | Tekt1 | 0.952 | MT14 | NM_011569.1 |
| RIKEN cDNA 4930500J02 | 4930500J02Rik | 0.951 |  |  |
| Zinc finger protein 42 | Zfp42 | 0.951 | Rex-1;Rex1;Zfp-42 | NM_009556.2 |
| Activity regulated cytoskeletal-associated protein | Arc | 0.951 | Arc3.1 | NM_018790.1 |
| Similar to tripin | LOC244495 | 0.951 |  | XM_146372.2 |
| Ash2 (absent, small, or homeotic)-like (Drosophila) | Ash2l | 0.951 |  | NM_011791 |
| Actinin alpha 4 | Actn4 | 0.951 |  | NM_021895.2 |
| Similar to Apopolysialoglycoprotein precursor (PSGP) | LOC383616 | 0.95 |  | XM_357157.1 |
| RIKEN cDNA 2310042N02 | 2310042N02Rik | 0.95 | 2310074C17Rik | NM_024246.3 |
| Similar to Apopolysialoglycoprotein precursor (PSGP) | LOC233184 | 0.95 |  | XM_145565.3 |
| Calmegin | Clgn | 0.95 | 4930459O04Rik;Cln | NM_009904 |
| Nuclear factor of activated T-cells, calcineurin-dependent 2 interacting protein | Nfatc2ip | 0.949 | NIP45 | NM_010900.1 |
| RIKEN cDNA D630023F18 | D630023F18Rik | 0.949 | AI314969 | NM_175293.2 |
| RIKEN cDNA 4933425K02 gene | 4933425K02Rik | 0.949 | 1700094G20Rik;4933424B12Rik | NM_028946 |
| cDNA sequence BC035954 | BC035954 | 0.949 | MGC31683 | NM_177569.2 |
| Left-right determination, factor B | Leftb | 0.948 | Lefty;lefty-1;Lefty1;Stra3;Tgfb4 | NM_010094.2 |
| Transcription factor CP2-like 2 | Tcfcp2l2 | 0.948 | Grhl1;LBP-32;MGR | NM_145890.1 |
| G protein-coupled receptor 160 | Gpr160 | 0.948 |  | XM_130823.3 |
| Erythrocyte protein band 4.1-like 4b | Epb4.1l4b | 0.948 | D4Ertd346e;Ehm2 | NM_019427 |
| A kinase (PRKA) anchor protein 1 | Akap1 | 0.948 | Akap;C76494;C81186;S-AKAP84 | NM_009648 |
| RIKEN cDNA 2410146L05 gene | 2410146L05Rik | 0.947 |  | NM_026480.1 |
| Lipase, member H | Liph | 0.947 | ;D16Wsu119e;Lpdlr;mPA-PLA1;PLA1B | NM_153404.1 |
| Tripartite motif protein 2 | Trim2 | 0.947 | narf | NM_030706.1 |
| Protein phosphatase 2a, catalytic subunit, zeta isoform | Ppp2cz | 0.947 | 2310008J22Rik | NM_027982.1 |
| Cyclin-dependent kinase-like 2 (CDC2-related kinase) | Cdkl2 | 0.947 | 5330436L21Rik;KKIAMRE;Kkm | NM_177270.3 |
| 3-hydroxybutyrate dehydrogenase (heart, mitochondrial) | Bdh | 0.946 | 2310032J20Rik | NM_175177.3 |
| Similar to ORF-1137 | LOC386298 | 0.946 |  | XM_359163.1 |
| RIKEN cDNA 1700019H03 | 1700019H03Rik | 0.946 |  |  |
| Fibroblast growth factor binding protein 1 | Fgfbp1 | 0.946 | FGF-BP | NM_008009.2 |
| Glutathione peroxidase 2 | Gpx2 | 0.945 | GPx-GI | NM_030677.1 |
| RIKEN cDNA 9130422G05 | 9130422G05Rik | 0.945 |  | NM_025782 |
| RIKEN cDNA G630064H08 | G630064H08Rik | 0.945 |  | XM_488860 |
| Ectonucleotidepyrophosphatase/phosphodiesterase 3 | ENPP3 | 0.944 |  | AK089553 |
| cDNA sequence BC050188 | BC050188 | 0.944 | 4933403D14 | NM_177742.2 |
| RIKEN cDNA 4921530G04 | 4921530G04Rik | 0.943 |  | NM_027622.1 |
| RIKEN cDNA 2600009P04 | 2600009P04Rik | 0.943 |  |  |
| Autoimmune polyendocrinopathy candidiasis ectodermal dystrophy | Aire | 0.943 |  | NM_009646.1 |
| RIKEN cDNA 2410116G06 | 2410116G06Rik | 0.942 |  | NM_026630.1 |
| Growth differentiation factor 3 | Gdf3 | 0.942 | Gdf-3;Vgr-2;Vgr2 | NM_008108.1 |
| RIKEN cDNA 2410004F06 | 2410004F06Rik | 0.942 | 2410070K17Rik | NM_028034.1 |
| Aldehyde dehydrogenase family 3, subfamily A1 | Aldh3a1 | 0.941 | Ahd-4;Ahd4;Aldh;Aldh3 | NM_007436.1 |
| Leucine-rich repeat-containing 5 | Lrrc5 | 0.94 | 4930525N13Rik;A930019F03 | NM_178701.2 |
| Msx-interacting-zinc finger | Miz1 | 0.94 | 4930520E11Rik;Dib;PIASxb;PIASxbeta;SIZ2 | NM_008602.2 |
| Latent transforming growth factor beta binding protein 4 | Ltbp4 | 0.94 | 2310046A13Rik | NM_175641.1 |
| RIKEN cDNA 2610036A22 | 2610036A22Rik | 0.94 |  |  |
| Transcription elongation factor A (SII), 3 | Tcea3 | 0.939 | S-II | NM_011542 |
| Developmental pluripotency associated 2 | Dppa2 | 0.939 |  | XM_358869.1 |
| Nur77 downstream gene 2 | Ndg2 | 0.939 |  | NM_175329.3 |
| Dehydrogenase/reductase (SDR family) member 6 | Dhrs6 | 0.939 | 1810026B04Rik | NM_027208.1 |
| Dystrophin, muscular dystrophy | Dmd | 0.939 | Dp427;DXSmh7;DXSmh9;mdx;pke | NM_007868.1 |
| RIKEN cDNA 3632413B07 | 3632413B07Rik | 0.938 | 1110013E22Rik;MGC25852;mKIAA1125;Prkcbp1 | NM_172270.1 |
| Ribosomal protein L3-like | Rpl3l | 0.938 | 1110057H16Rik | NM_025425.1 |
| Myeloblastosis oncogene-like 2 | Mybl2 | 0.938 | Bmyb | NM_008652.2 |
| Folate receptor alpha precursor | FR-alpha | 0.938 |  | AF096319 |
| Zinc finger protein 296 | Zfp296 | 0.938 | 2210018A16Rik | NM_022409.1 |
| cDNA sequence BC046418 | BC046418 | 0.938 |  | XM_140553.3 |
| Similar to HYPOTHETICAL PROTEIN ORF-1137 | LOC386199 | 0.938 |  | XM_359116.1 |
| Microtubule-associated protein 7 | Mtap7 | 0.937 | E-MAP-115;MAP7 | NM_008635.1 |
| Estrogen related receptor, beta | Esrrb | 0.937 | Err2;ERRb;Estrrb;Nr3b2 | NM_011934.2 |
| Deoxyribonuclease II alpha | Dnase2a | 0.937 | Dnase2 | NM_010062 |
| Expressed sequence AI118078 | AI118078 | 0.936 | 9630029F15 | NM_172923.1 |
| RIKEN cDNA 1810054D07 | 1810054D07Rik | 0.936 |  | AK007856 |
| Semaphorin) 4B | Sema4b | 0.936 | Semac;SemC | NM_013659.1 |
| Calcium/calmodulin-dependent protein kinase ID | Camk1d | 0.935 | A630059D12Rik;CKLiK;E030025C11Rik | NM_177343 |
| Myeloblastosis oncogene-like 2 | Mybl2 | 0.935 | Bmyb | NM_008652.2 |
| RIKEN cDNA 9130422G05 | 9130422G05Rik | 0.935 |  | NM_025782.2 |
| Similar to HYPOTHETICAL PROTEIN ORF-1137 | LOC386101 | 0.935 |  | XM_359070.1 |
| RIKEN cDNA 4933405K07 | 4933405K07Rik | 0.935 | 4930427I11Rik | NM_028913.1 |
| Keratin complex 1, acidic, gene 17 | Krt1-17 | 0.934 | K17 | NM_010663.1 |
| Zinc finger, SWIM domain containing 1 | Zswim1 | 0.934 | 2410003H12Rik | NM_028028.2 |
| Expressed sequence AU016977 | AU016977 | 0.934 | 9530023G02 | NM_175016.1 |
| RIKEN cDNA 1300001I01 | 1300001I01Rik | 0.933 |  | XM_181333.2 |
| Stromal cell derived factor receptor 2 | Sdfr2 | 0.933 |  | NM_009146 |
| RIKEN cDNA 2310075G12 | 2310075G12Rik | 0.933 | 1110014L05Rik | NM_027162 |
| Zinc finger CCCH type domain containing 1 | Zc3hdc1 | 0.932 | 9930021O16 | NM_172893.1 |
| DNA (cytosine-5-)-methyltransferase 3-like | Dnmt3l | 0.932 | D6Ertd14e | NM_019448.2 |
| Deoxyribonuclease II alpha | Dnase2a | 0.932 | Dnase2 | NM_010062 |
| RIKEN cDNA 2410078J06 | 2410078J06Rik | 0.932 |  | XM_356960.1 |
| RIKEN full-length clone:2610020P14 |  | 0.932 |  | AK011496.1 |
| Testis expressed gene 14 | Tex14 | 0.932 |  | NM_031386.1 |
| Polo-like kinase 3 (Drosophila) | Plk3 | 0.931 | Cnk;Fnk;PRK | NM_013807.1 |
| Thiosulfate sulfurtransferase, mitochondrial | Tst | 0.931 | Rhodanese | NM_009437.2 |
| RIKEN cDNA 4933425K02 | 4933425K02Rik | 0.93 | 1700094G20Rik;4933424B12Rik | AK015318 |
| Engrailed 2 | En2 | 0.93 | En-2 | NM_010134.1 |
| Procollagen, type XVII, alpha 1 | Col17a1 | 0.93 | BP180;Bpag;Bpag2 | NM_007732.1 |
| Growth factor receptor bound protein 2-associated protein 1 | Gab1 | 0.93 |  | NM_021356.2 |
| cDNA sequence BC032203 | BC032203 | 0.93 |  | XM_140041.3 |
| Ubiquitin carboxy-terminal hydrolase L1 | Uchl1 | 0.929 | gad;PGP9.5 | NM_011670.1 |
| RIKEN cDNA 9130227C08 | 9130227C08Rik | 0.929 | 2310014G06Rik;6330555F21;mKIAA0323 | NM_027143 |
| Similar to hypothetical protein FLJ20345 | LOC380718 | 0.929 |  | XM_354636.1 |
| Uroplakin 2 | Upk2 | 0.929 | UPII | NM_009476.1 |
| Phospholipase A2, group VI | Pla2g6 | 0.929 |  | NM_016915.2 |
| RAD9 homolog B (S. cerevisiae) | Rad9b | 0.929 | MGC28469 | NM_144912.1 |
| Msx2 interacting nuclear target protein | Mint | 0.929 | mKIAA0929 | NM_019763.1 |
| NECTIN 4 homolog | 1200017F15Rik | 0.928 |  | AK004821 |
| Meprin 1 beta | Mep1b | 0.928 | Mep-1b | NM_008586.1 |
| Endometrial bleeding associated factor | Ebaf | 0.927 | 6030463A22Rik;Lefta;Lefty2 | NM_177099.3 |
| Tubulin, alpha 3 | Tuba3 | 0.927 | M[a]3 | NM_009446.1 |
| Argininosuccinate synthetase 1 | Ass1 | 0.927 | ASS;Ass-1 | NM_007494.2 |
| RIKEN cDNA 1700012H05 | 1700012H05Rik | 0.926 |  |  |
| Glycosyltransferase 28 domain containing 1 | Glt28d1 | 0.926 |  | NM_026247.1 |
| RAN binding protein 17 | Ranbp17 | 0.926 | 4932704E15Rik | NM_023146.1 |
| Ring finger protein 125 | Rnf125 | 0.925 | 4930553F04Rik | NM_026301.1 |
| UDP-GlcNAc:betaGal beta-1,3-N-acetylglucosaminyltransferase 7 | B3gnt7 | 0.924 | beta-3GnT7;C330001H22Rik | NM_145222.1 |
| Integrin alpha 6 | Itga6 | 0.924 | 5033401O05Rik;Cd49f | NM_008397.2 |
| Cyclin M2 | Cnnm2 | 0.924 | Acdp2 | NM_033569.1 |
| Matrix metalloproteinase 10 | Mmp10 | 0.924 |  | NM_019471.1 |
| RIKEN full-length clone:1700010P22:glutaredoxin 2 (thioltransferase) |  | 0.923 |  | AK005853.1 |
| Similar to HMG-1 | LOC384082 | 0.923 |  | XM_357408.1 |
| Tektin 1 | Tekt1 | 0.923 | MT14 | NM_011569.1 |
| Hypothetical protein 4933409I22 | 4933409I22 | 0.923 |  | NM_172914.1 |
| Ring finger protein 138, transcript variant 2. | Rnf138 | 0.923 | 2410015A17Rik;2810480D20Rik;STRIN;Trif;Trif-d | NM_019706.2 |
| RIKEN cDNA C230081A13 | C230081A13Rik | 0.923 | 9530046P14 | NM_172924 |
| Spectrin beta 3 | Spnb3 | 0.923 |  | XM_129130.5 |
| RIKEN cDNA C430004E15 | C430004E15Rik | 0.923 | C87750 | NM_175286.2 |
| RIKEN cDNA 9630033F20 | 9630033F20Rik | 0.922 |  | NM_177003.2 |
| Microtubule-associated protein 7 | Mtap7 | 0.922 | E-MAP-115;MAP7 | NM_008635 |
| SH3 and cysteine rich domain 2 | Stac2 | 0.922 |  | NM_146028.2 |
| Serine protease inhibitor, Kunitz type 2 | Spint2 | 0.921 | HAI-2 | NM_011464.1 |
| Developmental pluripotency associated 4 | Dppa4 | 0.921 | 2410091M23Rik | NM_028610 |
| Microfibrillar associated protein 5 | Mfap5 | 0.921 | MAGP-2 | NM_015776 |
| Zinc finger protein 459 | Zfp459 | 0.921 |  | NM_177811 |
| Myosin, heavy polypeptide 3 | Myh3 | 0.92 |  | XM_354614.1 |
| DNA segment, Chr 6, Wayne State University 176 | D6Wsu176e | 0.92 |  | NM_138587.3 |
| Dystrobrevin alpha, transcript variant 2. | Dtna | 0.919 | A0;adbn;Dtn | NM_207650.1 |
| Zinc finger protein 219 | Zfp219 | 0.919 | 2010302A17Rik | NM_027248 |
|  | 6330414G02Rik | 0.919 |  |  |
| Similar to zinc finger protein 97 | LOC235860 | 0.918 |  | XM_142431.1 |
| Epoxide hydrolase 2, cytoplasmic | Ephx2 | 0.918 | Eph2 | NM_007940.2 |
| Chromodomain helicase DNA binding protein 4 | Chd4 | 0.918 | 9530019N15Rik;AA617397;D6Ertd380e;MGC11769 | NM_145979.1 |
| RIKEN cDNA D030051N19 | D030051N19Rik | 0.918 | 2310079H06Rik;A130023A14;mKIAA1736 | NM_172669 |
| RIKEN cDNA 2410072D24 | 2410072D24Rik | 0.918 |  | XM_357002.1 |
| Similar to small zinc finger-like protein | LOC223262 | 0.918 |  | XM_127887.1 |
| Solute carrier family 12 (potassium/chloride transporters), member 8 | Slc12a8 | 0.917 | CCC9;E330020C02Rik | NM_134251 |
| Myosin light chain, phosphorylatable, fast skeletal muscle | Mylpf | 0.917 | 2410014J02Rik;MLC-2;Mlc2 | NM_016754.3 |
| RIKEN cDNA 2310003C23 | 2310003C23Rik | 0.917 |  | XM_130746.2 |
| Protein phosphatase 1B, magnesium dependent, beta isoform | Ppm1b | 0.917 | PP2CB | NM_011151.1 |
| Netrin 1 | Ntn1 | 0.917 | Netrin-1 | NM_008744 |
| Inositol 1,3,4-triphosphate 5/6 kinase | Itpk1 | 0.917 |  | NM_172584.1 |
| RIKEN cDNA 9030221M09 | 9030221M09Rik | 0.917 |  | NM_177298.2 |
| Sphingomyelin phosphodiesterase, acid-like 3B | Smpdl3b | 0.917 | 1110054A24Rik;Asml3b | NM_133888.1 |
| Frizzled homolog 5 (Drosophila) | Fzd5 | 0.917 | 5330434N09Rik;Fz5 | NM_022721 |
| RIKEN cDNA 5730592L21 | 5730592L21Rik | 0.916 |  | NM_029720.1 |
| Ring finger protein 17 | Rnf17 | 0.916 | MMIP-2;Mmip2 | AF285585 |
| RIKEN cDNA 2410072D24 | 2410072D24Rik | 0.916 |  | XM_357002 |
| Serum/glucocorticoid regulated kinase | Sgk | 0.916 | Sgk1 | NM_011361 |
| Ataxin 2-like | Atxnl2 | 0.916 |  | NM_183020.1 |
| Nanos homolog 1 | Nanos1 | 0.916 |  | NM_178421.2 |
| RIKEN full-length clone C530043L06 |  | 0.915 |  | AK049709.1 |
| RIKEN cDNA A930012E17 | A930012E17Rik | 0.915 |  | AK044427 |
| UBX domain containing 4 | Ubxd4 | 0.915 | 6330407P03Rik;MGC7992 | NM_145441.2 |
| Hypothetical protein C330048F19 | C330048F19 | 0.915 |  | XM_488799 |
| Similar to hypothetical protein FLJ32191 | LOC195534 | 0.915 |  | XM_112241.3 |
| Polymerase (DNA-directed), epsilon 4 (p12 subunit) | Pole4 | 0.914 | 2400007P05Rik | NM_025882.1 |
| RIKEN cDNA 2310039L15 | 2310039L15Rik | 0.914 |  | XM_290098.1 |
| EF hand domain containing 1 | Efhd1 | 0.914 | 4931430I01Rik;AI452351;PP3051 | NM_028889.1 |
| Integrin, alpha E, epithelial-associated | Itgae | 0.914 | A530055J10;alpha-E1;CD103 | NM_008399.1 |
| Myosin X | Myo10 | 0.913 | D15Ertd600e;mKIAA0799 | NM_019472 |
| Metallothionein 2 | Mt2 | 0.913 | Mt-2;MT-II | NM_008630.1 |
| Similar to zinc finger protein | LOC209182 | 0.913 |  | XM_142725.2 |
| RIKEN cDNA 2310075G12 | 2310075G12Rik | 0.913 | 1110014L05Rik | NM_027162.3 |
| DNA segment, Chr 6, Wayne State University 176 | D6Wsu176e | 0.913 |  | NM_138587.3 |
| Tight junction protein 2 | Tjp2 | 0.913 | ZO-2 | NM_011597 |
| Semaphorin 4A | Sema4a | 0.913 | Semab;SemB | NM_013658.2 |
| RIKEN cDNA 4833439L19 | 4833439L19Rik | 0.912 | 4930558H15Rik;C81457 | NM_133797 |
| Microtubule-associated protein 7 | Mtap7 | 0.912 | E-MAP-115;MAP7 | AK008018 |
| RIKEN cDNA 1700012H05 | 1700012H05Rik | 0.912 |  | XM_133707.3 |
| Glycoprotein A33 (transmembrane) | Gpa33 | 0.912 | 2010310L10Rik;2210401D16Rik | AK008784 |
| Signal transducer and activator of transcription 4 | Stat4 | 0.912 |  | NM_011487.1 |
| Transmembrane protein 20 | Tmem20 | 0.912 | D330039I19Rik | NM_175507.2 |
| Zinc finger protein of the cerebellum 3 | Zic3 | 0.912 | Bn | AK034780 |
| RIKEN cDNA 1700028N11 | 1700028N11Rik | 0.912 |  | NM_029341.1 |
| PHD finger protein 17 | Phf17 | 0.912 | D530048A03Rik;Jade1;mKIAA1807 | NM_172303.3 |
| RIKEN cDNA 1700019G17 | 1700019G17Rik | 0.912 |  | NM_029331.2 |
| Integrin, alpha E, epithelial-associated | Itgae | 0.911 | A530055J10;alpha-E1;CD103 | NM_008399.1 |
| RIKEN cDNA 2210011C24 | 2210011C24Rik | 0.911 |  | XM_356097.1 |
| RIKEN cDNA D230025D16 | D230025D16Rik | 0.911 |  | NM_145604.1 |
| Transmembrane 4 superfamily member 5 | Tm4sf5 | 0.91 | 2010003F10Rik | NM_029360.1 |
| Hydroxysteroid (17-beta) dehydrogenase 1 | Hsd17b1 | 0.91 | 17beta-HSD;E2DH;Hsd17ba | NM_010475.1 |
| Elastase 2 | Ela2 | 0.91 | Ela-2 | NM_007919.1 |
| Topoisomerase (DNA) III beta | Top3b | 0.91 |  | NM_011624.2 |
| RIKEN cDNA A730036I17 | A730036I17Rik | 0.909 |  | NM_177847.2 |
| Chromodomain helicase DNA binding protein 5 | Chd5 | 0.909 |  | XM_196334 |
| Protein tyrosine phosphatase, receptor type, K | Ptprk | 0.909 |  | AK078614 |
| Calmegin | Clgn | 0.909 | 4930459O04Rik;Cln | NM_009904.1 |
| Laminin, alpha 3 | Lama3 | 0.909 |  | XM_128926.3 |
| RIKEN cDNA 1700094G20 | 1700094G20Rik | 0.909 |  | AK007064 |
| RIKEN cDNA 3830431G21 | 3830431G21Rik | 0.909 |  | XM_126991.3 |
| Paired basic amino acid cleaving system 4 | Pace4 | 0.909 |  | XM_355911.1 |
| Protein kinase C, alpha binding protein | Prkcabp | 0.908 | Pick1 | NM_008837.1 |
| Similar to ribosomal protein S4, X-linked | LOC384661 | 0.908 |  | XM_357772.1 |
| RIKEN cDNA 4930529M09 | 4930529M09Rik | 0.908 |  | XM_126016.2 |
| Tumor necrosis factor (ligand) superfamily, member 11 | Tnfsf11 | 0.908 | Ly109l;ODF;OPG;OPGL;RANKL;Trance | NM_011613.2 |
| Similar to HYPOTHETICAL PROTEIN ORF-1137 | LOC270589 | 0.908 |  | XM_193524.2 |
| Branched chain aminotransferase 1, cytosolic | Bcat1 | 0.908 | Bcat-1;BCATc;Eca39 | NM_007532.1 |
| RIKEN cDNA 2410137M14 | 2410137M14Rik | 0.908 |  | NM_029747.1 |
| Cyclin-dependent kinase-like 2 (CDC2-related kinase) | Cdkl2 | 0.908 | 5330436L21Rik;KKIAMRE;Kkm | NM_177270.3 |
| Galactosidase, beta 1 | Glb1 | 0.908 | Bge;Bgl;Bgl-e;Bgl-s;Bgl-t;Bgs;Bgt | AK034049 |
|  | 9530060I07 | 0.907 |  |  |
| Glycosyltransferase 28 domain containing 1 | Glt28d1 | 0.907 |  | NM_026247 |
| Neurogenic differentiation 1 | Neurod1 | 0.907 | BETA2;BHF-1;Neurod | NM_010894.1 |
| Zinc finger protein 532 | Zfp532 | 0.907 |  | NM_207255.1 |
|  | Sox16 | 0.907 |  |  |
| RIKEN cDNA 5730453I16 | 5730453I16Rik | 0.906 | C330017N18Rik;MGC46982 | NM_172302.2 |
| Lactate dehydrogenase 3, C chain, sperm specific | Ldh3 | 0.906 | Ldh-3;Ldhc | NM_013580.2 |
| Similar to KIAA1762 protein | LOC239102 | 0.905 |  | XM_139193.4 |
| Nuclear receptor subfamily 5, group A, member 2 | Nr5a2 | 0.905 | Ftf;LRH-1 | NM_030676.1 |
| Similar to 1700029I01Rik protein | LOC385211 | 0.905 |  | XM_358120.1 |
| Integrin beta 4 | Itgb4 | 0.905 |  | XM_109756.3 |
| Spermatogenesis associated, serine-rich 1 | Spats1 | 0.905 | 1700011H05Rik;4933400B06Rik;Daip2;Srsp1 | NM_027649.1 |
| Receptor (calcitonin) activity modifying protein 3 | Ramp3 | 0.905 |  | NM_019511.1 |
| C-terminal binding protein 2 | Ctbp2 | 0.904 |  | XM_358372.1 |
| Acyloxyacyl hydrolase | Aoah | 0.904 |  | NM_012054.2 |
| Aldehyde dehydrogenase 4 family, member A1 | Aldh4a1 | 0.904 | ALDH4;E330022C09;P5CD;P5CDH;P5CDhL;P5CDhS | NM_175438 |
| Ubiquitin-like 3 | Ubl3 | 0.904 | HCG | NM_011908.1 |
| Diaphanous homolog 1 (Drosophila) | Diap1 | 0.903 | Dia1;Drf1;p140mDia | NM_007858.1 |
| Tight junction protein 2 | Tjp2 | 0.903 | ZO-2 | NM_011597.1 |
| Branched chain aminotransferase 1, cytosolic | Bcat1 | 0.902 | Bcat-1;BCATc;Eca39 | NM_007532 |
| RIKEN cDNA 5830482F20 | 5830482F20Rik | 0.902 |  | NM_177158.2 |
| SWI/SNF-related, matrix-associated actin-dependent regulator of chromatin | Smarcad1 | 0.902 |  | XM_132597.3 |
| RIKEN cDNA 3830422N12 | 3830422N12Rik | 0.902 | 3830422N12;NY-SAR-35;NYSAR35 | NM_174993.1 |
| RIKEN cDNA 9030409E16 | 9030409E16Rik | 0.902 |  | NM_025781.1 |
| RIKEN cDNA 4930461G14 | 4930461G14Rik | 0.902 |  |  |
| Argininosuccinate synthetase 1 | Ass1 | 0.902 | ASS;Ass-1 | NM_007494.2 |
| Ubiquinol-cytochrome c reductase binding protein | Uqcrb | 0.902 | QP-C;QPC;UQBC;UQBP;UQPC | AK017907 |
| DNA segment, Chr 19, ERATO Doi 144 | D19Ertd144e | 0.902 | 5830466O21Rik;Doc-1r | NM_026373.1 |
| Ethanolamine Kinase (EC 2.7.1.82) (EKI) homolog | 4930555L11Rik | 0.902 |  | AK016135 |
| N-ethylmaleimide sensitive fusion protein attachment protein beta | Napb | 0.902 | b-SNAP;Brp14;E161;I47;SNARE | NM_019632.1 |
| Synaptotagmin 4 | Syt4 | 0.901 | SytIV | NM_009308.2 |
| Olfactory receptor 614 | Olfr614 | 0.901 | MOR20-1 | XM_145795.1 |
| Similar to MAP-kinase phosphatase (cpg21) | LOC240672 | 0.901 |  | XM_140740.3 |
| Phospholipase A2, group X | Pla2g10 | 0.901 | mGXsPLA2;PLA2GX;sPLA2-X | XM_148336.1 |
| Formin 2 | Fmn2 | 0.901 |  | NM_019445.1 |
| Zinc finger protein 598 | Zfp598 | 0.9 |  | NM_183149.1 |
| cDNA sequence BC019806 | BC019806 | 0.9 | 2410002F01Rik;MGC30933 | NM_145460 |
