# Supplementary material for: Dynamic transcription programs during ES cell differentiation towards mesoderm in serum versus serum-freeBMP4 culture
Source: BMC Genomics. 2007 Oct 10;8:365. doi: 10.1186/1471-2164-8-365 (PMC2204012; doi:10.1186/1471-2164-8-365)
Supplement: Additional file 4 — Sequence alignment of RIKEN clone 8430415E04RIK. The data provides an alignment of RIKEN clone 8430415E04RIK using human, mouse, chick and zebrafish sequences. [file 1471-2164-8-365-S4.doc]

**Additional file 4:** Sequence alignment of RIKEN clone 8430415E04RIK

human 1 --------------MHPPPPAAAMDFSQNSLFGYMEDLQELTIIERPVRRSLKTPEEIERLTVDEDLSDIERAVYLLSAG
mouse 1 ------------MHPPPPDAGVAMDFGQNSLFGYMEDLQELTIIERPVRRSLKTPEEIERLTVDEDLSDIDRAVYLLSAG
chick 1 AAGGAAAAGGCGASMRPVPCSSDMDFGQSNLFGYIEDLQELTIIERPVRRSLKTPEEIERLTVDEELNDIERALYLLSSG
zebrafish 1 --------------------------SQSSLFGEIDDLQDLTFIERPIRRSLKTAEEIDKLTVDEDLNDIERAVYLLSSG
consensus 1 mhppp a amdf Q sLFGymeDLQeLTiIERPvRRSLKTpEEIerLTVDEdL DIeRAvYLLS G

human 67 QDVQGTSVIANLPFLMRQNPTETLRRVLPKVR---------EALHVAGVEMQLTAAMSFLTILQDESVSIHAYTHSFLQV
mouse 69 QDVQGASVIANLPFLMRQNPTETLRRVLPKVR---------EVLHVASVEMQLTAAVSFLTILQEESMSVHTCAHSFLQV
chick 81 QDVQGTSVVANLPVLMRQNPAETLRRVLPKIR---------EVLHVAGVEMQLTAAVSFLTVLQDESVSIHTYSHSFLHI
zebrafish 55 EDIQRASVIINLPILVRQNPAETFRRVVPKVRVGAPLLPQQEVLHVAGADMQLAAAGSFLTILQDDIVLIQTHTHSILQI
consensus 81 qDvQg SViaNLPfLmRQNP ETlRRVlPKvR EvLHVAgveMQLtAAvSFLTiLQdesvsihtytHSfLqv

human 138 ILLHLEHRDTGVSNAWLETLLSVIEVLPKETLRHEILNPLVSKAQLSQTVQSRLVSCKILGKLTNKFDAHTIKREILPLV
mouse 140 ILLHLEHRDTGVSNAWLETLLSAVELLPKETLRHEILNPLVSKAQLSQTVQSRLVSCKILGKITNKFDAHSIKREILPLV
chick 152 ILQNLEHRDAGVSNAWLETLLAVIEALPKETIRHEILNPLVSKAQISQTLQSRLVSCKIMGKLPNKFEAHIVKREILPLV
zebrafish 135 VLLNLDHRDTVVSSAWLETLLSAIDALPKETIRQEILSPLLSKHQLSQSIPARLASCRILGKVVGKFESSIVKKDLLPLI
consensus 161 iLl LeHRDtgVSnAWLETLLs ieaLPKETlRhEILnPLvSKaQlSQtvqsRLvSCkIlGKltnKFdahiiKreiLPLv

human 218 KSLCQDVEYEVRSCMCRQLENIAQGIGTELTKSVVLPELIELSRDEGSSVRLAAFETLVNLLDIFDTDDRSQTILPLVKS
mouse 220 KSLCQDVEYEVRSCMCRQLENIAQGIGAELTKNVVLPELIELSRDESGSVRLAAFETLVNMLDMFDTDDRSQTILPLVKS
chick 232 KSLCQDVEYEVRTCMCRQLEHVAQGIGTELTKTVVLPELVELARDEGSSVRLAAFETLVNLLDMFDSDDRRQTVLPLVKS
zebrafish 215 RSLCQDVEYEVRACMCRQLENITRGIGLDHTKGEVLPELVELAQDEASTVRLAAFDTIINLLEMFDSDDRTCVIFPLVKA
consensus 241 kSLCQDVEYEVRsCMCRQLEniaqGIGtelTKsvVLPELiEL rDEgssVRLAAFeTlvNlLdmFDtDDRsqtilPLVKs

human 298 FCEKSFKADESILISLSFHLGKLCHGLYGIFTPDQHLRFLEFYKKLCTLGLQQENGHNENQIPPQILEQEKKYISVRKNC
mouse 300 FCEKSFKADESILISLSFHLGKLCHGLYGIFTPDQHLRFLEFYKKLCTLGLQQENGHNESQIPSQIVEQEKKYTSVRKNC
chick 312 FCEKSFKADESILVSLSFHLGKLCNGLYGIFTPEQHLRFLEFYKKLCTLGLQQENGHNDNQLQLQTLEQEKKYISVRKNC
zebrafish 295 FCEKCFKGDEAVLASLSFQYGKLCHGLSESFTDEQHLWFLEFYKKLCCLGLQHENGHCES--QPYPLELENKYALVRRNC
consensus 321 FCEKsFKaDEsiLiSLSFhlGKLChGLygiFTpdQHLrFLEFYKKLCtLGLQqENGHne qi pqilEqEkKYisVRkNC

human 378 AYNFPAMIVFVDPKNFHMELYSTFFCLCHDPEVPVRYTIAICFYEVSKLLNSGVYLIHKELITLLQDESLEVLDALIDHL
mouse 380 AYNFPAMIVFVDPKNFHMELYSTFFCLCHDPEVPVRHTIAICFYEVSKLLNSGVHLIHKELITLLQDESLEVLDALINHL
chick 392 AYNFPAMIVFVDPKNFHLELYSIFFCLCHDPEVPVRYTMAISFYEVAKLLNSGVYTIQKELVTLLQDESLEVLDALVGHL
zebrafish 373 AYNFPAIVLFADPNHFLSELYRTFSSLCHDPEISVRRTAAGGFHEVVKLLGPNVHYIHKELITLLQDDSLEVLDALLNNL
consensus 401 AYNFPAmivFvDPknFhmELYstFfcLCHDPEvpVRyTiAicFyEVsKLLnsgV lIhKELiTLLQDeSLEVLDALinhL

human 458 PEILELMSTGGESSVQENKLSSLPDLIPALTAAEQRAAASLKWRTHEKLLQKYACLPHVISSDQIYYRFLQRMFTIMMTN
mouse 460 PEILELMSTGGENSVQENKFSSVPDLIPALTAAEQRAAASLKWRTHEKLLQKYTCLPHIISSDQIYYRFLQRMFTIMMTN
chick 472 PEILELMINGGENNGSESKLLSIPDLISALTTAEQRAATSLKWRTHEKLLQKYACLPQILSSDQIYYRFLHRMLTIILTN
zebrafish 453 QETLELATSRGEGAGPESKQVNIQDLVPALVAAEQKAASSLHWRVHEKLLQCYSCLPRVISGDQIYFRFFQRMFSIITTN
consensus 481 pEiLELmstgGEns qE KlssipDLipALtaAEQrAAaSLkWRtHEKLLQkYaCLPhviSsDQIYyRFlqRMftImmTN

human 538 NVLPVQKAASRTLCIFLRYNRKQEQRHEVIQKLIEQLGQGKSYWNRLRFLDTCEFIIEIFSKSFFCKYFFLPAIELTHDP
mouse 540 NVLPVQRAAARTLCIFLRYNRKQEQRHEVIQKLIEQLGQGKSYWNRLRFLDTCEFIIEIFSRSFFCKYFFLPVIELTHDP
chick 552 NVLPVQKAAARTLCVYLRYNRKQEQRHEVIQKLIEQLGQGKSYWNRLRFLDTCEFIMELFSKSFFCKYFFLPVLELTHDP
zebrafish 533 NVLPVQREAVRTLCVFLRYNRKQEQRQEIMSKIKQDLAQGRSYWNRLRFLDLCDITIDLFSKSYFCK-------------
consensus 561 NVLPVQkaAaRTLCifLRYNRKQEQRhEviqKlieqLgQGkSYWNRLRFLDtCefiieiFSkSfFCKyfflpvielthdp

human 618 VANVR-MKLCYLLPKVKSTLKIPADKHLLQQLEMCVRKLLCQEKDKDVLAIVKRTVLELDRMEMSMDAFQKKFYEKDLLD
mouse 620 VANVR-MKLCYLLPKVKSALKIPADMHLLQQLEMCVRKLLCQEKDKDVLAIVKKTVLELDRMEMSMDMFQKKNYEKDLLD
chick 632 VANVSRIKLCYLLPKVKSTLKIPTDKHLLQQLELCIRKLLCQEKDKDVLTIVKRTVLELDRMDISLDAFQKRFYENDLLD
zebrafish --------------------------------------------------------------------------------
consensus 641 vanvr mklcyllpkvkstlkipadkhllqqlemcvrkllcqekdkdvlaivkrtvleldrmemsmdafqkkfyekdlld

human 697 QEKEREELLLLEMEQLEKEKQQNDGRPMSDKMFEKKRRDTKTPTQSLPKNIPISVPGPSSVTPSTSKEIKKSKLIRSQSF
mouse 699 QEKEREELLFLEMEQLEKEKHQSDGRLASDKSFEKKRRDSRTSTQSLSKNLPISVPGPSSSTASTSKEIKKSKLTRSQSF
chick 712 QEKERQEHLLLEMEQLEKEKQQNEGRSANVNDKIFEKKRRDNKTSSVLAKSMTLTSSGSSSCTSTGKEDKKSKLVRSQSF
zebrafish --------------------------------------------------------------------------------
consensus 721 qekereellllemeqlekekqqndgr asdk fekkrrdtkt tqsl knipisvpgpssst stskeikksklirsqsf

human 777 NNQAFHAKYGNLEKCASKSSTTGYTTSVSGLGKTSVLSLADDSFRTRNASSVPSSFSPNTPLPSTSRGTGNSVDPKSSGS
mouse 779 NNQAFHAKYGTLDKCASKSSTLAHTSSVSGLVRTAMLSLTDDSFRTRNASSVPASFSPNPVMPSTSRGPGNTADPKSSGS
chick 792 STQALHPKYSNIDKCSNKSSATGYTSSLSGMGKGCMLSFSDDSFRTRSTGNSGNATFSSSSSLIASRNSFNSADQKNNGN
zebrafish --------------------------------------------------------------------------------
consensus 801 nnqafhakygnldkcaskssttgytssvsglgkt mlsltddsfrtrnassvp sfspnt lpstsrgtgnsadpkssgs

human 857 KDTQPRKATLKSRKSNP-
mouse 859 KDAQPRKATLKSRKSNP-
chick 872 KESQSRKMSMVVFCKNQP
zebrafish ------------------
consensus 881 kdtqprkatlksrksnp
