# Supplementary material for: Dynamic transcription programs during ES cell differentiation towards mesoderm in serum versus serum-freeBMP4 culture
Source: BMC Genomics. 2007 Oct 10;8:365. doi: 10.1186/1471-2164-8-365 (PMC2204012; doi:10.1186/1471-2164-8-365)
Supplement: Additional file 5 — FGF5 gene list. The data provided lists all genes expressed during 16 days of embryoid body differentiation with similarity to FGF5 (Pearson correlation >0.9). [file 1471-2164-8-365-S5.doc]

**Additional file 5:** FGF5 (epiblast) gene list (Pearson correlation >0.9)

| **Description** | **Symbol** | **Corr.** | **Synonyms** | **Genbank ID** |
| --- | --- | --- | --- | --- |
| Fibroblast growth factor 5 | Fgf5 | 1 | angora;Fgf-5;go | NM_010203.2 |
| Semaphorin 6A | Sema6a | 0.983 | A730020P05Rik;sema;Sema6A-1;Semaq;VIa | AK042751 |
| Tandem PH domain containing protein-1 |  | 0.979 |  | AK083340.1 |
| C130076O07Rik | C130076O07Rik | 0.973 | C030017F07Rik;mKIAA0343;NrCAM | NM_176930.2 |
| Serine/threonine kinase 32A | Stk32a | 0.97 |  | NM_178749 |
| Eph receptor A1 | Epha1 | 0.969 | 5730453L17Rik;Eph;Esk | NM_023580.2 |
| Zic family member 2 | Zic2 | 0.969 | HPE5;Ku | NM_009574.2 |
| Interferon regulatory factor 1 | Irf1 | 0.969 | Irf-1 | NM_008390.1 |
| Neurofascin | D430023G06Rik | 0.968 |  | AK052440 |
| B430217B02Rik | Irf1 | 0.962 | Irf-1 | NM_008390.1 |
| RIKEN cDNA E130318A13 | E130318A13Rik | 0.961 |  | AK053879 |
| Membrane associated guanylate kinase interacting protein-like 1 | Magi1 | 0.959 | 6820402C05;MGC37681 | NM_172546.1 |
| Poliovirus receptor-related 1 | Pvrl1 | 0.958 | Cd111;HIgR;HveC;nectin-1;PRR;PRR1 | NM_021424.2 |
| Proviral integration site 2 | Pim2 | 0.957 | DXCch3;Pim-2 | NM_138606.1 |
| Wingless-related MMTV integration site 8A | Wnt8a | 0.957 | Stra11;Wnt-8A;Wnt-8D;Wnt8d | NM_009290.1 |
| Eph receptor A1 | Epha1 | 0.957 | 5730453L17Rik;Eph;Esk | NM_023580.2 |
| Glutathione S-transferase, theta 2 | Gstt2 | 0.951 | mGSTT2;Yrs | NM_010361.1 |
| Apical protein, Xenopus laevis-like | Apxl | 0.949 | 4832440C16;C630003H05Rik | NM_172441.1 |
| RIKEN cDNA 1810005K13 | 1810005K13Rik | 0.949 |  |  |
| Kinesin family member 1A | Kif1a | 0.948 | ATSV;Kns1 | NM_008440.1 |
| Psoriasis susceptibility 1 candidate 2 | Psors1c2 | 0.942 | Pcg;SPR1 | NM_020576.1 |
| UDP-N-acetyl-alpha-D-galactosamine:N-cetylgalactosaminyltransferase 3 | Galnt3 | 0.941 |  | AK087219 |
| Rap guanine nucleotide exchange factor (GEF) 5 | Rapgef5 | 0.937 | GFR;mKIAA0277;mmr-gef;Mrgef | NM_175930.2 |
| Dual specificity phosphatase 6 | Dusp6 | 0.935 | 1300019I03Rik;MKP-3;MKP3;PYST1 | NM_026268.1 |
| Interferon regulatory factor 1 | Irf1 | 0.932 | Irf-1 | NM_008390.1 |
| Adaptor protein complex AP-1, mu 2 subunit | Ap1m2 | 0.931 | [m]1B;D9Ertd818e;mu1B | NM_009678.1 |
| Sulfotransferase family, cytosolic, 2B, member 1 | Sult2b1 | 0.928 | SULT2B | NM_017465.1 |
| Cofactor required for Sp1 transcriptional activation subunit 2 | Crsp2 | 0.927 |  | NM_012005 |
| Similar to hypothetical protein DKFZp434P0316 | LOC217341 | 0.925 |  | XM_126800.2 |
| RIKEN cDNA 1700067G17 | 1700067G17Rik | 0.925 |  |  |
| RIKEN cDNA 2810025M15 | 2810025M15Rik | 0.924 |  | NM_027274.2 |
| Aminoadipate aminotransferase | Aadat | 0.924 |  | NM_011834.1 |
| Potassium inwardly-rectifying channel, subfamily J, member 3 | Kcnj3 | 0.923 | GIRK1;Kcnf3;Kir3.1 | NM_008426.1 |
| Zinc finger, SWIM domain containing 5 | Zswim5 | 0.922 |  | XM_204114.3 |
| Semaphorin 6a precursor | C230094A19Rik | 0.922 |  | AK082711 |
| Hypothetical LOC332300 | LOC332300 | 0.92 |  | XM_288828.2 |
| Member RAS oncogene family, transcript variant 1 | Rab26 | 0.919 |  | XM_283428 |
| Crumbs homolog 3 (Drosophila) | Crb3 | 0.919 | 5730439B18 | NM_177638.3 |
| Adaptor protein complex AP-1, mu 2 subunit | Ap1m2 | 0.917 | [m]1B;D9Ertd818e;mu1B | NM_009678.1 |
| Scinderin | Scin | 0.917 | adseverin | NM_009132.1 |
| Y box protein 2 | Ybx2 | 0.915 | Msy2 | NM_016875.1 |
| Glutathione S-transferase, mu 6 | Gstm6 | 0.914 |  | NM_008184.1 |
| Matrix metalloproteinase 25 | Mmp25 | 0.913 |  | XM_139838.2 |
| Expressed in non-metastatic cells 3 | Nme3 | 0.913 | 1810009F08Rik;DR-nm23;Ndk3;Nm23-DR;Nm23-M3 | NM_019730.1 |
| Similar to hypothetical protein FLJ20519 | LOC229588 | 0.911 |  | XM_131066.3 |
| RIKEN cDNA 4930463G05 | 4930463G05Rik | 0.91 | 4632415N18Rik;4930428C11Rik | NM_027559.1 |
| cDNA sequence BC026370 | BC026370 | 0.909 |  | NM_198167.1 |
| RIKEN cDNA C130076O07 | C130076O07Rik | 0.909 | C030017F07Rik;mKIAA0343;NrCAM | XM_147660.1 |
| RIKEN cDNA A930024E05 | A930024E05Rik | 0.909 |  | NM_175405.2 |
| RIKEN cDNA 0610037M15 | 0610037M15Rik | 0.909 |  |  |
| DNA methyltransferase 3B | Dnmt3b | 0.908 |  | AF151973 |
| RIKEN cDNA A430091O22 | A430091O22Rik | 0.908 | 4732493M14 | NM_183024.1 |
| Potassium channel, subfamily K, member 1 | Kcnk1 | 0.908 | TWIK-1 | NM_008430 |
| Proteosome (prosome, macropain) subunit, beta type 9 | Psmb9 | 0.908 | Lmp-2;Lmp2 | NM_013585.1 |
| G protein-coupled receptor 23 | Gpr23 | 0.906 | 5730485F04Rik | NM_175271 |
| WASP family 1 | Wasf1 | 0.906 | Scar;WAVE-1 | NM_031877.2 |
| Hypothetical FAD-dependent pyridine nucleotide-disulphide oxidoreductase | 2810401C16Rik | 0.906 |  | AK012958 |
| Gamma-aminobutyric acid (GABA-A) receptor, subunit beta 3 | Gabrb3 | 0.906 | A230092K12Rik;Cp1;Gabrb-3 | NM_008071.2 |
| Mitogen-activated protein kinase kinase kinase 9 | Map3k9 | 0.904 | E130314H24Rik;Mlk1;Prke1 | NM_177395 |
| Nudix (nucleoside diphosphate linked moiety X)-type motif 8 | Nudt8 | 0.904 | 2310039H17Rik | NM_025529.2 |
| Hypothetical RNA-binding region RNP-1 | A430091O22Rik | 0.904 | 4732493M14 | AK040404 |
| Proprotein convertase subtilisin/kexin type 9 | Pcsk9 | 0.901 | AI415265;FH3;HCHOLA3;MGC47409;Narc1 | NM_153565.1 |
| Hyaluronan and proteoglycan link protein 4 | Hapln4 | 0.901 | 9330174O11;Bral2;Lpr4 | NM_177900.3 |
| Mitogen-activated protein kinase 12 | Mapk12 | 0.901 | Erk6;P38gamma;Prkm12;Sapk3 | NM_013871.2 |
