# Supplementary material for: Dynamic transcription programs during ES cell differentiation towards mesoderm in serum versus serum-freeBMP4 culture
Source: BMC Genomics. 2007 Oct 10;8:365. doi: 10.1186/1471-2164-8-365 (PMC2204012; doi:10.1186/1471-2164-8-365)
Supplement: Additional file 6 — Wnt5a gene list. The data provided lists all genes expressed during 16 days of embryoid body differentiation with similarity to Wnt5 (Pearson correlation >0.9). [file 1471-2164-8-365-S6.doc]

**Additional file 6:** Wnt5a (Late primitive streak) gene list (Pearson correlation >0.9)

| **Description** | **Symbol** | **Corr.** | **Synonyms** | **Genbank ID** |
| --- | --- | --- | --- | --- |
| Wingless-related MMTV integration site 5A | Wnt5a | 1 | 8030457G12Rik;Wnt-5a | NM_009524.2 |
| Homeo box B2 | Hoxb2 | 0.972 | Hox-2.8 | NM_134032.1 |
| LOC380655 | LOC380655 | 0.968 |  | XM_358397.1 |
| T-BOX PROTEIN 3 | 6030495B01Rik | 0.966 |  | AK031708 |
| Ocular development associated gene | Odag | 0.965 | 2310031E19Rik;2810047M21Rik | NM_026033.1 |
| Enolase 3, beta muscle | Eno3 | 0.963 | Eno-3 | NM_007933.2 |
| AB023957 | AB023957 | 0.956 | EIG180 | NM_133237.1 |
| Adrenergic receptor, alpha 2a | Adra2a | 0.956 | alpha2-C10;alpha2A | NM_007417 |
| RIKEN cDNA D230045O07Rik | D230045O07Rik | 0.952 |  | AK052099 |
| Ribosomal protein L36a | Rpl36a | 0.951 | L44L;Rpl44 | NM_019865.2 |
| Similar to IGE-BINDING PROTEIN | E430033B07Rik | 0.95 |  | AK088947 |
| Zinc finger protein 592 | Zfp592 | 0.949 | 8430405N24;A730014M16Rik;mKIAA0211 | NM_178707.2 |
| RIKEN cDNA C530008M17 | C530008M17Rik | 0.948 |  | XM_287460.2 |
| CCR4-NOT transcription complex, subunit 7 | Cnot7 | 0.948 | Caf1;Pop2 | NM_011135.2 |
| Homeo box, msh-like 2 | Msx2 | 0.947 | Hox-8;Hox8;Hox8.1 | NM_013601.1 |
| Nuclear respiratory factor 1 | Nrf1 | 0.946 |  | NM_010938.2 |
| RIKubiquinol cytochrome c reductase core protein 2 | Uqcrc2 | 0.946 | 1500004O06Rik;4930408O21Rik | NM_025899.1 |
| RIKEN cDNA 4432404J10 | 4432404J10Rik | 0.943 | 2010005B09Rik;KIAA1521;mKIAA1521 | NM_025709.2 |
| RIKEN cDNA 1700001A24 | 1700001A24Rik | 0.941 |  | NM_175395.2 |
| START domain containing 8 | Stard8 | 0.94 |  | NM_199018.1 |
| G protein-coupled receptor 73 | Gpr73 | 0.938 | EG-VEGFR1;Pkr1 | NM_021381.3 |
| 4930471O16Rik | 4930471O16Rik | 0.938 |  | NM_026348.2 |
| Forkhead box K1 | Foxk1 | 0.936 | A630048H08Rik;Mnf | NM_010812 |
| Hypothetical protein A230106N23 | A230106N23 | 0.935 |  | NM_177838.2 |
| RIKEN cDNA 2700046A07 | 2700046A07Rik | 0.933 |  | XM_484738 |
| RIKEN cDNA 4933434L15 | 4933434L15Rik | 0.932 |  | NM_026231.1 |
| Dihydrofolate reductase | Dhfr | 0.931 | 8430436I03Rik | NM_010049 |
| Opsin (encephalopsin) | Opn3 | 0.931 | Ecpn;ERO | NM_010098.1 |
| Hoxd1 | Hoxd1 | 0.93 | Hox-4.9 | NM_010467.1 |
| Thrombospondin, type I, domain 2 | Thsd2 | 0.929 | 2810459H04Rik | NM_028351.1 |
| Histocompatibility 2, O region alpha locus | H2-Oa | 0.929 | H-2Oa | NM_008206 |
| Cbp/p300-interacting transactivator, with Glu/Asp-rich carboxy-terminal domain | Cited2 | 0.928 | ER154-like;Mrg1;Msg2;p35srj | NM_010828.1 |
| NAD(P) dependent steroid dehydrogenase-like | Nsdhl | 0.926 | Bpa;H105E3;Str;XAP104 | NM_010941.3 |
| Bone morphogenetic protein 4 | Bmp4 | 0.926 | Bmp2b;Bmp2b-1;Bmp2b1 | NM_007554.1 |
| Sox7 | Sox7 | 0.925 |  | NM_011446.1 |
| Casein kinase II, alpha 2, polypeptide | Csnk2a2 | 0.925 | 1110035J23Rik;CK2 | NM_009974.2 |
| Fucosyltransferase 4 | Fut4 | 0.925 | FAL;FucT-IV | NM_010242.2 |
| RIKEN cDNA 5930412G12 | 5930412G12Rik | 0.923 |  |  |
| RIKEN cDNA 4932442C03 | 4932442C03Rik | 0.921 |  | AK030106 |
| PET112-like (yeast) | Pet112l | 0.921 | 9430026F02Rik;MGC11629 | NM_144896.2 |
| T-box 3 | Tbx3 | 0.921 | D5Ertd189e | NM_011535.2 |
| Menage a trois 1 | Mnat1 | 0.92 | MAT1;P36 | NM_008612.1 |
| RIKEN cDNA 2700069A02 | 2700069A02Rik | 0.92 |  | AK076066 |
| Hematopoietically expressed homeobox | Hhex | 0.92 | Hex;Prh;Prhx | NM_008245.2 |
| Cripto, FRL-1, cryptic family 1 | Cfc1 | 0.917 | cryptic | NM_007685.1 |
| ISL1 transcription factor, LIM/homeodomain (islet 1) | Isl1 | 0.916 |  | NM_021459.2 |
| FMS-like tyrosine kinase 1 | Flt1 | 0.916 | Flt-1;sFlt1;VEGFR-1;VEGFR1 | NM_010228.2 |
| Hoxb6 | Hoxb6 | 0.916 | Hox-2.2 | NM_008269.1 |
| BTB and CNC homology 2 | Bach2 | 0.916 |  | NM_007521.2 |
| HMG4 | LOC270665 | 0.914 |  | XM_195245.2 |
| RIKEN cDNA 4632404H22 | 4632404H22Rik | 0.914 |  | NM_030167.1 |
| RIKEN cDNA C030018P15 | C030018P15Rik | 0.913 |  |  |
| RIKEN cDNA 0610009F02 | 0610009F02Rik | 0.913 |  | NM_029508.1 |
| NAD(P) dependent steroid dehydrogenase-like | Nsdhl | 0.912 | Bpa;H105E3;Str;XAP104 | NM_010941.3 |
| ADP-ribosylation factor-like 2 binding protein | Arl2bp | 0.912 | 1700010P10Rik;1700027H16Rik;6330544B05Rik;Bart1 | NM_024191.1 |
| Protein-L-isoaspartate (D-aspartate) O-methyltransferase 1 | Pcmt1 | 0.91 | PIMT | NM_008786.1 |
| Mitochondrial ribosomal protein L23 | Mrpl23 | 0.91 | L23mrp;Rpl23;Rpl23l | NM_011288.1 |
| Heart and neural crest derivatives expressed transcript 1 | Hand1 | 0.91 | eHAND;Ehand1;Hxt;Th1;Thing1 | NM_008213.1 |
| SMC (structural maintenance of chromosomes 1)-like 1 | Smc1l1 | 0.91 | 5830426I24Rik;SB1.8;Smc1;Smc1alpha;SmcB | NM_019710.1 |
| Spindlin-like protein 2 | LOC278240 | 0.909 |  | XM_204445.1 |
| Pellino 1 | Peli1 | 0.908 | 2810468L03Rik;A930031K15Rik;D11Ertd676e | NM_030015.1 |
| RIKEN cDNA 1810044O22 | 1810044O22Rik | 0.908 | Cyb5m | NM_025558.2 |
| NAD(P) dependent steroid dehydrogenase-like | Nsdhl | 0.908 | Bpa;H105E3;Str;XAP104 | NM_010941.3 |
| Keratin complex 2, basic, gene 8 | Krt2-8 | 0.907 | Card2;EndoA;K8;Krt-2.8 | NM_031170.1 |
| RIKEN cDNA 2810027O19 | 2810027O19Rik | 0.907 | 4930522P10Rik | NM_026613.2 |
| Caudal type homeo box 2 | Cdx2 | 0.906 | Cdx-2 | NM_007673.2 |
| Chloride intracellular channel 4 (mitochondrial) | Clic4 | 0.905 | mc3s5;mtCLIC | XM_124389.1 |
| Acetyl-Coenzyme A acetyltransferase 2 | Acat2 | 0.905 | Tcp-1x;Tcp1-rs1 | NM_009338 |
| RIKEN cDNA 1810011E08 | 1810011E08Rik | 0.904 |  |  |
| Par-3 | Pard3 | 0.903 | ASIP;D8Ertd580e;Par3 | NM_033620.1 |
| RIKEN cDNA B130055L09 | B130055L09Rik | 0.903 | B130055L09 | NM_172467.1 |
| NADH dehydrogenase 1 alpha subcomplex, 9 | Ndufa9 | 0.903 | 1010001N11Rik | NM_025358.1 |
| Regulator of G-protein signaling 5 | Rgs5 | 0.902 | 1110070A02Rik | NM_009063.2 |
| Vomeronasal 1 receptor, E11 | V1re11 | 0.902 |  | NM_134230.1 |
