# Supplementary material for: Dynamic transcription programs during ES cell differentiation towards mesoderm in serum versus serum-freeBMP4 culture
Source: BMC Genomics. 2007 Oct 10;8:365. doi: 10.1186/1471-2164-8-365 (PMC2204012; doi:10.1186/1471-2164-8-365)
Supplement: Additional file 7 — Position Weighted Matrices. Provides published data on position weighted matrices for Oct, Sox, KLF-A, KLF4, Nanog, E-box, Gata-1 and their variants. [file 1471-2164-8-365-S7.doc]

**Additional file 7:** Position Weighted Matrices

KLF-A

| Residue | A | C | G | T | Consensus |
| --- | --- | --- | --- | --- | --- |
| 1 | 0 | 100 | 0 | 0 | C |
| 2 | 0 | 100 | 0 | 0 | C |
| 3 | 35 | 35 | 15 | 15 | A/T |
| 4 | 0 | 100 | 0 | 0 | C |
| 5 | 70 | 15 | 15 | 0 | A |
| 6 | 0 | 100 | 0 | 0 | C |
| 7 | 0 | 100 | 0 | 0 | C |
| 8 | 0 | 100 | 0 | 0 | C |
| 9 | 40 | 10 | 10 | 40 | A/T |

Octamer

| Residue | A | C | G | T | Consensus |
| --- | --- | --- | --- | --- | --- |
| 1 | 100 | 0 | 0 | 0 | A |
| 2 | 0 | 0 | 0 | 100 | T |
| 3 | 0 | 0 | 100 | 0 | G |
| 4 | 0 | 100 | 0 | 0 | C |
| 5 | 50 | 0 | 0 | 50 | A/T |
| 6 | 100 | 0 | 0 | 0 | A |
| 7 | 100 | 0 | 0 | 0 | A |
| 8 | 0 | 0 | 0 | 100 | T |

Extended Octamer- Loh et al. 2006

| Residue | A | C | G | T | Consensus |
| --- | --- | --- | --- | --- | --- |
| 1 | 67 | 1 | 2 | 30 | A |
| 2 | 2 | 2 | 0 | 96 | T |
| 3 | 1 | 10 | 0 | 89 | T |
| 4 | 0 | 31 | 67 | 2 | G |
| 5 | 34 | 4 | 1 | 61 | T |
| 6 | 4 | 44 | 35 | 17 | C |
| 7 | 60 | 2 | 3 | 35 | A |
| 8 | 0 | 0 | 2 | 98 | T |
| 9 | 2 | 0 | 75 | 23 | G |
| 10 | 15 | 70 | 7 | 8 | C |
| 11 | 46 | 4 | 0 | 50 | A/T |
| 12 | 75 | 3 | 22 | 1 | A |
| 13 | 80 | 1 | 15 | 4 | A |
| 14 | 30 | 3 | 7 | 60 | T |
| 15 | 17 | 27 | 38 | 18 | G |

Oct3/4-Sox2

| Residue | A | C | G | T | Consensus |
| --- | --- | --- | --- | --- | --- |
| 1 | 50 | 0 | 0 | 50 | A/T |
| 2 | 50 | 0 | 0 | 50 | A/T |
| 3 | 50 | 0 | 0 | 50 | A/T |
| 4 | 50 | 0 | 0 | 50 | A/T |
| 5 | 0 | 0 | 100 | 0 | G |
| 6 | 0 | 100 | 0 | 0 | C |
| 7 | 100 | 0 | 0 | 0 | A |
| 8 | 0 | 0 | 0 | 100 | T |
| 9 | 25 | 25 | 25 | 25 | N |
| 10 | 25 | 25 | 25 | 25 | N |
| 11 | 25 | 25 | 25 | 25 | N |
| 12 | 50 | 0 | 0 | 50 | A/T |
| 13 | 100 | 0 | 0 | 0 | A |
| 14 | 0 | 100 | 0 | 0 | C |
| 15 | 100 | 0 | 0 | 0 | A |
| 16 | 100 | 0 | 0 | 0 | A |
| 17 | 50 | 0 | 0 | 50 | A/T |
| 18 | 0 | 0 | 100 | 0 | G |

KLF4- Shields and Yang, 1998

| Residue | A | C | G | T | Consensus |
| --- | --- | --- | --- | --- | --- |
| 1 | 24 | 7 | 13 | 2 | A |
| 2 | 26 | 6 | 9 | 5 | A |
| 3 | 28 | 6 | 6 | 6 | A |
| 4 | 17 | 2 | 17 | 10 | A/G |
| 5 | 26 | 4 | 15 | 1 | A |
| 6 | 27 | 3 | 13 | 3 | A |
| 7 | 23 | 3 | 16 | 4 | A |
| 8 | 22 | 0 | 22 | 2 | A/G |
| 9 | 21 | 2 | 22 | 1 | A/G |
| 10 | 28 | 3 | 11 | 4 | A |
| 11 | 24 | 1 | 16 | 5 | A |
| 12 | 18 | 0 | 37 | 1 | G |
| 13 | 0 | 0 | 46 | 0 | G |
| 14 | 0 | 0 | 46 | 0 | G |

Nanog - Loh et al. 2006

| Residue | A | C | G | T | Consensus |
| --- | --- | --- | --- | --- | --- |
| 1 | 20 | 15 | 55 | 10 | G |
| 2 | 21 | 12 | 61 | 6 | G |
| 3 | 24 | 1 | 74 | 1 | G |
| 4 | 34 | 36 | 29 | 1 | C |
| 5 | 8 | 62 | 0 | 29 | C |
| 6 | 1 | 98 | 1 | 0 | C |
| 7 | 98 | 1 | 1 | 0 | A |
| 8 | 3 | 0 | 3 | 94 | T |
| 9 | 0 | 1 | 0 | 99 | T |
| 10 | 16 | 2 | 33 | 49 | T |
| 11 | 20 | 62 | 10 | 8 | C |
| 12 | 0 | 100 | 0 | 0 | C |

Nanog-Mitsui et al. 2003

| Residue | A | C | G | T | Consensus |
| --- | --- | --- | --- | --- | --- |
| 1 | 0 | 5 | 5 | 0 | C/G |
| 2 | 5 | 0 | 5 | 0 | A/G |
| 3 | 0 | 5 | 5 | 0 | C/G |
| 4 | 0 | 10 | 0 | 0 | C |
| 5 | 0 | 5 | 5 | 0 | C/G |
| 6 | 10 | 0 | 0 | 0 | A |
| 7 | 0 | 0 | 0 | 10 | T |
| 8 | 0 | 0 | 0 | 10 | T |
| 9 | 10 | 0 | 0 | 0 | A |
| 10 | 2 | 2 | 2 | 2 | N |
| 11 | 0 | 5 | 5 | 0 | C/G |

E-Box (From JASPAR)

| Residue | A | C | G | T | Consensus |
| --- | --- | --- | --- | --- | --- |
| 1 | 0 | 100 | 0 | 0 | C |
| 2 | 100 | 0 | 0 | 0 | A |
| 3 | 0 | 0 | 100 | 0 | G |
| 4 | 0 | 0 | 100 | 0 | G |
| 5 | 0 | 0 | 0 | 100 | T |
| 6 | 0 | 0 | 100 | 0 | G |

Gata1- Merika et al. 1993

| Residue | A | C | G | T | Consensus |
| --- | --- | --- | --- | --- | --- |
| 1 | 0 | 0 | 50 | 0 | G |
| 2 | 50 | 0 | 0 | 0 | A |
| 3 | 0 | 0 | 0 | 50 | T |
| 4 | 18 | 16 | 18 | 11 | A/T |
| 5 | 5 | 14 | 24 | 9 | G |
