# Supplementary material for: Dynamic transcription programs during ES cell differentiation towards mesoderm in serum versus serum-freeBMP4 culture
Source: BMC Genomics. 2007 Oct 10;8:365. doi: 10.1186/1471-2164-8-365 (PMC2204012; doi:10.1186/1471-2164-8-365)
Supplement: Additional file 8 — Oligonucleotide sequences used for qRT-PCR analysis. Lists the oligonucleotide sequences and cycling conditions used for the qRT-PCR analysis. [file 1471-2164-8-365-S8.doc]

**Additional file 8:** Oligonucleotide sequences and conditions used for qRT-PCR analysis.

| Gene | **5' Sequence (5'-3')** | **3' Sequence (5'-3')** | **(bp)** | **Ta (^0^C)** |
| --- | --- | --- | --- | --- |
| α-Globin | -gtcacggcaagaaggtcgc- | -ggggtgaaatcggcaggg- | 321 | 52 |
| β-h1 globin | -gaaggctcctgattgtttaccca- | -acatgttgcccaggagcttg- | 243 | 60 |
| Actc 1 | -tccagccctctttcattggta- | -gagcccccaatccagaca- | 247 | 60 |
| Brachyury | -gataactggtctagcctcggagtg- | -ccagagactgggatactggctaga- | 102 | 60 |
| Cdx4 | -ggttaccctcatatggtccaacat- | -ccgccgttgcttgca- | 209 | 60 |
| 2410146L05Rik | -gcagaaagggtcataggcaca- | -tgaggaggcacgaattttca- | 249 | 60 |
| 8430415E04Rik | -ttgatgcgacagaatcctactga- | -gcttaattctactgcggagagaag- | 253 | 60 |
| HPRT | -gcagtacagccccaaaatgg- | -aacaaagtctggcctgtatccaa- | 85 | 60 |
| Klf2 | -ccacacatacttgcagctacacc- | -ctttcggtagtggcgggtaa- | 167 | 59 |
| Klf3 | -gggaagagacctctacctgtgg- | -catttgtacggcttttctcctg- | 152 | 59 |
| Klf4 | -tatgcaggctgtggcaaaac- | -tttgcggtagtgcctggtc- | 150 | 59 |
| Klf5 | -tgcttccaaactggcgatt- | -cgtatgagtcctcaggtgagctt- | 199 | 59 |
| Klf9 | -ctacagtggctgtgggaaagtc- | -cttggtgagatggtcactcctc- | 223 | 59 |
| Klf16 | -acctctccggtctcctcctc- | -ggcgaacttcttgtcacagc- | 201 | 59 |
| Lim1 | -ggaggagctggttgtcttcaaaga- | -gcgacactgctgttactcaggta- | 458 | 60 |
| Mixl1 | -ggagctcgtcttccgacaga- | -ttgaggataagggctgaaatgac- | 161 | 60 |
| Myl4 | -acatctcccgcaacaaggag- | -tgatgtgcttgacaaaggcttc- | 219 | 60 |
| Myl7 | -tattcccagctcggtgaggta- | -gaacttgtctgcctgggtca- | 247 | 60 |
| Nanog | -cagaaaaaccagtggttgaagactag- | -gcaatggatgctgggatactc- | 82 | 61 |
| Oct4/Pou5f1 | -caactcccgaggagtccca- | -ctgggtgtaccccaaggtga- | 101 | 60 |
| Podocalyxin | -ccagaggaaggaccagcaa- | -caccttcttctcctgcatct- | 120 | 60 |
| WT1 | -cttccgaggcattcaggatgt- | -ccggctatgcatctgtaagtgg- | 157 | 60 |

bp- amplicon size in base pairs.

Ta- Annealing temperature.
